# World Allergy Organization (WAO) Diagnosis and Rationale for Action against Cow’s Milk Allergy (DRACMA) Guideline update – XIV – Recommendations on CMA immunotherapy

**DOI:** 10.1016/j.waojou.2022.100646

**Published:** 2022-04-23

**Authors:** Jan L. Brozek, Ramon T. Firmino, Antonio Bognanni, Stefania Arasi, Ignacio Ansotegui, Amal H. Assa'ad, Sami L. Bahna, Roberto Berni Canani, Martin Bozzola, Derek K. Chu, Lamia Dahdah, Christophe Dupont, Piotr Dziechciarz, Motohiro Ebisawa, Elena Galli, Andrea Horvath, Rose Kamenwa, Gideon Lack, Haiqi Li, Alberto Martelli, Anna Nowak-Węgrzyn, Nikolaos G. Papadopoulos, Ruby Pawankar, Yetiani Roldan, Maria Said, Mario Sánchez-Borges, Raanan Shamir, Jonathan M. Spergel, Hania Szajewska, Luigi Terracciano, Yvan Vandenplas, Carina Venter, Siw Waffenschmidt, Susan Waserman, Amena Warner, Gary W.K. Wong, Alessandro Fiocchi, Holger J. Schünemann

**Affiliations:** a Department of Health Research Methods, Evidence & Impact, McMaster University, Hamilton, Ontario, Canada.; b Department of Medicine, Division of Clinical Immunology and Allergy, McMaster University, Hamilton, Ontario, Canada; c Faculty of Medical Sciences of Campina Grande, UNIFACISA University Centre, Campina Grande, Paraiba, Brazil; d Division of Allergy, Bambino Gesù Children's Hospital, Rome, Italy; e Hospital Quironsalud Bizkaia, Bilbao-Erandio, Spain; f Division of Allergy and Immunology, Cincinnati Children's Hospital Medical Center, Cincinnati, OH, USA; g Allergy and Immunology Section, Louisiana State University Health Sciences Center, Shreveport, LA, USA; h Pediatric Allergy Program at the Department of Translational Medical Science, and ImmunoNutritionLab at Ceinge Advanced Biotechnologies, University of Naples Federico II, Naples, Italy; i Department of Pediatrics, British Hospital-Perdriel, Buenos Aires, Argentina; j Paris Descartes University, Pediatric Gastroenterology, Necker Hospital, Paris, France; k Clinique Marcel Sembat, Boulogne-Billancourt, France; l Department of Paediatrics, Medical University of Warsaw, Warsaw, Poland; m Clinical Research Center for Allergy and Rheumatology, National Hospital Organization, Sagamihara National Hospital, Kanagawa, Japan; n Pediatric Allergy Unit, San Pietro Hospital – Fatebenefratelli, Rome, Italy; o Department of Paediatrics and Child Health, Aga Khan University Hospital, Nairobi, Kenya; p King's College London, Asthma-UK Centre in Allergic Mechanisms of Asthma, Department of Pediatric Allergy, St Thomas' Hospital, London, UK; q Department of Primary Child Care, Children's Hospital, Chongqing Medical University, China; r Italian Society od Pediatric Allergy and Immunology; s Department of Pediatrics, NYU Grossman School of Medicine, Hassenfeld Children's Hospital, New York, NY, USA; t Department of Pediatrics, Gastroenterology and Nutrition, Collegium Medicum, University of Warmia and Mazury, Olsztyn, Poland; u Division of Infection, Immunity and Respiratory Medicine, School of Biological Sciences, University of Manchester, Manchester, UK; v Allergy Department, 2nd Paediatric Clinic, National and Kapodistrian University of Athens, Athens, Greece; w Division of Allergy, Department of Pediatrics, Nippon Medical School, Tokyo, Japan; x Allergy & Anaphylaxis Australia, Castle Hill, New South Wales, Australia; y Allergy and Clinical Immunology Department, Centro Médico Docente La Trinidad and Clínica El Avila, Caracas, Venezuela; z Institute for Gastroenterology, Nutrition and Liver Diseases, Schneider Children's Medical Center of Israel, Sackler Faculty of Medicine, Tel Aviv University, Tel Aviv, Israel; aa Division of Allergy and Immunology, The Children's Hospital of Philadelphia, University of Pennsylvania School of Medicine, Philadelphia, PA, USA; ab Pediatric Primary Care, National Pediatric Health Care System, Milan, Italy; ac Department of Pediatric Gastroenterology, Universitair Ziekenhuis Brussel, Brussels, Belgium; ad Section of Allergy and Immunology, Children's Hospital Colorado, University of Colorado School of Medicine, Aurora, CO, USA; ae Institute for Quality and Efficiency in Health Care, Cologne, Germany; af Allergy UK, London, England, UK; ag Department of Paediatrics, Prince of Wales Hospital, Chinese University of Hong Kong, Shatin, Hong Kong; ah Department of Medicine, Division of Internal Medicine, McMaster University, Hamilton, Ontario, Canada

**Keywords:** Milk allergy, Immunotherapy, Practice guidelines, GRADE

## Abstract

**Background:**

The prevalence of cow's milk allergy (CMA) is approximately 2–4.5% in infants and less than 0.5% in adults. Most children outgrow cow's milk allergy in early childhood, particularly that to the baked milk products. Immunotherapy with unheated cow's milk has been used as a treatment option for those who have not yet outgrown CMA, but the benefits must be balanced with the adverse effects.

**Objective:**

These evidence-based guidelines from the World Allergy Organization (WAO) intend to support patients, clinicians, and others in decisions about the use of oral and epicutaneous immunotherapy for the treatment of IgE-mediated CMA.

**Methods:**

WAO formed a multidisciplinary guideline panel balanced to include the views of all stakeholders and to minimize potential biases from competing interests. The McMaster University GRADE Centre supported the guideline-development process, including updating or performing systematic evidence reviews. The panel prioritized clinical questions and outcomes according to their importance for clinicians and patients. The Grading of Recommendations Assessment, Development and Evaluation (GRADE) approach was used, including GRADE Evidence-to-Decision frameworks, which were subject to public comment.

**Results:**

After a careful review of the summarized evidence and thorough discussions the WAO guideline panel suggests: a) using oral immunotherapy with unheated cow's milk in those individuals with confirmed IgE-mediated CMA who value the ability to consume controlled quantities of milk more than avoiding the large adverse effects of therapy, b) not using oral immunotherapy with unheated cow's milk in those who value avoiding large adverse effects of therapy more than the ability to consume controlled quantities of milk, c) using omalizumab in those starting oral immunotherapy with unheated cow's milk, d) not using oral immunotherapy with baked cow's milk in those who do not tolerate both unheated and baked milk, and e) not using epicutaneous immunotherapy outside of a research setting. The recommendations are labeled “conditional” due to the low certainty about the health effects based on the available evidence.

**Conclusions:**

Clinicians, patients, and their family members might want to discuss all the potential desirable and undesirable effects of oral immunotherapy for IgE-mediated CMA and integrate them with the patients' values and preferences before deciding on a treatment option. More robust research is needed to determine with greater certainty which interventions are likely to be the most beneficial with the least harms, and to develop safer, low-cost, and equitable treatments.

## Summary of recommendations (executive summary)

### Background

The prevalence of cow's milk allergy (CMA) is approximately 2–4.5% in infants and less than 0.5% in adults. Most children outgrow cow's milk allergy in early childhood, particularly that to the baked milk products. Immunotherapy with unheated cow's milk has been used as a treatment option for those who have not yet outgrown CMA, but the benefits must be balanced with the adverse effects.

### Methods

These World Allergy Organization (WAO) guidelines are based on updated and original systematic reviews of evidence conducted under the direction of the McMaster University GRADE Centre with international collaborators. The panel followed best practices for guideline development recommended by the Institute of Medicine and the Guidelines International Network (GIN).^1^, ^2^, ^3^ The panel used the Grading of Recommendations Assessment, Development and Evaluation (GRADE) approach to assess the certainty in the evidence and formulate recommendations.^4^^,^^5^

### Interpretation of strong and conditional recommendations

The strength of a recommendation is expressed as either strong (“the guideline panel recommends …”), or conditional (“the guideline panel suggests …”) and has the following interpretation:

#### Strong recommendation

• For patients: most fully informed people in this situation would want to follow the recommended course of action, and only a small proportion would not.
• For clinicians: most individuals should receive the intervention or test. Formal decision aids are not likely to be needed to help individual patients make decisions consistent with their values and preferences.
• For policy makers: the recommendation can be adopted as policy in most situations. Adherence to this recommendation according to the guideline could be used as a quality criterion or performance indicator.

#### Conditional recommendation

• For patients: the majority of fully informed people in this situation would want the suggested course of action, but many would not, and it may need more discussion between them and their healthcare professional first.
• For clinicians: recognize that different choices will be appropriate for individual patients and that you must help each patient arrive at a management decision consistent with his or her values and preferences. Decision aids may be useful in helping individuals to make decisions consistent with their values and preferences.
• For policy makers: policymaking will require substantial debate and involvement of various stakeholders. Performance measures about the suggested course of action should focus on documentation of an appropriate decision-making processes.

### Assumed values and preferences

The guideline panel rated anaphylaxis and other severe symptoms, use of intramuscular epinephrine, emergency department visits, and ability to consume cow's milk (prioritized outcomes) as critical for decision making and placed a high value on these outcomes and avoiding them with the interventions that were evaluated.

### Explanations and other considerations

These recommendations take into consideration cost, impact on health equity, acceptability by stakeholders, and feasibility of implementation (Table 1).

**Table 1:** Summary of the recommendations

Question 1: Should oral immunotherapy with unheated cow's milk, rather than no immunotherapy, be used in persons with IgE-mediated CMA?
**Recommendation 1A:**
We suggest oral immunotherapy with unheated cow's milk, rather than no immunotherapy, for those people with IgE-mediated CMA who place a higher value on being able to consume milk (even if in small amounts) with less need to follow a strict avoidance diet, and a lower value on allergic reactions during oral immunotherapy.
(CONDITIONAL recommendation based on a moderate certainty evidence about health effects)
**Recommendation 1B:**
We suggest that clinicians do not use oral immunotherapy with cow's milk in those people with IgE-mediated CMA who place a higher value on avoiding allergic reactions during oral immunotherapy, and a lower value on being able to consume cow's milk (even if in small amounts) with less need to follow a strict avoidance diet.
(CONDITIONAL recommendation based on a moderate certainty evidence about health effects)
**Question 2:** Should **omalizumab**, rather than no anti-IgE therapy, be used during oral immunotherapy with unheated cow's milk in persons with IgE-mediated CMA?
**Recommendation 2:**
We suggest that clinicians use omalizumab, compared with not using it, during the initial stages of oral immunotherapy with unheated cow's milk in people with IgE-mediated CMA.
(CONDITIONAL recommendation based on a very low certainty evidence about health effects)
**Question 3:** Should **oral immunotherapy with baked cow's milk,** rather than no immunotherapy, be used for persons with IgE-mediated CMA who do not tolerate baked cow's milk?
**Recommendation 3:**
In people with IgE-mediated CMA who do not tolerate unheated and baked milk, we suggest that clinicians do not use oral immunotherapy with baked cow's milk.
(CONDITIONAL recommendation based on a very low certainty evidence about health effects)
Remark: This recommendation concerns persons who react to very small doses of baked milk. Persons with IgE-mediated CMA who do tolerate certain amounts of baked cow's milk can continue consuming it and advance with the amounts tolerated under physician supervision.
**Question 4:** Should **epicutaneous immunotherapy with cow's milk,** rather than no immunotherapy, be used for persons with IgE-mediated CMA?
**Recommendation 4:**
More rigorously designed and performed studies of epicutaneous immunotherapy for IgE-mediated CMA are needed to make a recommendation for clinical practice. Thus, we recommend that, for now, clinicians do not use epicutaneous immunotherapy for IgE-mediated CMA outside of the research setting.
(STRONG recommendation based on a very low certainty evidence about health effects)

## Introduction

### Aim of these guidelines and their specific objectives

The purpose of this document is to evaluate the current evidence and provide guidance on the use of immunotherapy in the treatment of IgE-mediated CMA. These guidelines are intended to be international in scope. The primary target audience of these guidelines are specialists in allergy, children and adults with IgE-CMA, and caregivers of children with IgE-CMA. Pediatricians, general practitioners, and allied health practitioners may also benefit from these guidelines. This document may also serve as the basis for development and implementation of locally adapted guidelines. By identifying gaps in the research literature, these guidelines may help researchers to direct attention to topics on which more studies are needed.

This is the first of 3 documents presenting the recommendations of the World Allergy Organization (WAO) Diagnosis and Rationale for Action against Cow's Milk Allergy (DRACMA) guidelines updated in 2021/2022. In this document we present the recommendations about the use of immunotherapy for the management of IgE-mediated cow's milk allergy (CMA). These recommendations replace the original DRACMA guidelines published in 2010.^6^

### Description of the health problem

IgE-mediated food allergy affects 1–5% of children (with a prevalence of self-reported food allergy of 9% [range: 2.5%–38%]).^7^, ^8^, ^9^ CMA is one of the most common causes of food allergy among children.^10^ Self-reported prevalence of CMA is 1.2%–17% and the prevalence of IgE sensitization ranges from 2% to 9% with approximately 0.6% (95% CI: 0.3–1.0) being sensitized and symptomatic.^7^ Clinical manifestations of IgE-mediated cow's milk allergy (IgE-CMA) include rapid-onset urticaria and pruritus, angioedema, rhinoconjunctivitis, difficulty breathing, gastrointestinal symptoms, and anaphylaxis. IgE-mediated reactions are rapid in onset, typically beginning within minutes to 2 h from the time of ingestion. Anaphylactic reactions to cow's milk can be severe^11^ and fatal cases have been described.^12^^,^^13^ Cow's milk accounts for 21% of anaphylaxis fatalities in children.^14^

Patients usually outgrow IgE-CMA during childhood or adolescence, with the majority achieving tolerance around the age of 3–5 years. Studies investigating the natural history of achieving tolerance to cow's milk in children with IgE-CMA suggest that 12–37% of children outgrow IgE-CMA before reaching school age.^15^, ^16^, ^17^

The mechanisms by which IgE-CMA resolves are not well understood and involve not only specific IgE but also non-IgE and non-antibody mediated immune processes.^18^ In most patients with IgE-CMA milk-specific IgE levels tend to decrease over time and are the best-known predictor of achieving the tolerance.^18^ However, loss of milk-specific IgE is not a requirement for the resolution of IgE-CMA, since some patients become clinically tolerant even with persistently elevated IgE levels.^19^ In general, the higher the level of cow's milk-specific IgE in the serum at the time of diagnosis, the longer is the duration of disease^19^ and the less likely the child is to become tolerant over time.^15^

Strict elimination of cow's milk proteins from the diet can be difficult. Hence, accidental ingestions of cow's milk are frequent, especially in processed foods, posing a high risk of severe allergic reactions in a proportion of milk allergic subjects.^20^^,^^21^

### Description of the interventions

Some studies have investigated oral immunotherapy (OIT) with unheated (ie, not baked) as a treatment for IgE-CMA.^22^ The approach generally follows the same principles as immunotherapy for other food allergic disorders.

Although no uniform protocol has been developed, overall, unheated cow's milk OIT of IgE-CMA involves administering (usually daily) small doses of cow's milk increased incrementally (usually biweekly or weekly) during a build-up phase until a target maintenance dose is reached or the patient reaches dose-limiting symptoms.^23^ The duration of the build-up phase is variable (usually 3–9 months) depending on the protocol design and an individual's ability to continue with the regimen despite adverse effects and a strict dosing schedule. The dose is typically taken at the same time every day, often with a meal to reduce the risk of gastrointestinal adverse effects. Due to the risk of triggering a more severe reaction the daily dose cannot be taken when the patient is tired or sick, on an empty stomach, if menstruating, within 2 hours of hot shower or bath, within 2–4 hours of physical exertion (eg, sports), and within 1–2 hours of sleeping or waking up. Additional risks for reactions to the treatment dose can occur if the patient is stressed, if it is during the pollen season, or if non-steroidal anti-inflammatory drug is also taken.^24^ Dose escalations during the build-up phase are typically conducted under physician's supervision because of the risk of allergic reactions. Keeping a strict schedule can also complicate the ability to travel or participate in prolonged activities away from home.

The build-up phase is followed by a maintenance phase with regular, daily intake of a maximum tolerated amount of cow's milk (eg, 250 ml of unheated cow's milk daily). The optimal duration of maintenance is undefined and is currently commonly assumed to be indefinite. Patients may experience unexpected breakthrough reactions to the maintenance dose, despite having tolerated that dose for weeks or even months prior. Potential risk factors for breakthrough reactions are the same as those for the adverse reactions during the build-up phase mentioned above.

In a systematic review supporting these guidelines^82^ we found that cow's milk OIT may be associated with an increased risk of discontinuing treatment due to adverse effects, but the estimates were imprecise (risk ratio: 1.92, 95% CI: 0.92 to 3.99; risk difference: 6 more per 100 patients, 95% CI: from 0 to 18 more). The mechanisms involved in OIT of IgE-CMA are also not well understood. Altogether, there are significant potential benefits and harms of currently available forms of OIT to unheated milk.

We found no research evidence about the estimated direct and indirect costs of OIT with milk. Based on the experience of panel members, total cost of OIT is likely to be large, because it requires the availability of trained health care professionals, appropriate clinical facilities to provide OIT and deal with adverse effects, and the availability of emergency physician to provide advice during maintenance OIT at home.

**Figure:**
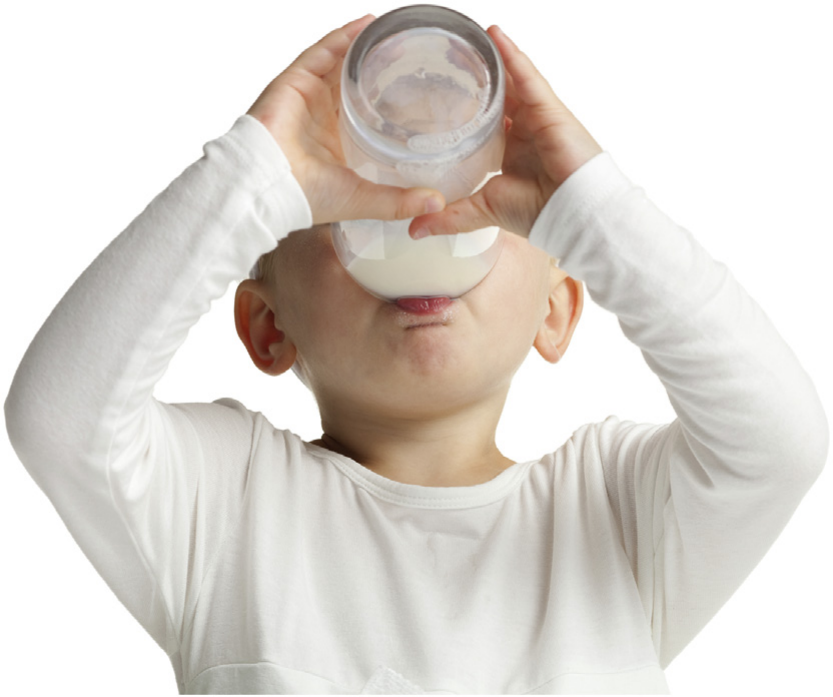

## Methods

### Organization, panel composition, planning, and coordination

These guidelines were developed by: (a) an international panel of key stakeholders consisting of 30 members, including patients with cow's milk allergy, dieticians, primary care professionals, and specialists in pediatrics, allergy, and gastroenterology; and (b) a methodology team consisting of 8 researchers with experience in evidence synthesis and guideline development.

The guideline panel developed and graded the recommendations and assessed the certainty in the supporting evidence following the GRADE approach.^4^^,^^5^ The overall guideline-development process, including funding of the work, panel formation, management of conflicts of interest, internal and external review, and organizational approval, was guided by WAO policies and procedures derived from the GIN-McMaster Guideline Development Checklist (http://cebgrade.mcmaster.ca/guidecheck.html)^25^ and intended to meet recommendations for trustworthy guidelines by the Institute of Medicine and the Guidelines International Network.^1^, ^2^, ^3^^,^^26^

Project oversight was provided by the coordination panel chairpersons: AF (pediatrician and content expert) and HJS (internist and expert in guideline-development methodology).

WAO vetted and appointed individuals to the guideline panel. The McMaster GRADE Centre vetted and retained researchers to conduct systematic reviews of evidence and coordinate the guideline-development process, including use of the GRADE approach. The membership of the panel and the evidence synthesis team is described in Online Supplement.

In addition to synthesizing evidence systematically, the evidence synthesis team supported the guideline-development process, including determining methods, preparing agendas, and meeting materials, and facilitating panel discussions. The panel's work was done using Web-based tools: SurveyMonkey (www.surveymonkey.com), Google Forms (docs.google.com/forms/) and GRADEpro Guideline Development Tool (www.gradepro.org),^27^ 1 face-to-face meeting, and subsequent online meetings.

### Guideline funding and the management of competing interests

Development of these guidelines was funded by WAO and the McMaster University GRADE Centre. WAO staff supported panel appointments and coordinated meetings but had no role in choosing the guideline questions or determining the recommendations. Members of the guideline panel received travel reimbursement for attendance of an in-person meeting; they received no other payments. Funding from WAO was also used to partially remunerate for time spent by research assistants and students conducting the systematic reviews, and on information technology. Some researchers who contributed to the systematic evidence reviews participated without remuneration to fulfill requirements of an academic degree or program.

Conflicts of interest of all participants were managed according to WAO policies (Online Supplement 2) based on recommendations of the Institute of Medicine (now National Academy of Medicine)^28^ and the Guidelines International Network.^3^ At the time of appointment, the majority of the guideline panel, including the guideline panel co-chair (HJS), had no conflicts of interest as defined by WAO and judged by an independent and anonymous WAO committee (ie, no current material interest in any commercial entity with a product that could be affected by the guidelines). One DRACMA guideline panel co-chair (AF) had the knowledge about the commercial entities that provide WAO with educational grants and as such he abstained from voting on all recommendations in the DRACMA guidelines.

Before the appointment to the panel and the commencement of work, all research team members who prepared systematic reviews and all DRACMA panel members completed the World Health Organization (WHO) declaration of interest forms. An external, anonymous WAO committee reviewed each disclosure form. Research team members who were deemed to have a real, potential, or perceived conflict of interest related to the topic of a systematic review were excused from taking part in that review. Guideline panel members who were deemed to have a real, potential, or perceived conflict of interest that was manageable abstained from voting on recommendations related to that interest. The Evidence-to-Decision (EtD) tables for each recommendation list individuals who were excused from making judgments about that recommendation. One proposed panel member was deemed to have disqualifying competing interests and was excused from participation in the entire DRACMA project.

### Selection of questions and outcomes of interest

Building on the original WAO DRACMA guidelines from 2010,^6^ members of the guideline panel and methodology team collaboratively brainstormed potential questions to be addressed in these guidelines. Using group discussion and online polling software (www.surveymonkey.com), we ranked the questions in terms of priority. The selected interventions and questions represent the top-prioritized issues identified by the group:

1. Should OIT with unheated milk vs. strict avoidance of milk be used for IgE-mediated CMA?
2. Should OIT with baked milk vs. strict avoidance of milk be used for IgE-mediated CMA?
3. Should OIT with baked milk vs. OIT with unheated milk be used for IgE-mediated CMA?
4. Should omalizumab + OIT with unheated milk vs. OIT with unheated milk alone be used for IgE-mediated CMA?
5. Should an epicutaneous IT with unheated milk vs. OIT with unheated milk be used for IgE-mediated CMA?

The panel selected outcomes of interest for each question *a priori*, following the approach described in detail elsewhere.^29^ In brief, the panel brainstormed all possible outcomes before rating their relative importance for decision making following the GRADE approach. Rating outcomes by their relative importance can help focus attention on those outcomes that are considered most important and help to resolve or clarify potential disagreements. The list of outcomes rated highly by the panel and those identified as important based on the literature reviews was further refined.

For most questions, we deemed the following outcomes to be critically important: anaphylaxis to cow's milk, need for intramuscular epinephrine (adrenaline), severe gastrointestinal or respiratory symptoms, generalized erythema or urticaria, ability to consume cow's milk and milk products without a reaction, ability to accidentally consume a small amount of cow's milk without a reaction, adverse effects leading to the discontinuation of treatment, and emergency department visit. We also deemed the following outcomes to be important but less critically so: non-severe angioedema, mild and/or local allergic symptoms, hospital admission, development of eosinophilic esophagitis (EoE), and quality of life among people with IgE-CMA and their caregivers.

### Evidence review and development of recommendations

For each guideline question, the evidence synthesis team prepared an evidence profile^30^^,^^31^ and a GRADE evidence-to-decision (EtD) table^32^^,^^33^ using the GRADEpro software. Each EtD table summarizes the results of a systematic review of the literature that was either updated or performed de novo for these guidelines. The EtD table includes the information about the effects of interventions on health outcomes, the values and preferences (ie, relative importance of outcomes), resource utilization (cost-effectiveness), health equity issues, acceptability of interventions to stakeholders, and the feasibility of implementation. The guideline panel reviewed the draft EtD tables before and during the guideline meetings making suggestions for corrections and clarifications. To ensure that recent studies were not missed, searches were updated in June 2021, and panel members were asked to suggest any additional studies that they may have known about and that fulfilled the inclusion criteria for the systematic reviews.

Under the direction of the McMaster GRADE Centre the evidence synthesis team followed the general methods outlined in the Cochrane Handbook for Systematic Reviews of Interventions^34^ for conducting updated or new systematic reviews of intervention effects. When existing reviews were used, judgments of the original authors about risk of bias were either randomly checked for accuracy and accepted or conducted *de novo* if they were not available or not reproducible. For new reviews, risk of bias was assessed at the health outcome level using the Cochrane Collaboration's risk of bias tool for randomized trials^35^ and the Newcastle-Ottawa scale for single-arm nonrandomized studies.^36^ Subsequently, the certainty in the body of evidence about the health effects (also known as quality of the evidence or confidence in the estimated effects) was assessed for each outcome of interest following the GRADE approach based on the following domains: risk of bias, precision, consistency and magnitude of the estimates of effects, directness of the evidence, risk of publication bias, presence of large effects, dose–effect relationship, and an assessment of the effect of plausible residual and opposing confounding.^37^, ^38^, ^39^, ^40^, ^41^, ^42^ The certainty was categorized into 4 levels ranging from very low to high.^5^^,^^43^

During a 2-day in-person meeting, followed by online communication and conference calls, the panel developed recommendations based on the evidence summarized in the EtD tables. For each recommendation, the panel took a population perspective and came to consensus on the following: the certainty in the evidence, the balance of benefits and harms of the compared management options, and the assumptions about the values and preferences associated with the decision. The guideline panel also explicitly took into account the extent of resource use associated with alternative management options. The panel agreed on the recommendations (including direction and strength), remarks, and qualifications by consensus or, in rare instances, by voting (an 80% majority was required for a strong recommendation), based on the balance of all desirable and undesirable consequences. All members of the panel reviewed and approved the final guidelines.

### Interpretation of strong and conditional recommendations

The recommendations are labeled as “strong” or “conditional” according to the GRADE approach. The words “the guideline panel recommends” are used for strong recommendations, and “the guideline panel suggests” for conditional recommendations. Table 2 provides GRADE's interpretation of strong and conditional recommendations by patients, clinicians, health care policy makers, and researchers.

**Table 2:** Interpretation of strong and conditional recommendations

Implications for:	Strong recommendation	Conditional recommendation
Patients	Most fully informed people in this situation would want the recommended course of action, and only a small proportion would not.	The majority of fully informed people in this situation would want the suggested course of action, but many would not, and it may need more discussion between them and their healthcare professional first.
Clinicians	Most individuals should follow the recommended course of action. Formal decision aids are not likely to be needed to help individual patients make decisions consistent with their values and preferences.	Different choices will be appropriate for individual patients; clinicians must help each patient arrive at a management decision consistent with his or her values and preferences. Decision aids may be useful in helping individuals to make decisions consistent with their individual risks, values, and preferences.
Policy makers	The recommendation can be adopted as policy in most situations. Adherence to this recommendation according to the guideline could be used as a quality criterion or performance indicator.	Policymaking will require substantial debate and involvement of various stakeholders. Performance measures should assess if decision-making is appropriate.
Researchers	The recommendation is supported by credible research or other convincing judgments that make additional research unlikely to alter the recommendation. On occasion, a strong recommendation is based on low or very low certainty of the evidence; in such instances, further research may provide important information that may alter that recommendation.	The recommendation is likely to be strengthened (for future updates or adaptation) by additional research. An evaluation of the conditions and criteria (and the related judgments, research evidence, and additional considerations) that determined the conditional (rather than strong) recommendation will help identify possible research gaps.

### Document review

Draft guidelines were reviewed by all panel members and revised where necessary. The guidelines were then subjected to peer review by the World Allergy Organization Journal. All reviewers' comments were addressed, but in both above instances no changes were made to the recommendations.

### How to use these guidelines

#### Terminology

In this document we use the term “unheated cow's milk” referring to fresh cow's milk, milk shakes, and dairy products like cream, yogurt, ice cream, cheese, and others that have not been baked. In contrast, by “baked cow's milk” we refer to cow's milk that has been exposed to high heat for an extended period of time within a matrix such as wheat (eg, biscuits and cakes, muffins, scones, croissants, waffles, shortbread, breadcrumb-covered meats, and other baked recipes that contain milk, cream, cheese, or butter). We use the word “people” whenever we mean both children and adults.

#### Intended use

WAO guidelines are primarily intended to help clinicians make decisions about diagnostic and treatment alternatives. They may also be used by patients. Other purposes are to inform policy, education, and advocacy, and to state future research needs.

These guidelines are not intended to serve or to be seen as a standard of care. Decision makers should not treat the recommendations in these guidelines as binding mandates. No recommendation can take into account all of the variable circumstances that might affect the potential benefits, harms, and burdens of an intervention in individual patients or in a given clinical setting. Clinicians must make decisions based on the clinical presentation of each individual patient, ideally through a shared process that considers the patient's values and preferences with respect to the anticipated outcomes of the chosen management option. Clinicians' and patients' decisions may also be constrained by the realities of a specific clinical setting and local resources, including, but not limited to, institutional policies, time limitations, and availability of treatments. Thus, no one charged with overseeing or evaluating the actions of clinicians should apply the recommendations by rote or in a blanket fashion.

These guidelines may not include all appropriate methods of care for the clinical scenarios described. As science advances and new evidence becomes available, recommendations may become outdated. Following these guidelines cannot guarantee successful outcomes. WAO does not warrant or guarantee any products described in these guidelines.

#### Translation and quoting

When quoting or translating recommendations from these guidelines, any qualifying remarks that accompany each recommendation should not be omitted (including statements regarding special circumstances and assumed values and preferences). These statements are integral to the recommendations and serve to facilitate more accurate interpretation.

## Summary of findings and recommendations

### Question 1: Should oral immunotherapy with unheated cow's milk, rather than no immunotherapy, be used in persons with IgE-mediated CMA?

#### Summary of the evidence, benefits, and harms

Summary of findings and the evidence-to-decision tables for this question (see Online Supplement) present details of the systematic review of the effects of OIT with unheated cow's milk on the outcomes of interest. For this question we updated the systematic review that we performed for the previous DRACMA guidelines^22^ and we will publish it separately.

Panel members thought that the ability to accidentally consume even a small amount of cow's milk (eg, 5 ml) or milk products without a reaction is the main benefit of OIT, whereas anaphylaxis and other severe reactions to OIT are the main adverse effects. Based on our systematic review, using unheated cow's milk OIT increased the probability of being able to consume cow's milk and milk products as assessed with a negative supervised graded food challenge with ≥150 ml of unheated cow's milk (RR: 12, 95% CI: 6 to 26; risk difference: 28 more per 100, 95% CI: 11 to 63) and the probability of being able to accidentally ingest even a small amount of cow's milk without a reaction as assessed with a negative supervised graded food challenge with ≥5 ml of unheated cow's milk (RR: 10, 95% CI: 5 to 21; risk difference: 35 more per 100, 95% CI: 15 to 74). At the same time cow's milk OIT increased the risk of anaphylaxis (rate ratio: 60, 95% CI: 15 to 244; rate difference: 541 more anaphylactic reactions per 100 persons per year, 95% CI: 450 to 632), need for epinephrine use (rate ratio: 29, 95% CI: 7 to 117; rate difference: 268 more epinephrine injections per 100 persons per year, 95% CI: 203 to 333), and severe gastrointestinal (RR: 6.9, 95% CI: 1.6 to 30.9; risk difference: 28 more per 100, 95% CI: 3 to 100) and respiratory adverse effects (RR: 49, 95% CI: 3 to 771; risk difference: 77 more per 100, 95% CI: 62 to 92).

#### Assumed values and preferences

Panel members agreed that people with IgE-CMA place high value on avoiding severe allergic reactions. These can occur as a result of accidental ingestion of milk or as a result of cow's milk OIT. However, there might be important variability in how they value other outcomes. For example, some school-aged patients may place more (or less) value on the ability to consume milk and milk products relative to the ability to take part in social activities (eg, OIT with milk might preclude the ability to take part in school trips or camps). Older patients are likely to vary in their perception of burden related to unheated cow's milk OIT (eg, having to follow the regular daily dosing or avoid exercise after taking a daily dose of OIT).

For detailed consideration of values and preferences, acceptability of interventions, feasibility of implementation, and required resources please see the Evidence-to-Decision table in the Online Supplement.

#### Balance between desirable and undesirable health effects

Panel members thought that the overall balance of effects does not favor either intervention (i.e., unheated cow's milk OIT vs avoidance diet only). However, they acknowledged that it mostly depends on values and preferences of patients and/or their caregivers for individual outcomes. For those who value the ability to consume milk more than adverse effects during OIT, the balance may favor OIT. For those who place more value on avoiding allergic reactions, the balance may favor staying on an elimination diet without OIT.

#### Recommendations

##### Recommendation 1A

We suggest oral immunotherapy with unheated cow's milk, rather than no immunotherapy, for those people with IgE-mediated CMA who place a higher value on being able to consume milk (even small amounts) with less need to follow a strict avoidance diet, and a lower value on allergic reactions during OIT.

(CONDITIONAL recommendation based on moderate certainty evidence about health effects)

##### Recommendation 1B

We suggest that clinicians do not use oral immunotherapy with unheated cow's milk in those people with IgE-mediated CMA who place a higher value on avoiding allergic reactions during OIT, and a lower value on being able to consume cow's milk (even small amounts) with less need to follow a strict avoidance diet.

(CONDITIONAL recommendation based on moderate certainty evidence about health effects)

#### Special circumstances

• Adolescents and adults with persistent reactions who are unlikely to outgrow IgE-CMA may benefit from unheated cow's milk OIT more than those who are younger and still likely to outgrow it.
• Most studies were done on children – based on the indirect evidence extrapolated from children, the effects may be similar in adults, but this is less certain. Some evidence from immunotherapy studies with other foods suggests that adverse effects may be more frequent in adults than in children,^44^, ^45^, ^46^ tipping the balance of desirable and undesirable effects against the OIT in adults.

#### Implementation considerations

In all cases the diagnosis of IgE-CMA must be confirmed before commencing milk OIT. Patients and their families require specific education and training in the correct administration of OIT, prevention and treatment of adverse effects, and a priori defined rules for stopping cow's milk OIT. When choosing to perform OIT, clinicians may want to consider the situations listed in Table 3 which may be contraindications for starting and for continuation of OIT. Also, when choosing to perform OIT, clinicians need to monitor symptoms and adherence in all patients. Furthermore, clinicians should monitor growth and nutritional intake in small children before and periodically during the OIT procedure, because of the risk of nutritional deficiencies in subjects with dietetic restrictions (eg, caloric, calcium, and vitamin D deficiencies).

**Table 3:** Situations which may be contraindications for starting and/or continuation of OIT

- a patient and/or the family are not able to follow the OIT protocol for any reason (eg, scheduling conflicts, patient's athletic activities)
- a patient and/or their family have no access to epinephrine and/or are not able to properly use it when needed
- a patient has a confirmed history of previous frequent severe reactions
- a patient had multiple severe reactions to cow's milk OIT
- a patient has persistent gastrointestinal symptoms
- a patient has a concomitant asthma that is not well controlled
- a physician suggesting to use OIT is not able to devote sufficient time and resources to properly administering and monitoring OIT – this may require a 24 h per day, 7 days per week on-call service
- a preschool or school personnel does not accept providing and/or supervising milk OIT during school trips which might require the child to forgo school social activities or temporarily suspend the OIT

#### Implications for further research

When reviewing the evidence and considering all other factors influencing this recommendation, the guideline panel identified the following priorities for further research:

1. Patient adherence to long-term OIT, patients' needs in terms of suitable foods (variety) for long term intake, and the impact of OIT and long-term treatment on nutritional status
2. Properly designed and executed experimental studies (RCTs) in patients with moderate and severe IgE-CMA (including those with previous severe anaphylaxis) that would measure and report all important outcomes, including quality of life, and that would investigate: a) sustainability of the long-term beneficial effects, b) short-term and long-term adverse effects, c) relative effects of different doses (especially the starting dose) and different protocols of OIT to identify the best balance between desirable and undesirable effects of OIT, d) the effects of OIT with unheated milk compared with OIT with baked milk and with baked milk diet.
3. Studies to provide more information about the predictors of response to OIT and the resources required to offer OIT and its cost-effectiveness.
4. Qualitative studies of patients' and their families' knowledge of IgE-CMA and OIT, understanding the benefits and risks, and their expectations of the management of milk allergy (values and preferences).

**Figure:**
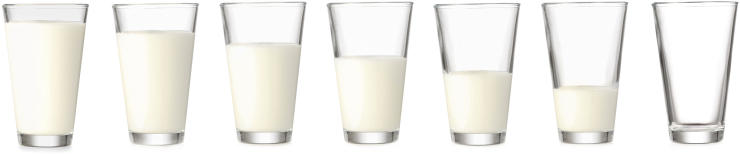

### Question 2: Should an anti-IgE antibody (omalizumab) be used, rather than not used, during oral immunotherapy with unheated cow's milk in people with IgE-mediated CMA?

#### Summary of the evidence, benefits, and harms

Summary of findings table (see Online Supplement) presents details of our systematic review of the effects of adding omalizumab to OIT on the outcomes of interest. We found only 1 RCT that investigated the use of omalizumab in OIT for CMA^47^ and 3 RCTs that investigated the use of omalizumab during the OIT with peanuts^48^ and multiple foods.^49^^,^^50^ In addition, we found no non-randomized studies (NRS) with a control group investigating omalizumab in OIT with cow's milk but we found 2 NRS of omalizumab in OIT with other foods.^51^^,^^52^ We also found 11 series of cases describing the use of omalizumab in OIT with cow's milk^53^, ^54^, ^55^, ^56^, ^57^, ^58^, ^59^, ^60^, ^61^, ^62^, ^63^ and 10 series of cases that used omalizumab for OIT with other foods.^64^, ^65^, ^66^, ^67^, ^68^, ^69^, ^70^, ^71^, ^72^ We used the results of studies of omalizumab during OIT with other foods as indirect evidence of the effects of omalizumab on adverse effects of unheated cow's milk OIT.

We found very low certainty evidence that adding omalizumab in advance of and then during the initial phase of OIT with unheated milk reduces the risk of severe adverse effects. Omalizumab may have reduced the risk of anaphylaxis (RR: RR 0.34, 95% CI: 0.11 to 1.05; risk difference: 7 fewer per 100, 95% CI: from 9 fewer to 1 more) and epinephrine use (RR 0.23, 95% CI: 0.05 to 0.97; risk difference: 19 fewer per 100, 95% CI: from 24 fewer to 1 fewer), and had little effect on other symptoms, but the evidence is very uncertain (see the summary of findings table in the Online Supplement). Most panel members thought that the desirable health effects of adding omalizumab to unheated cow's milk OIT were moderate and the undesirable were small or trivial.

#### Assumed values and preferences

Panel members agreed that the values and preferences for OIT without omalizumab (see Assumed values and preferences) would apply similarly in this context. Panel members were divided on whether the addition of omalizumab to OIT with cow's milk would be acceptable to stakeholders. The main barrier to acceptability was the cost of anti-IgE therapy.

Clinicians, patients, and their family members may vary in their perception of risk and the relative value they place on avoiding reactions with accidental exposure to cow's milk or with OIT. Thus, some clinicians, patients, and family members may see value in adding omalizumab to OIT while others will not.

#### Balance between desirable and undesirable health effects

Majority of panel members thought that the balance of health effects favors adding omalizumab to OIT with cow's milk. However, they acknowledged that this judgment is based on very low certainty evidence.

#### Recommendation 2

We suggest that clinicians use omalizumab, compared with not using it, during the initiation of oral immunotherapy with unheated cow's milk in people with IgE-mediated CMA.

(CONDITIONAL recommendation based on very low certainty evidence about health effects)

#### Implementation considerations

All implementation considerations for OIT (see Implementation considerations) also apply to OIT with omalizumab. In addition, dosing of anti-IgE needs to be based on serum total IgE measurement and body mass/weight.

Panel members agreed that the monitoring of the OIT with anti-IgE should be the same as without it, however, clinicians should also monitor symptoms after anti-IgE injection.

Panel members found the following to be currently the main barriers to implementation of omalizumab (in addition to the barriers for implementation of OIT itself):

- additional cost to already expensive OIT
- limited access to omalizumab in many countries (in some jurisdictions patients with coexisting severe asthma and/or chronic spontaneous urticaria may be more likely to have access to omalizumab).

Currently in all jurisdictions using omalizumab for OIT would be off label.

#### Implications for further research

The panel identified a need for high-quality research regarding the following topics:

1. Dosing of omalizumab and duration of treatment with omalizumab in the context of food OIT.
2. Identification of patients who would benefit the most from using omalizumab in this context.
3. Well designed and executed RCTs measuring important desirable and undesirable health effects and quality of life.

**Figure:**
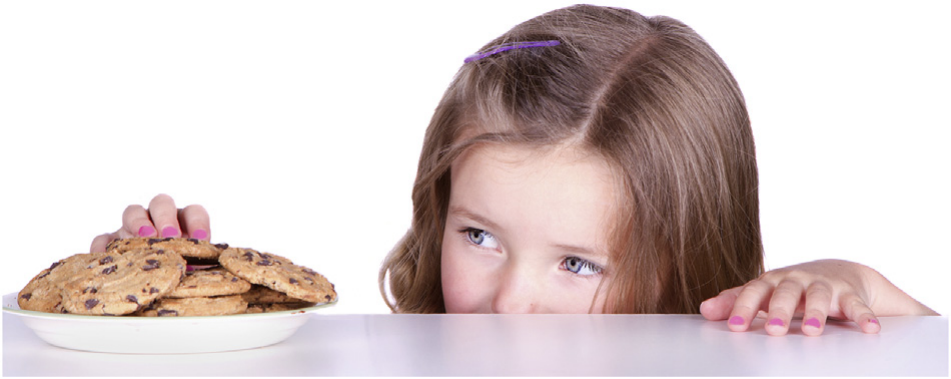

### Question 3: Should oral immunotherapy with baked cow's milk, rather than no immunotherapy, be used for persons with IgE-mediated CMA who do not tolerate unheated and baked cow's milk?

#### Summary of evidence, benefits, and harms

Summary of findings table (see Online Supplement) presents details of our systematic review of the effects of OIT with baked milk on the outcomes of interest in people with IgE-CMA who do not tolerate baked milk. We defined OIT with baked milk as a specific protocol similar to OIT with unheated milk rather than the inclusion of small amounts of baked milk via a “milk ladder”. We found very low certainty evidence from 2 series of cases that OIT with baked milk may increase the risk of adverse effects with possibly increased ability to consume baked milk assessed with either passing a supervised graded food challenge with 254 ml of unheated cow's milk or ability to eat 1.3 g/d of baked milk protein.^73^^,^^74^ Panel members judged the beneficial and undesirable effects to be small to moderate. They noted that the ability to tolerate baked milk would allow patients to substantially expand their diet. Panel members also noted that lack of controls does not allow an estimate of what proportion of those who were able to eat baked milk after OIT gained it owing to OIT or naturally outgrowing milk allergy.

#### Assumed values and preferences

Panel members agreed that values and preferences that people place on the outcomes of OIT with baked milk would be the same as for OIT with unheated milk (see Assumed values and preferences)

#### Balance between desirable and undesirable health effects

Panel members noted that the certainty of the evidence is very low, and the conclusions are difficult to draw. However, they thought that the desirable and undesirable effects of OIT with baked milk were closely balanced. Panel members agreed that more and higher quality evidence would be desirable to obtain and once available, it is likely to influence the strength and the direction of this recommendation.

Panel members noted that the balance will ultimately depend on patient's and their family's values and preferences.

#### Recommendation 3

In people with IgE-mediated CMA who do not tolerate baked milk, we suggest that clinicians do not use oral immunotherapy with baked cow's milk.

(CONDITIONAL recommendation based on very low certainty evidence about health effects)

*Remark*: This recommendation concerns persons who react to very small doses of baked milk. Persons with IgE-CMA who do tolerate certain amounts of baked cow's milk can continue consuming it and advance with the amounts tolerated under physician supervision.

#### Implementation considerations

When choosing to perform OIT with baked milk, clinicians need to consider all implementation issues similar to the OIT with unheated cow's milk (see Implementation considerations)

#### Implications for further research

The panel identified a need for high-quality research regarding the following topics:

1. Temperature and time of heating/baking cow's milk products.
2. The effects of OIT with baked milk on quality of life of patients and their family members.
3. Studies to establish the standardized protocol (starting dose, escalation schedule, and maintenance dose), safety and efficacy of baked milk OIT
4. Studies investigating whether baked milk OIT could be utilized as transition to unheated milk OIT.
5. Costs of OIT with baked milk.

**Figure:**
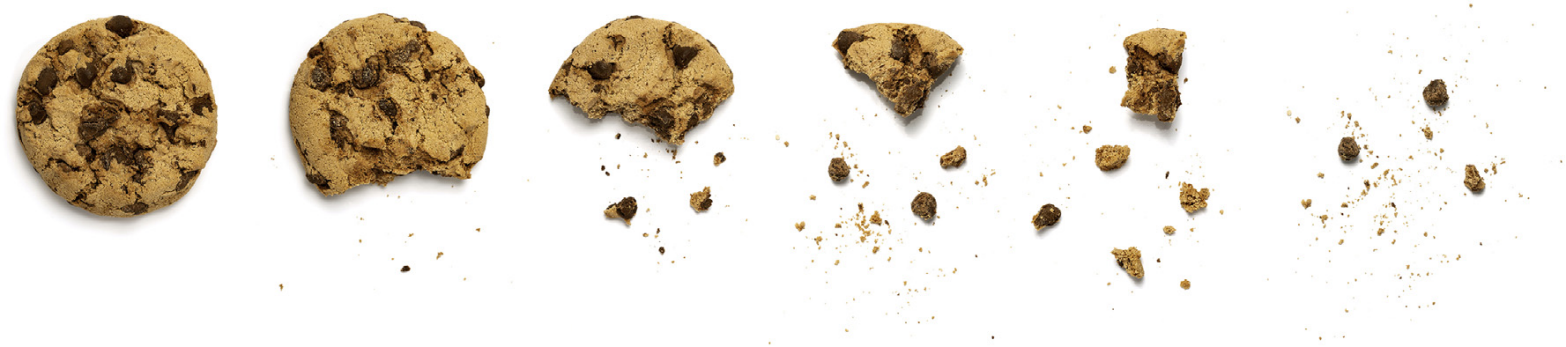

### Question 4: Should epicutaneous immunotherapy with cow's milk, rather than no immunotherapy, be used for people with IgE-mediated CMA?

#### Summary of the evidence

We performed a separate systematic review of studies that used epicutaneous immunotherapy (EPIT) for IgE-CMA. We found 6 records that referred to the same 2 studies.^75^^,^^76^ We present the detailed results in summary of findings table in the Online Supplement. Both studies were small and there were no events noted for most of the outcomes of interest. We identified 1 more randomized trial of EPIT for milk-induced eosinophilic esophagitis (EoE), but its results have not yet been published.^77^

#### Conclusions and implications for further research

Panel members thought that the overall balance of effects cannot be assessed before more evidence becomes available and they suggested that it might be prudent to perform more well designed and executed studies investigating the effects of EPIT for IgE-CMA. Panel members also noted that EPIT is currently not available outside some experimental settings and opted to recommend additional research.

#### Recommendation 4

More rigorously designed and performed studies of epicutaneous immunotherapy for IgE-mediated CMA are needed to make a recommendation for clinical practice. Thus, we recommend that, for now, clinicians do not use epicutaneous immunotherapy for IgE-mediated CMA outside of the research setting.

(STRONG recommendation based on very low certainty evidence about health effects)

## Strengths and limitations of these guidelines

The recommendations in these guidelines may help support informed decision-making by clinicians, as well as individuals with IgE-CMA and their caregivers. The strength of these guidelines is in the diverse, international guideline panel including clinicians treating IgE-CMA, researchers, and patients with IgE-CMA themselves, who performed systematic reviews of available evidence and followed the systematic GRADE approach to develop recommendations. However, the evidence that informs these guidelines has important limitations. Recommendations about the use of omalizumab in OIT, OIT with baked milk, and EPIT are based on very low certainty evidence. Recommendation about the use of OIT with unheated milk recognizes that the best choice for individual patients will depend on their values and preferences for certain outcomes (ie, ability to consume milk vs higher risk of severe allergic reactions during OIT), however, no study investigated quality of life of either the patients themselves or their caregivers. More research may also be beneficial for assessing the cost-effectiveness and feasibility of implementation of OIT in practice. The findings of future research could substantially affect the recommendations in these guidelines and/or make them more specific.

## What others are saying and what is new in these WAO guidelines

The Joint Task Force on Practice Parameters, representing the American Academy of Allergy, Asthma & Immunology (AAAAI), the American College of Allergy, Asthma & Immunology (ACAAI), and the Joint Council of Allergy, Asthma & Immunology (JCAAI) most recent guidelines for the management of food allergy were published in 2014.^78^ The guidelines state that “although immunotherapeutic approaches, such as oral immunotherapy (OIT), in clinical trials show promise in treating food allergy, they are not ready for implementation in clinical practice at the present time because of inadequate evidence for therapeutic benefit over risks of therapy”. These guidelines do not mention the use of omalizumab during OIT, the OIT with baked milk, or the epicutaneous immunotherapy.

The Canadian Society of Allergy and Clinical Immunology (CSACI) guidelines published in 2020 state that OIT with food allergens in general, including cow's milk, is “recommended as a treatment to achieve desensitization”.^79^ They add that “short-term concomitant use of omalizumab can be considered in challenging cases”. The guidelines do not comment on the use of OIT with baked milk and on the epicutaneous immunotherapy.

The European Academy of Allergy and Clinical Immunology (EAACI) Guidelines from 2018 on allergen immunotherapy of the IgE-mediated food allergy state that “OIT is recommended as a treatment option to increase threshold of reaction while on treatment in children with persistent cow's milk allergy, from around 4–5 years of age” and that “no recommendation can be made about OIT as a treatment option in adults with persistent cow's milk allergy”.^80^

The authors stated that a “combination of OIT with biologicals (such as omalizumab) may enhance safety of immunotherapy” and that “in patients with systemic reactions, individualized schedules with a longer and slower up-dosing phase, and premedication (antihistamines, or omalizumab) may be considered”. The EAACI guidelines do not comment on the use of OIT with baked milk or the epicutaneous immunotherapy.

Expert panel selected from members of the Spanish Society of Pediatric Allergology, Asthma and Clinical Immunology (SEICAP) and the Spanish Society of Allergology and Clinical Immunology (SEAIC) published the Spanish guidelines on OIT for food allergy in 2017.^81^ The authors concluded that “OIT is effective in inducing desensitization in most patients with IgE-mediated CM and egg allergy, although the results in terms of long-term tolerance are not clear”. The guideline also mentions immunotherapy with baked milk but there are no specific recommendations: “in patients who do not tolerate extensively heated milk, desensitization has been attempted with this form of the product (ie, baked milk), although with little success”. The guidelines do not comment on the use of omalizumab with OIT or the epicutaneous immunotherapy.

## Revision or adaptation of these guidelines

After publication of these guidelines, WAO will maintain them through surveillance for new evidence, ongoing review by experts, and regular revisions.

Adaptation of these guidelines may be necessary in many circumstances. We encourage all stakeholders who would like to adapt the recommendations to their local circumstances to use the attached evidence-to-decision tables (see Online Supplement 1) and to follow the systematic and transparent GRADE-ADOLOPMENT process.^26^

## Abbreviations

CI, confidence interval; CMA, cow’s milk allergy; EoE, eosinophilic esophagitis; EPIT, epicutaneous immunotherapy; GRADE, Grading of Recommendations Assessment, Development, and Evaluation; IgE-CMA, IgE-mediated cow’s milk allergy; non-IgE-CMA, non-IgE-mediated cow’s milk allergy; OIT, oral immunotherapy; RD, risk difference; RR, relative risks; IgE, specific immunoglobulin E; SPT, skin prick test; WAO, World Allergy Organization.

## Availability of materials

All data generated or analyzed during this study are included in this published article (and its supplementary information files).

## Author contributions

**Study conception and design:** Alessandro Fiocchi, Holger J. Schünemanna, Jan L Brozek.

**Data acquisition:** Jan L. Brozek, Ramon T. Firmino, Antonio Bognanni, Stefania Arasi, Derek K Chu, Piotr Dziechciarz, Andrea Horvath, Yetiani Roldan, Siw Waffenschmidt.

**Data analysis and generation of Evidence-to-decision tables:** Jan L. Brozek, Ramon T. Firmino, Antonio Bognanni, Stefania Arasi.

**Evidence interpretation and generation of recommendations:** Jan L. Brozek, Ramon T. Firmino, Antonio Bognanni, Stefania Arasi.

**Evaluation of recommendations and rating of their importance:** Ignacio Ansotegui, Amal H. Assa'ad, Sami L. Bahna, Roberto Berni Canani, Martin Bozzola, Lamia Dahdah, Christophe Dupont, Motohiro Ebisawa, Elena Gall, Rose Kamenwa, Gideon Lack, Haiqi Li, Alberto Martelli, Anna Nowak-Węgrzyn, Nikolaos G. Papadopoulos, Ruby Pawankar, Maria Said, Mario Sánchez-Borges, Raanan Shamir, Jonathan M. Spergel, Hania Szajewska, Luigi Terracciano, Yvan Vandenplas, Carina Venter, Susan Waserman, Amena Warner, Gary W. K. Wong, Alessandro Fiocchi.

**First draft:** Jan L. Brozek, Ramon T. Firmino, Antonio Bognanni, Stefania Arasi.

**Manuscript revising:** Jan L. Brozek, Ramon T. Firmino, Antonio Bognanni, Ignacio Ansotegui, Stefania Arasi, Amal H. Assa'ad, Sami L. Bahna, Roberto Berni Canani, Martin Bozzola, Derek K. Chu, Lamia Dahdah, Christophe Dupont, Piotr Dziechciarz, Motohiro Ebisawa, Elena Gall, Andrea Horvath, Rose Kamenwa, Gideon Lack, Haiqi Li, Alberto Martelli, Anna Nowak-Węgrzyn, Nikolaos G. Papadopoulos, Ruby Pawankar, Yetiani Roldan, Maria Said, Raanan Shamir, Jonathan M. Spergel, Hania Szajewska, Luigi Terracciano, Yvan Vandenplas, Carina Venter, Siw Waffenschmidt, Susan Waserman, Amena Warner, Gary W. K. Wong, Alessandro Fiocchi, Holger J. Schünemann.

All authors reviewed and approved the final manuscript.

## Funding

World Allergy Organization and the McMaster University GRADE Centre.

## Consent for publication

All authors approved the final version and agreed to publication in the World Allergy Organization Journal.

## Ethics approval

Not applicable for this work.

## Declaration of competing interest

Some of the authors have professional affiliations or personal interests outside of the submitted work that are related to the topic of allergy. During the development of these guidelines, an external committee was asked to review each research team and panel member's anonymized disclosure of interests. Panel members who were deemed to have a real, perceived, or potential conflict of interest were asked to abstain from voting on recommendations related to that interest.

The DRACMA CoI policy has been uploaded as supplementary file.

The following authors, in conflict of interest, did not participate to the assessment of the following questions:

Question 2 – Omalizumab in OIT: Gideon Lack, Nikos Papadopoulos, Alessandro Fiocchi (CoI Novartis), Question 4: epicutaneous IT with milk vs. OIT with milk: Christophe Dupont, Motohiro Ebisawa, Gideon Lack, Jonathan Spergel, Carina Venter (CoI - DBV technologies).

S Arasi, S Bahna, A Bognanni, J Brozek, D Chu, L Dahdah, P Dziechciarz, E Galli, A Horvath, R Kamenwa, H Li, A Martelli, R Pawankar, H Schunemann, R Targino, L Terracciano, A Warner, and S Waffenschmidt have no conflicts to disclose.

IJ Anstotegui – Abbott, Amgen, Astra Zeneca, Bayer, Bial, Faes Farma, Hikma, Menarini, Merck, Mundipharma, Roxall, Sanofi, Stallergenes, UCB.

A Assa'ad – Aimmune Therapeutics, DBV Technologies, Astella, ABBVIE, Novartis, Sanofi, FARE, NIH and an intellectual property patent licensed to Hoth.

R Berni Canani – Nutricia, Ch.Hansen, Danone, DVB, Humana, iHealth, Kraft Heinz, Mead Johnson, Nestlè, Novalac, Sanofi.

M Bozzola – Danone.

C Dupont – Nestle Health Science, Nestle France, Nutricia, Novalac, Sodilac, Abbott, Danone, and stock ownership at DBV Technologies.

M Ebisawa – DBV Technologies, Mylan, ARS Pharmaceuticals, Novartis.

A Fiocchi – Abbott, Danone.

G Lack – FARE, National Peanut Board (NPB), The Davis Foundation, Action Medical Research, UK Food Standards Agency, Medical Research Council, DBV Technologies, Mission Mighty Me, Novartis, Sanofi-Genyzme, Regeneron, ALK-Abello, Lurie Children's Hospital.

A Nowak-Wegrzyn – Nestle, Nutricia, Novartis, Gerber, Aimmune.

N Papadopoulos – Novartis, Nutricia, HAL Allergy, Menarini/Faes Farma, Sanofi, Mylan/Meda, Biomay, AstraZeneca, GSK, MSD, ASIT Biotech, Boehringer Ingelheim, Gerolymatos International SA, Capricare.

M Said – Nestle, Nutricia, Abbott, Bayer for Anaphylaxis Australia.

J Spergel – DBV Technologies, Regeneron, Sanofi, and Aimmune.

H Szajewska – Ausnutria, Cargill, Danone, Else Nutrition, Hipp, Nestle, and Nestle Nutrition Institute.

Y Vandenplas – Abbott Nutrition, Biogaia, Biocodex, By Heart, CHR Hansen, Danone, ELSE Nutrition, Friesland Campina, Hero, Hypocrata, Nestle Health Science, Nestle Nutrition Institute, Nutricia, Mead Johnson Nutrition, Orafti, Phacobel, Phathom Pharmaceuticals, Sari Husada, United Pharmaceuticals (Novalac), Wyeth, Yakult.

C Venter – Reckitt Benckiser, Nestle Nutrition Institute, Danone, Abbott Nutrition, Else Nutrition, and Before Brands, DBV Technologies.

S Waserman – Novartis-basic science work on peanut allergy, Aimmune-peanut OIT trial, Medical Advisor to Food Allergy Canada, and Pfizer, Bausch, Kaleo-consultant for epinephrine autoinjectors.

GWK Wong – Nestle, Danone.
