# Supplementary material for: World Allergy Organization (WAO) Diagnosis and Rationale for Action against Cow’s Milk Allergy (DRACMA) Guideline update – XIV – Recommendations on CMA immunotherapy
Source: World Allergy Organ J. 2022 Apr 23;15(4):100646. doi: 10.1016/j.waojou.2022.100646 (PMC9061625; doi:10.1016/j.waojou.2022.100646)
Supplement: Multimedia component 2 [file mmc2.doc]

**CMA from diagnosis to treatment - Policy on Independence and Scientific Decision-Making Processes.**

This document identifies the specific independence policies to be used for DRACMA guidelines.

**Why a policy on independence and scientific decision-making processes?**

This policy describes all the steps that have been taken by WAO to ensure the implementation of its core values in the scientific outputs and decision-making process for the DRACMA guidelines. It aims to assure high-quality scientific outputs based on transparent, open and unbiased scientific decision-making processes.

**WAO core values**

As an international umbrella organization, the World Allergy Organization (WAO) convenes professionals from all over the world to achieve WAO’s mission to be a global resource and advocate in the field of allergy and asthma to advance excellence in clinical care, education, research, and training. All WAO programs and initiatives strive to be global in scope to provide venues and forums for international knowledge sharing and networking.

As part of its mission to advance the field of allergy and asthma worldwide and provide scholarly resources of global importance for its 97 member societies, WAO would like to write a supplement to its 2010 Guideline for the Diagnosis and Rationale for Action against Cow’s Milk Allergy (DRACMA).

**The new DRACMA guidelines**

This new DRACMA guideline will act as a reference for the diagnosis and treatment of IgE as well as non- IgE mediated Cow’s Milk Allergy (CMA). The goal of this guideline is to provide clinicians, including pediatric GI physicians, and their patients with guidelines based on scientific evidence and to unify the language to prevent further confusion. WAO is working in partnership with McMaster University and the Grading of Recommendations Assessment, Development, and Evaluation (GRADE) working group on this guideline. WAO did establish a scientific board (DRACMA Scientific Committee) with the aim of developing materials to overcome all the barriers to CMA guideline implementation.

**Collegial decision making**

According to the GRADE rules, DRACMA Scientific Committee is formed by experts with a wide range of complementary skills and experiences, drawn from diverse backgrounds ranging from specialists to patient organizations. The recommendations will be adopted by consensus or by majority decision following a metanalytic revision of the literature. The process does provide room for contradictory debates at the preparation and final decision level. Thus, the risk of one viewpoint exerting an undue influence over the other members of the group will be limited and the DRACMA guidelines will not represent the views of any single expert or school of thought. As a last resort, experts who will not agree with the majority of their peers may will adopt a duly reasoned minority opinion, where they will explain the reasons for a divergent position. DRACMA will record all minority views and will publish them in its scientific outputs to ensure that the full plurality of views is transparently reflected in its advice. The quality of DRACMA’s scientific outputs is also enhanced by ensuring a shared responsibility of all members of a Panel and competent Working Group in relation to the preparatory work.

**Declaration of CoI**

The DRACMA panel recognises that scientific expertise underpins the fulfilment of its mission and tasks and that the quality of such expertise is inherently based on prior experience. An “interest” declared is not automatically considered a conflict of interest. In general, individuals who are involved in a particular process inherently have a professional interest in the subject and in being involved in the process as such. Therefore, members of DRACMA panel have a professional interest in the work they are undertaking and in the outcome of these activities.

**Nature of the declared activities**

1. **Ownership or other investments**, including shares: it indicates meaning any financial interests in a company/entity whose product or substance is being reviewed, or a company that manufactures or markets products used in an activity falling within the DRACMA remit. This includes holding of stocks and shares, equity, bonds, partnership interests in the capital of a company, one of its subsidiaries or a company of which it has a holding.
2. **Member of a Managing Body** is to be interpreted as meaning any participation in the internal decision-making of a company, trade association or equivalent entity (e.g. board membership, directorship) whose product are reviewed in DRACMA.
3. **Member of a Scientific Advisory Body** is to be interpreted as meaning that the person concerned is participating or has participated, with a right to vote on the outputs, in the works of a Scientific Advisory Body which has expressed an opinion, a statement or an advice about products at issue.
4. **Employment** is to be interpreted, as covering all forms of employment, part-time and full-time, either paid or unpaid, in any organisation (private or public) whose product or substance is being reviewed.
5. **Consultancy/Advice** is to be interpreted as an activity where the concerned person charges or does not charge a fee for providing advice or services in a particular field such as (1) the development of the product, (2) a competitor product or a product used in conjunction either with the one being reviewed.
6. **Research funding** is to be interpreted as meaning any funding for research on the development of the product being reviewed or the one from a competitor, if financed by a private or public entity, including grants, rents, sponsorships and fellowships and received in a personal capacity.
7. **Intellectual property rights** are to be interpreted as meaning rights granted to creators and owners of works that are the result of human intellectual creativity. These can be publications or can be in the industrial, scientific and artistic domain. They can be in the form of an invention, a document, a suite of software, or a business name (e.g. copyrights, trademarks, patents on the product or substance or a competitor product or substance or a substance or product used in conjunction with the one being reviewed).
8. **Interests of close family members** are to be interpreted as meaning inter alia known interests held by family members and relatives belonging to the same household or under the care of the members of the household, and that relate to the development of the product being reviewed. The relationship (e.g. wife) should not be specified.
9. **Other** is to be interpreted as meaning that the person concerned has any activities or interests other than the above that can be perceived as an interest in the field of activity of DRACMA.

Other definitions

• **Current** is to be interpreted as meaning the activities that are currently ongoing.

• **Past period** is to be interpreted as meaning activities that are no longer ongoing and which have been completed in the five years preceding the filling in of the DoI.

• **Name of entity or organization** is to be interpreted as meaning name, location and nature of all organisations (private, public, etc.) that relate to the item on the agenda or in the mandate. Thus, for the purpose of the declarations of interests the involvement in public bodies needs to be included as well.

• **Item on the agenda or in the mandate** is to be interpreted as meaning the item(s) in the agenda or the mandate that is of concern e.g. types of substances, products, guidance documents, processes or policies. Any data collection and any other interest stemming from prior experience or affiliation of the individual with private or public institution should be equally declared.

**Compatibility of the declared activities**

The following will be considered incompatible with the participation to the DRACMA process:

1. **Ownership or other investments**, over $ 100,000;
2. **Membership of a Managing Body.**
3. **Employment**

The following will be considered incompatible with the participation to the DRACMA votations for diagnosis:

1. **Member of a Scientific Advisory Body** in diagnostic products for CMA
2. **Consultancy/Advice** in diagnostic products for CMA
3. **Research funding** in diagnostic products for CMA
4. **Intellectual property rights** in diagnostic products for CMA
5. **Interests of close family members** in diagnostic products for CMA

The following will be considered incompatible with the participation to the DRACMA votations for treatment recommendations:

1. **Member of a Scientific Advisory Body** in special formulae for CMA
2. **Consultancy/Advice** in special formulae for CMA
3. **Research funding** in special formulae for CMA
4. **Intellectual property rights** in special formulae for CMA
5. **Interests of close family members** in special formulae for CMA

Rome, September 27^th^ 2017
