# Supplementary material for: World Allergy Organization (WAO) Diagnosis and Rationale for Action against Cow’s Milk Allergy (DRACMA) Guideline update – XIV – Recommendations on CMA immunotherapy
Source: World Allergy Organ J. 2022 Apr 23;15(4):100646. doi: 10.1016/j.waojou.2022.100646 (PMC9061625; doi:10.1016/j.waojou.2022.100646)
Supplement: Multimedia component 3 [file mmc3.pdf]

Supplementary material

for the World Allergy Organization (WAO) Diagnosis and Rationale for Action against Cow’s Milk Allergy (DRACMA) Guidelines 2021: immunotherapy

Table of contents

	Page
Evidence-to-Decision table for question 1: Should oral immunotherapy with cow's milk vs. no immunotherapy be used for IgE-mediated cow's milk allergy?	2
Evidence-to-Decision table for question 2: Should omalizumab vs. no anti-IgE antibody be used for patients who receive OIT for IgE-mediated CMA?	8
Evidence-to-Decision table for question 3: Should OIT with baked milk vs. no OIT be used for patients with IgE-CMA who do not tolerate baked milk?	14
Summary of findings table for question 1: Should oral immunotherapy with cow's milk vs. no immunotherapy be used for IgE-mediated cow's milk allergy?	20
Summary of findings table for question 2: Should omalizumab vs. no anti-IgE antibody be used for patients who receive OIT for IgE-mediated CMA?	26
Summary of findings table for question 3: Should OIT with baked milk vs. no OIT be used for patients with IgE-CMA who do not tolerate baked milk?	30
Summary of findings table for question 4: Should epicutaneous immunotherapy vs. no immunotherapy be used for IgE-mediated cow's milk allergy?	32
Membership of the panel and the evidence synthesis team	33

Evidence-to-Decision table for question 1

Should oral immunotherapy with cow's milk vs. no immunotherapy be used for IgE-mediated cow's milk allergy?

QUESTION

Should oral immunotherapy with cow's milk vs. no immunotherapy be used for IgE-mediated cow's milk allergy?	
**POPULATION:**	IgE-mediated cow's milk allergy
**INTERVENTION:**	oral immunotherapy with cow's milk
**COMPARISON:**	no immunotherapy
**MAIN OUTCOMES:**	Anaphylaxis; Use of IM epinephrine; Discontinuation of treatment due to adverse effects and/or symptoms; Gastrointestinal symptoms (severe); Severe respiratory symptoms/wheezing; Generalized erythema or urticaria; Angioedema; Ability to drink cow's milk and eat dairy products without a reaction; Ability to accidentally consume a small amount of cow's milk without a reaction; Duration of sustained tolerance of milk (when achieved); Emergency department visit; Death; Any adverse effects; Mild respiratory symptoms; Mild laryngospasm; Lip/mouth pruritus; Hospital admission; Eosinophilic esophagitis; Quality of life of children; Quality of life of the caregivers;
**SETTING:**	allergy specialty clinics
**PERSPECTIVE:**	individual patient
**COMPETING INTERESTS:**	none

ASSESSMENT

**Desirable Effects**<br>How substantial are the desirable anticipated effects?		
**JUDGEMENT**	**RESEARCH EVIDENCE**	**ADDITIONAL CONSIDERATIONS**
○ Trivial<br>○ Small<br>● Moderate<br>○ Large<br>○ Varies<br>○ Don't know	Please see the corresponding evidence profile.	Panel members thought that the ability to accidentally consume a small amount of cow's milk or milk products without a reaction is the main benefit of OIT by protecting patients from accidental anaphylaxis in daily life. Patients would still have to avoid milk, but much less strictly.<br>Half of panel members thought that the benefits were moderate and the other half that they were large.
**Undesirable Effects**<br>How substantial are the undesirable anticipated effects?		
**JUDGEMENT**	**RESEARCH EVIDENCE**	**ADDITIONAL CONSIDERATIONS**
○ Large<br>● Moderate<br>○ Small	Please see the corresponding evidence profile.	Majority of panel members thought that the undesirable effects were moderate and some thought they were large.

○ Trivial<br>○ Varies<br>○ Don't know		

Certainty of evidence

What is the overall certainty of the evidence of effects?

JUDGEMENT	RESEARCH EVIDENCE	ADDITIONAL CONSIDERATIONS
○ Very low<br>○ Low<br>● Moderate<br>○ High<br>○ No included studies	Please see the attached evidence profile<br>This is the lowest certainty across the critical outcomes. There is moderate certainty about both desirable and undesirable outcomes.	

Values

Is there important uncertainty about or variability in how much people value the main outcomes?

JUDGEMENT	RESEARCH EVIDENCE	ADDITIONAL CONSIDERATIONS
○ Important uncertainty or variability<br>● Possibly important uncertainty or variability<br>○ Probably no important uncertainty or variability<br>○ No important uncertainty or variability	**Students with food allergy perceive a tension between maintaining their social wellbeing and physical safety, expressing concern about the social implications of food allergy and interventions to manage it.**<br>*1. Dean J, Fenton NE, Shannon S, Elliott SJ, Clarke A. Disclosing food allergy status in schools: health-related stigma among school children in Ontario. Health Soc Care Community 2016;24:e43-52.*<br>**Among children with food allergy, there is variability in risk perception, risk-taking behaviors, the level of concern they express about having food allergy, and how they balance threats to their social identity with threats to their personal safety.**<br>*2. Akeson N, Worth A, Sheikh A. The psychosocial impact of anaphylaxis on young people and their parents. Clin Exp Allergy 2007;37:1213-20.*<br>*3. Cummings AJ, Knibb RC, Erlewyn-Lajeunesse M, King RM, Roberts G, Lucas JSA. Management of nut allergy influences quality of life and anxiety in children and their mothers. Pediatr Allergy Immunol 2010;21:586-94.*<br>*4. Monks H, Gowland MH, MacKenzie H, Erlewyn-Lajeunesse M, King R, Lucas JS, et al. How do teenagers manage their food allergies? Clin Exp Allergy 2010;40:1533-40.*<br>*5. Fenton NE, Elliott SJ, Cicutto L, Clarke AE, Harada L, McPhee E. Illustrating risk: anaphylaxis through the eyes of the food-allergic child. Risk Anal 2011;31:171-83.*<br>*6. Sampson MA, Munoz-Furlong A, Sicherer SH. Risk-taking and coping strategies of adolescents and young adults with food allergy. J Allergy Clin Immunol 2006;117:1440-5.*<br>*7. Sommer I, Mackenzie H, Venter C, Dean T. An exploratory investigation of food choice behavior of teenagers with and without food allergies. Annals of Allergy, Asthma and Immunology 2014;112:446-52.*	Panel members agreed that patients with IgE-mediated CMA place **high value on avoiding severe and fatal allergic reactions**. However, **there might be important variability in how they value other outcomes.** For example, some school-aged patients may place more or less value on the ability to drink milk and eat dairy relative to the ability to take part in social activities (e.g. OIT might preclude going on school trips that would require missing one or more daily doses of OIT, making OIT too difficult or not feasible).<br>Some older patients are likely to **vary in their perception of burden** related to OIT: e.g. avoiding exercise after taking a daily dose of OIT or requirement for regular daily dosing.<br>**Patient and family goals may differ**: some may value more the ability to drink milk, others may just wish to avoid an allergic reaction.

Balance of effects

Does the balance between desirable and undesirable effects favor the intervention or the comparison?

JUDGEMENT	RESEARCH EVIDENCE	ADDITIONAL CONSIDERATIONS

○ Favors the comparison<br>○ Probably favors the comparison<br>○ Does not favor either the intervention or the comparison<br>○ Probably favors the intervention<br>○ Favors the intervention<br>● Varies<br>○ Don't know	Please see the attached evidence profile	Panel members thought that the overall balance of effects does not favor either intervention. However, they acknowledged that it mostly depends on values and preferences that patients and/or their caregivers assign to particular outcomes. For those who value more the ability to drink milk, compared with advese efects during OIT, tha balance may favor OIT. For those who place more value on avoiding allergic reactions, the balance may favor staying on elimination diet without OIT.

Resources required

How large are the resource requirements (costs)?

JUDGEMENT	RESEARCH EVIDENCE	ADDITIONAL CONSIDERATIONS
● Large costs<br>○ Moderate costs<br>○ Negligible costs and savings<br>○ Moderate savings<br>○ Large savings<br>○ Varies<br>○ Don't know	No research evidence was found.	Panel members noted that the **estimates of the direct and indirect costs of OIT with milk are currently not available.**<br>Based on personal experience of panel members, cost of OIT is likely to be large, because it requires trained health care professionals and clinical facilities in order to provide OIT, and the availability of emergency physician to provide advice during maintenance at home.<br>Majority of panel members thought that the additional costs of OIT are large and others thought that they were moderate.

Equity

What would be the impact on health equity?

JUDGEMENT	RESEARCH EVIDENCE	ADDITIONAL CONSIDERATIONS
○ Reduced<br>○ Probably reduced<br>○ Probably no impact<br>○ Probably increased<br>○ Increased<br>● Varies<br>○ Don't know	No research evidence was found.	**OIT is currently not reimbursed in many countries** and many third-party health insurance systems do not cover it, so **it would be available only to more affluent families** that could cover the cost out of the pocket or through an expensive health insurance.<br>Cost and availability of specialized facilities to perform OIT are more likely to limit implementation in jurisdictions where fewer resources are available.<br>Panel members thought that the impact on health equity would vary depending on who bears the cost of OIT (patients and families themselves, public health care system, or private third party payers) and whether or not all or only selected more expensive insurance systems would cover OIT.

Acceptability

Is the intervention acceptable to key stakeholders?

JUDGEMENT	RESEARCH EVIDENCE	ADDITIONAL CONSIDERATIONS
○ No<br>○ Probably no<br>○ Probably yes<br>○ Yes<br>● Varies<br>○ Don't know	No research evidence was found.	The **autonomy of patients** (e.g. not doing physical exercises after OIT) as well as parents (need to supervise the child) may be affected.<br>**Clinicians as well as family members** may vary in their perception of risk and the relative value they place on avoiding reactions with accidental exposure to milk or with OIT. Thus, some clinicians and family members may be reluctant to administer or accept OIT while others will not. Some **clinicians** may not accept the risk of allergic reactions that occur during OIT in their offices.

		**Preschool and school personnel** may not accept providing and/or supervising milk OIT during school trips.<br>**Third party payers** may not accept the additional cost of specialized health care personnel and clinical facilities required for OIT.<br>The long-term effects and persistence of desensitization are still being investigated. This uncertainty influences the variability in acceptance of OIT by patients, their families, clinicians, and third party payers. The general perception of importance of food allergy varies across cultures and also affects the acceptability of related interventions.

Feasibility

Is the intervention feasible to implement?

JUDGEMENT	RESEARCH EVIDENCE	ADDITIONAL CONSIDERATIONS
○ No<br>○ Probably no<br>● Probably yes<br>○ Yes<br>○ Varies<br>○ Don't know	No research evidence was found.	Panel members found the following to be currently the **main barriers to implementation:**<br>- **additional cost** of OIT<br>- **limited availability** and access to OIT in many countries<br>- limited and **variable availability of facilities** for OIT<br>- **limited availability of allergy specialists** who would have to provide and supervise OIT<br>- **need for education and training for patients** and their families<br>- need to start OIT in a hospital (in settings in which it is required).<br>School personnel is unlikely to provide or supervise OIT if it needs to be done on a school trip. Parents or other caregivers would have to accompany children on those occasions.<br>**Lifelong or long-term OIT may not be sustainable** owing to its cumulative cost and burden.<br>The **inappropriate use** of milk OIT would increase the risk of serious adverse effects in children with severe food allergies. However, it would be unlikely if it was used in patients correctly diagnosed with IgE-mediated CMA and properly administered by allergy specialists.

SUMMARY OF JUDGEMENTS

	JUDGEMENT						
**DESIRABLE EFFECTS**	Trivial	Small	**Moderate**	Large	–	Varies	Don't know
**UNDESIRABLE EFFECTS**	Large	**Moderate**	Small	Trivial	–	Varies	Don't know
**CERTAINTY OF EVIDENCE**	Very low	Low	**Moderate**	High	–		No included studies
**VALUES**	Important uncertainty or variability	**Possibly important uncertainty or variability**	Probably no important uncertainty or variability	No important uncertainty or variability	–	–	–

	JUDGEMENT						
BALANCE OF EFFECTS	Favors the comparison	Probably favors the comparison	Does not favor either the intervention or the comparison	Probably favors the intervention	Favors the intervention	**Varies**	Don't know
RESOURCES REQUIRED	**Large costs**	Moderate costs	Negligible costs and savings	Moderate savings	Large savings	Varies	Don't know
EQUITY	Reduced	Probably reduced	Probably no impact	Probably increased	Increased	**Varies**	Don't know
ACCEPTABILITY	No	Probably no	Probably yes	Yes	–	**Varies**	Don't know
FEASIBILITY	No	Probably no	**Probably yes**	Yes	–	Varies	Don't know

TYPE OF RECOMMENDATION

Strong recommendation against the intervention	Conditional recommendation against the intervention	Conditional recommendation for either the intervention or the comparison	Conditional recommendation for the intervention	Strong recommendation for the intervention
○	○	●	○	○

CONCLUSIONS

Recommendation

Recommendation 1A
We suggest oral immunotherapy with unheated cow's milk, rather than no immunotherapy, for those people with IgE-mediated CMA who place a higher value on being able to consume milk (even small amounts) with less need to follow a strict avoidance diet, and a lower value on allergic reactions during OIT.
(CONDITIONAL recommendation based on moderate certainty evidence about health effects)

Recommendation 1B
We suggest that clinicians do not use oral immunotherapy with unheated cow's milk in those people with IgE-mediated CMA who place a higher value on avoiding allergic reactions during OIT, and a lower value on being able to consume cow's milk (even small amounts) with less need to follow a strict avoidance diet.
(CONDITIONAL recommendation based on moderate certainty evidence about health effects)

Justification

Panel members thought that the choice whether to accept OIT will mostly depend on the value that they place on particular outcomes.

Subgroup considerations

Patients with persistent reactions who are unlikely to outgrow CMA may benefit from OIT more than those who are still likely to outgrow it.

Implementation considerations

Diagnosis of IgE-mediated CMA must be confirmed before commencing milk OIT.
When choosing to preform OIT, clinicians may want to consider the following situations that may be contraindications for starting and for continuation of OIT:
- a patient and/or the family are not able to follow the OIT protocol for any reason (e.g., scheduling conflicts, patient's athletic activities)

- a patient and/or their family have no access to epinephrine and/or are not able to properly use it when needed
- a patient has a confirmed history of previous frequent severe reactions
- a patient had multiple severe reactions to cow's milk OIT
- a patient has persistent gastrointestinal symptoms
- a patient has a concomitant asthma that is not well controlled
- a physician suggesting to use OIT is not able to devote sufficient time and resources to properly administering and monitoring OIT – this may require a 24 hours per day, 7 days per week on-call service
- a preschool or school personnel does not accept providing and/or supervising milk OIT during school trips which might require the child to forgo school social activities or temporarily suspend the OIT

Monitoring and evaluation

When choosing to preform OIT, clinicians need to monitor the symptoms in all patients and proper nutrition in small children.

Research priorities

The panel identified the following priorities for research in this area:

1. Properly designed and executed experimental studies (RCTs) in patients with moderate and *severe* CMA (including those with previous severe anaphylaxis) that would measure and report *all important outcomes,* and that would investigate:

- sustainability of the long-term beneficial effects
- short-term and long-term adverse effects
- relative effects of different doses (especially the staring dose) and different protocols of OIT to identify the best balance between desirable and undesirable effects of OIT
- the effects of OIT with unheated milk compared with baked milk.

2. Studies are also needed to provide more information about:

- predictors of response to OIT
- resources required to offer OIT and its cost-effectiveness.

3. Qualitative studies of patients' and their families' knowledge about CMA and OIT and understanding the benefits and risks, and their expectations from the management of milk allergy (values and preferences).

Should omalizumab vs. no anti-IgE antibody be used for patients who receive OIT for IgE-mediated CMA?

QUESTION

Should omalizumab vs. no anti-IgE antibody be used for patients who receive OIT for IgE-mediated CMA?	
**POPULATION:**	patients who receive OIT for IgE-mediated CMA
**INTERVENTION:**	omalizumab
**COMPARISON:**	no anti-IgE antibody
**MAIN OUTCOMES:**	Anaphylaxis RCT; Anaphylaxis (Observational studies); Use of IM epinephrine (adrenaline) RCT; Use of IM epinephrine (adrenaline) NRS; Adverse effect leading to the discontinuation of treatment; Adverse effect leading to the discontinuation of treatment OBS; Severe gastrointestinal symptoms RCT; Severe gastrointestinal symptoms OBS; Severe respiratory symptoms/wheezing RCT; Severe respiratory symptoms/wheezing OBS; Generalized erythema or urticaria RCT; Generalized erythema or urticaria OBS; Ability to drink cow's milk and eat dairy products without a reaction; Ability to accidentally consume a small amount of cow's milk without a reaction; Ability to drink cow's milk reintroduced after a period of not consuming milk and milk products; Ability to accidentally consume a small amount of cow's milk reintroduced after a period of not consuming milk and milk products; Emergency department visit RCT; Emergency department visit OBS; Hospital admission; Hospital admission OBS; Eosinophilic esophagitis; Quality of life of patients; Quality of life of the caregivers; Any adverse effect;
**SETTING:**	tertiary care allergy clinic
**PERSPECTIVE:**	individual patient
**COMPETING INTERESTS:**	Two panel members were deemed to have an actual, potential, or perceived conflict of interest and abstained from voting on this recommendation: Gideon Lack and Nikolaos Papadopoulos

ASSESSMENT

Desirable Effects<br>How substantial are the desirable anticipated effects?		
**JUDGEMENT**	**RESEARCH EVIDENCE**	**ADDITIONAL CONSIDERATIONS**
○ Trivial<br>○ Small<br>● Moderate<br>○ Large<br>○ Varies<br>○ Don't know	See the attached Evidence Profile	Most panel members thought that the desirable effects were moderate, despite some judging them as small or trivial. Anti-IgE allow for quicker updosing of the OIT. However, the frequency of adverse effects of OIT after the discontinuation of anti-IgE also needs to be clarified.
**Undesirable Effects**<br>How substantial are the undesirable anticipated effects?		
**JUDGEMENT**	**RESEARCH EVIDENCE**	**ADDITIONAL CONSIDERATIONS**
○ Large<br>○ Moderate<br>● Small	See the attached Evidence Profile	One panel member thought that the undesirable effects were trivial.

○ Trivial<br>○ Varies<br>○ Don't know		

Certainty of evidence

What is the overall certainty of the evidence of effects?

JUDGEMENT	RESEARCH EVIDENCE	ADDITIONAL CONSIDERATIONS
● Very low<br>○ Low<br>○ Moderate<br>○ High<br>○ No included studies	See the attached Evidence Profile<br>Certainty of evidence is the lowest rating across the critical outcomes.	

Values

Is there important uncertainty about or variability in how much people value the main outcomes?

JUDGEMENT	RESEARCH EVIDENCE	ADDITIONAL CONSIDERATIONS
○ Important uncertainty or variability<br>● Possibly important uncertainty or variability<br>○ Probably no important uncertainty or variability<br>○ No important uncertainty or variability	**Children with food allergy perceive a tension between maintaining their social wellbeing and physical safety, expressing concern about the social implications of food allergy and interventions to manage it.**<br>*1. Dean J, Fenton NE, Shannon S, Elliott SJ, Clarke A. Disclosing food allergy status in schools: health-related stigma among school children in Ontario. Health Soc Care Community 2016;24:e43-52.*<br><br>**Among children with food allergy, there is variability in risk perception, risk-taking behaviors, the level of concern they express about having food allergy, and how they balance threats to their social identity with threats to their personal safety.**<br>*2. Akeson N, Worth A, Sheikh A. The psychosocial impact of anaphylaxis on young people and their parents. Clin Exp Allergy 2007;37:1213-20.*<br>*3. Cummings AJ, Knibb RC, Erlewyn-Lajeunesse M, King RM, Roberts G, Lucas JSA. Management of nut allergy influences quality of life and anxiety in children and their mothers. Pediatr Allergy Immunol 2010;21:586-94.*<br>*4. Monks H, Gowland MH, MacKenzie H, Erlewyn-Lajeunesse M, King R, Lucas JS, et al. How do teenagers manage their food allergies? Clin Exp Allergy 2010;40:1533-40.*<br>*5. Fenton NE, Elliott SJ, Cicutto L, Clarke AE, Harada L, McPhee E. Illustrating risk: anaphylaxis through the eyes of the food-allergic child. Risk Anal 2011;31:171-83.*<br>*6. Sampson MA, Munoz-Furlong A, Sicherer SH. Risk-taking and coping strategies of adolescents and young adults with food allergy. J Allergy Clin Immunol 2006;117:1440-5.*<br>*7. Sommer I, Mackenzie H, Venter C, Dean T. An exploratory investigation of food choice behavior of teenagers with and without food allergies. Annals of Allergy, Asthma and Immunology 2014;112:446-52.*	Panel members agreed that patients with IgE-mediated CMA place **high value on avoiding severe and fatal allergic reactions**. However, **there might be important variability in how they value other outcomes.** For example, some school-aged patients may place more or less value on the ability to drink milk and eat dairy relative to the ability to take part in social activities (e.g. OIT might preclude going on school trips that would require missing one or more daily doses of OIT, making OIT too difficult or not feasible).<br>Some older patients are likely to **vary in their perception of burden** related to OIT: e.g. avoiding exercise after taking a daily dose of OIT or requirement for regular daily dosing.<br>**Patient and family goals may differ**: some may value more the ability to drink milk, others may just wish to avoid an allergic reaction.<br>Children would prefer to avoid injections.

Balance of effects

Does the balance between desirable and undesirable effects favor the intervention or the comparison?

JUDGEMENT	RESEARCH EVIDENCE	ADDITIONAL CONSIDERATIONS
○ Favors the comparison<br>○ Probably favors the comparison<br>○ Does not favor either the intervention or the	See the attached Evidence Profile	Majority of panel members thought that the balance of efects favors omalizumab.

comparison<br>● Probably favors the intervention<br>○ Favors the intervention<br>○ Varies<br>○ Don't know		

Resources required

How large are the resource requirements (costs)?

JUDGEMENT	RESEARCH EVIDENCE	ADDITIONAL CONSIDERATIONS
● Large costs<br>○ Moderate costs<br>○ Negligible costs and savings<br>○ Moderate savings<br>○ Large savings<br>○ Varies<br>○ Don't know	The use of Omalizumab results in an increase in costs. The mean cost of this drug in children over 6 years old has been estimated to be € 1,311 per month in Italy (Valluzzi, 2019).<br>In Spain median monthly cost of adding omalizumab to OIT was €1,100 (€738–€2,952) per patient, including the initial dose. (Larrosa Garcia 2019).	In some countries the treatment costs may be covered by the health system or reimbursed by insurance if the child also has refractory asthma.<br>The high cost of Omalizumab can limit global availability of this treatment.<br>High costs may also lead families to not complete treatment.<br>Specialized health professionals and clinical facilities are needed.<br>Cost of omalizumab is different in different countries/jurisdictions and also may depend on who bears the cost.<br>Omalizumab is usually used for 4 months in the protocols, so the total cost would be 4x monthly cost. Some patients may require longer administration.<br>Panel members also noted that the estimates of the direct and indirect costs of OIT with milk are currently not available and there is no cost effectiveness analysis of using omalizumab in OIT.

Equity

What would be the impact on health equity?

JUDGEMENT	RESEARCH EVIDENCE	ADDITIONAL CONSIDERATIONS
○ Reduced<br>● Probably reduced<br>○ Probably no impact<br>○ Probably increased<br>○ Increased<br>○ Varies<br>○ Don't know	No research evidence has been found.	**Omalizumab for OIT and OIT itself are currently not reimbursed in any country** and many third-party health insurance systems do not cover it, so **it would be available only to more affluent families** that could cover the cost out of the pocket or through an expensive health insurance.<br>Cost and availability of specialized facilities to perform OIT and administer omalizumab are more likely to limit implementation in jurisdictions where fewer resources are available.<br>Panel members thought that the impact on health equity would vary depending on who bears the cost (patients and families themselves, public health care system, or private third party payers).<br>Scarcity of specialized clinics and professionals may lead to barriers in accessing treatment (e.g.: long distance travel and costs for patients), especially in rural and remote areas, and in developing countries.

Acceptability

Is the intervention acceptable to key stakeholders?

JUDGEMENT	RESEARCH EVIDENCE	ADDITIONAL CONSIDERATIONS
o No<br>o Probably no<br>o Probably yes<br>o Yes<br>● Varies<br>o Don't know	No research evidence has been identified.	Panel members were divided whether or not the addition of omalizumab to OIT with cow's milk would be acceptable to stakeholders. The main barrier to acceptability mentioned was the cost of therapy.<br><br>Low certainty of evidence of the effect of omalizumab for CMA may reduce acceptance.<br><br>**Clinicians as well as family members** may vary in their perception of risk and the relative value they place on avoiding reactions with accidental exposure to milk or with OIT. Thus, some clinicians and family members may see value in adding omalizumab to OIT while others will not.<br>**Preschool and school personnel** may not accept providing and/or supervising milk OIT during school trips.<br>**Third party payers** may not accept the additional cost.

Feasibility

Is the intervention feasible to implement?

JUDGEMENT	RESEARCH EVIDENCE	ADDITIONAL CONSIDERATIONS
o No<br>o Probably no<br>o Probably yes<br>o Yes<br>● Varies<br>o Don't know	No research evidence has been identified.	Panel members found the following to be currently the **main barriers to implementation of omalizumab** (**in addition** to the barriers for implementation of OIT itself):<br>- **additional cost** to already expensive OIT<br>- **limited access** to omalizumab in many countries.<br><br>Currently in all jurisdictions using omalizumab for this indication would be off-label.

SUMMARY OF JUDGEMENTS

	JUDGEMENT						
DESIRABLE EFFECTS	Trivial	Small	**Moderate**	Large	–	Varies	Don't know
UNDESIRABLE EFFECTS	Large	Moderate	**Small**	Trivial	–	Varies	Don't know
CERTAINTY OF EVIDENCE	**Very low**	Low	Moderate	High	–	–	No included studies

	JUDGEMENT						
VALUES	Important uncertainty or variability	**Possibly important uncertainty or variability**	Probably no important uncertainty or variability	No important uncertainty or variability	–	–	–
BALANCE OF EFFECTS	Favors the comparison	Probably favors the comparison	Does not favor either the intervention or the comparison	**Probably favors the intervention**	Favors the intervention	Varies	Don't know
RESOURCES REQUIRED	**Large costs**	Moderate costs	Negligible costs and savings	Moderate savings	Large savings	Varies	Don't know
EQUITY	Reduced	**Probably reduced**	Probably no impact	Probably increased	Increased	Varies	Don't know
ACCEPTABILITY	No	Probably no	Probably yes	Yes	–	**Varies**	Don't know
FEASIBILITY	No	Probably no	Probably yes	Yes	–	**Varies**	Don't know

TYPE OF RECOMMENDATION

Strong recommendation against the intervention	Conditional recommendation against the intervention	Conditional recommendation for either the intervention or the comparison	Conditional recommendation for the intervention	Strong recommendation for the intervention
○	○	○	●	○

CONCLUSIONS

Recommendation

We suggest that clinicians use omalizumab, compared with not using it, during the initiation of oral immunotherapy with fresh cow's milk in people with IgE-mediated CMA.
(CONDITIONAL recommendation based on very low certainty evidence)

Justification

The balance of health effects favors adding omalizumab to milk OIT, however, cost of omalizumab may reduce its accessibility in many settings.

Subgroup considerations

None

Implementation considerations

1. Diagnosis of IgE-mediated CMA must be confirmed before commencing milk OIT.
2. When choosing to preform OIT, clinicians might consider the following situations that may be contraindications for starting and for continuation of OIT in general:
- a patient and her/his family are not able to follow the protocol
- a patient and family have no access to epinephrine and/or is not able to properly use it when needed
- a physician managing OIT is not able to devote sufficient time and resources to properly administer and monitor OIT.

- a patient has a history of confirmed previous frequent severe reactions
- a child has persistent gastrointestinal symptoms
- a patient has a concomitant asthma that is not well controlled.

3. Dosing of anti-IgE needs to be based on serum total IgE measurement.

NOTE: Patients with coexisting severe asthma and/or chronic spontaneous urticaria may be more likely to have access to omalizumab.

Monitoring and evaluation

1. Monitor symptoms after anti-IgE injection.
2. Monitoring of the OIT with anti-IgE should be the same as without it.

Research priorities

1. Dosing of omalizumab and duration of treatment with omalizumab in the context of food OIT.
2. Patient identification that would benefit the most.
3. Well designed and executed RCTs measuring important desirable and undesirable health effects and quality of life.

Evidence-to-Decision table for question 3

Should OIT with baked milk vs. no OIT be used for patients with IgE-CMA who do not tolerate baked milk?

QUESTION

Should OIT with baked milk vs. no OIT be used for patients with IgE-CMA who do not tolerate baked milk?	
**POPULATION:**	patients with IgE-CMA who do not tolerate baked milk
**INTERVENTION:**	OIT with baked milk
**COMPARISON:**	no OIT
**MAIN OUTCOMES:**	Anaphylaxis; Use of IM epinephrine; Discontinuation of treatment due to adverse effects and/or symptoms; Severe gastrointestinal symptoms; Severe respiratory symptoms/wheezing; Generalized urticaria or erythema; Ability to drink cow's milk and eat dairy products without a reaction; Ability to accidentally consume a small amount of cow's milk without a reaction; Ability to drink cow's milk reintroduced after a period of abstaining from milk and milk products; Emergency department visit; Hospital admission; Eosinophilic esophagitis; Quality of life of children; Quality of life of the caregivers; Lip/mouth pruritus; Angioedema; Any adverse effect;
**SETTING:**	Outpatient allergy clinic
**PERSPECTIVE:**	individual patient
**COMPETING INTERESTS:**	none

ASSESSMENT

Desirable Effects
How substantial are the desirable anticipated effects?

JUDGEMENT	RESEARCH EVIDENCE	ADDITIONAL CONSIDERATIONS
○ Trivial<br>● Small<br>○ Moderate<br>○ Large<br>○ Varies<br>○ Don't know	See attached evidence profile.	Some panel members thought that the effect may be larger than *small*, noting that the ability to tolerate baked milk would allow to substantially expand patient's diet. Panel members also noted that lack of controls does not allow to estimate what proportion of those who were able to eat baked milk after OIT gained it owing to OIT or naturally outgrowing milk allergy.

Undesirable Effects
How substantial are the undesirable anticipated effects?

JUDGEMENT	RESEARCH EVIDENCE	ADDITIONAL CONSIDERATIONS
○ Large<br>● Moderate<br>○ Small	See attached evidence profile.	Some panel members thought that the undesirable effects were small and others that they were large, however, the majority considered them moderate.

○ Trivial<br>○ Varies<br>○ Don't know		

Certainty of evidence

What is the overall certainty of the evidence of effects?

JUDGEMENT	RESEARCH EVIDENCE	ADDITIONAL CONSIDERATIONS
● Very low<br>○ Low<br>○ Moderate<br>○ High<br>○ No included studies	See attached evidence profile.<br>This is the lowest certainty across the critical outcomes.	Estimates of the effects of OIT with baked milk come from 2 series of cases with additional limitations.

Values

Is there important uncertainty about or variability in how much people value the main outcomes?

JUDGEMENT	RESEARCH EVIDENCE	ADDITIONAL CONSIDERATIONS
○ Important uncertainty or variability<br>● Possibly important uncertainty or variability<br>○ Probably no important uncertainty or variability<br>○ No important uncertainty or variability	**Children and adolescents with food allergy perceive a tension between maintaining their social wellbeing and physical safety, expressing concern about the social implications of food allergy and interventions to manage it.**<br>*1. Dean J, Fenton NE, Shannon S, Elliott SJ, Clarke A. Disclosing food allergy status in schools: health-related stigma among school children in Ontario. Health Soc Care Community 2016;24:e43-52.*<br>**Among children with food allergy, there is variability in risk perception, risk-taking behaviors, the level of concern they express about having food allergy, and how they balance threats to their social identity with threats to their personal safety.**<br>*2. Akeson N, Worth A, Sheikh A. The psychosocial impact of anaphylaxis on young people and their parents. Clin Exp Allergy 2007;37:1213-20.*<br>*3. Cummings AJ, Knibb RC, Erlewyn-Lajeunesse M, King RM, Roberts G, Lucas JSA. Management of nut allergy influences quality of life and anxiety in children and their mothers. Pediatr Allergy Immunol 2010;21:586-94.*<br>*4. Monks H, Gowland MH, MacKenzie H, Erlewyn-Lajeunesse M, King R, Lucas JS, et al. How do teenagers manage their food allergies? Clin Exp Allergy 2010;40:1533-40.*<br>*5. Fenton NE, Elliott SJ, Cicutto L, Clarke AE, Harada L, McPhee E. Illustrating risk: anaphylaxis through the eyes of the food-allergic child. Risk Anal 2011;31:171-83.*<br>*6. Sampson MA, Munoz-Furlong A, Sicherer SH. Risk-taking and coping strategies of adolescents and young adults with food allergy. J Allergy Clin Immunol 2006;117:1440-5.*<br>*7. Sommer I, Mackenzie H, Venter C, Dean T. An exploratory investigation of food choice behavior of teenagers with and without food allergies. Annals of Allergy, Asthma and Immunology 2014;112:446-52.*	Panel members agreed that patients with IgE-mediated CMA place **high value on avoiding severe and fatal allergic reactions**. However, **there might be important variability in how they value other outcomes.** For example, some school-aged patients may place more or less value on the ability to drink milk and eat dairy relative to the ability to take part in social activities (e.g. OIT might preclude going on school trips that would require missing one or more daily doses of OIT, making OIT too difficult or not feasible).<br>Some older patients are likely to **vary in their perception of burden** related to OIT: e.g. avoiding exercise after taking a daily dose of OIT or requirement for regular daily dosing.<br>**Patient and family goals may differ**: some may value more the ability to drink milk, others may just wish to avoid an allergic reaction.

Balance of effects

Does the balance between desirable and undesirable effects favor the intervention or the comparison?

JUDGEMENT	RESEARCH EVIDENCE	ADDITIONAL CONSIDERATIONS
○ Favors the comparison<br>● Probably favors the comparison<br>○ Does not favor either the intervention or	See attached evidence profile.	Panel members noted that the number of participants in the studies was very small and the conclusions are difficult to draw.

the comparison<br>○ Probably favors the intervention<br>○ Favors the intervention<br>○ Varies<br>○ Don't know		Currently available evidence suggests that the undesirable effects may outweigh the desirable ones. However, the certainty of the evidence is very low and further studies, if done, are likely to influence this balance.

Resources required

How large are the resource requirements (costs)?

JUDGEMENT	RESEARCH EVIDENCE	ADDITIONAL CONSIDERATIONS
● Large costs<br>○ Moderate costs<br>○ Negligible costs and savings<br>○ Moderate savings<br>○ Large savings<br>○ Varies<br>○ Don't know	No research evidence was found.	Based on personal experience of panel members, cost of OIT is likely to be **at least moderate**; majority of panel members thought it would be large.<br><br>Panel members agreed that the direct **cost of OIT with baked milk would be similar to OIT with fresh milk** and that the determinants of the cost would be the same.

Equity

What would be the impact on health equity?

JUDGEMENT	RESEARCH EVIDENCE	ADDITIONAL CONSIDERATIONS
○ Reduced<br>○ Probably reduced<br>○ Probably no impact<br>○ Probably increased<br>○ Increased<br>● Varies<br>○ Don't know	No research evidence was found.	**OIT is currently not reimbursed in many countries** and many third-party health insurance systems do not cover it, so **it would be available only to more affluent families** that could cover the cost out of the pocket or through a more expensive health insurance.<br>Cost and availability of specialized facilities to perform OIT are more likely to limit implementation in jurisdictions where fewer resources are available.<br>Panel members thought that the impact on health equity would vary depending on who bears the cost of OIT (patients and families themselves, public health care system, or private third party payers) and whether or not all or only selected more expensive insurance systems would cover OIT.

Acceptability

Is the intervention acceptable to key stakeholders?

JUDGEMENT	RESEARCH EVIDENCE	ADDITIONAL CONSIDERATIONS
○ No<br>○ Probably no<br>○ Probably yes<br>○ Yes<br>● Varies<br>○ Don't know	No research evidence was found.	The **autonomy of patients** (e.g. not doing physical exercises after OIT) as well as parents (e.g. need to supervise OIT and intervene if necessary) may be affected.<br>**Clinicians as well as family members may vary** in their perception of risk and the relative value they place on avoiding reactions with accidental exposure to milk or with OIT. Thus, some clinicians and family members may be reluctant to administer or accept OIT while others will not. Some **clinicians** may not accept the risk of allergic reactions that occur during OIT in their offices.

		**Preschool and school personnel** may not accept providing and/or supervising milk OIT during school trips.<br>**Third party payers** may not accept the additional cost of specialized health care personnel and clinical facilities required for OIT.<br>The long-term effects and persistence of desensitization are still being investigated. This uncertainty influences the variability in acceptance of OIT by patients, their families, clinicians, and third party payers. The general perception of importance of food allergy varies across cultures and also affects the acceptability of related interventions.

Feasibility
Is the intervention feasible to implement?

JUDGEMENT	RESEARCH EVIDENCE	ADDITIONAL CONSIDERATIONS
○ No<br>○ Probably no<br>● Probably yes<br>○ Yes<br>○ Varies<br>○ Don't know	No research evidence was found.	Panel members found the following to be currently the **main barriers to implementation:**<br>- **additional cost** of OIT<br>- **limited availability** and access to OIT in many countries<br>- limited and **variable availability of facilities** for OIT<br>- **limited availability of allergy specialists** who would have to provide and supervise OIT<br>- **need for education and training for patients** and their families<br>- need to start OIT in a hospital (in settings in which it is required).<br>School personnel is unlikely to provide or supervise OIT if it needs to be done on a school trip. Parents or other caregivers would have to accompany children on those occasions.<br>**Lifelong or long-term OIT may not be sustainable** owing to its cumulative cost and burden.<br>The **inappropriate use** of milk OIT would increase the risk of serious adverse effects in children with severe food allergies. However, it would be unlikely if it was used in patients correctly diagnosed with IgE-mediated CMA and properly administered by allergy specialists.

SUMMARY OF JUDGEMENTS

	JUDGEMENT						
**DESIRABLE EFFECTS**	Trivial	**Small**	Moderate	Large	–	Varies	Don't know
**UNDESIRABLE EFFECTS**	Large	**Moderate**	Small	Trivial	–	Varies	Don't know
**CERTAINTY OF EVIDENCE**	**Very low**	Low	Moderate	High	–	–	No included studies
**VALUES**	Important uncertainty or variability	**Possibly important uncertainty or variability**	Probably no important uncertainty or variability	No important uncertainty or variability	–	–	–

	JUDGEMENT						
**BALANCE OF EFFECTS**	Favors the comparison	**Probably favors the comparison**	Does not favor either the intervention or the comparison	Probably favors the intervention	Favors the intervention	Varies	Don't know
**RESOURCES REQUIRED**	**Large costs**	Moderate costs	Negligible costs and savings	Moderate savings	Large savings	Varies	Don't know
**EQUITY**	Reduced	Probably reduced	Probably no impact	Probably increased	Increased	**Varies**	Don't know
**ACCEPTABILITY**	No	Probably no	Probably yes	Yes	–	**Varies**	Don't know
**FEASIBILITY**	No	Probably no	**Probably yes**	Yes	–	Varies	Don't know

TYPE OF RECOMMENDATION

Strong recommendation against the intervention	Conditional recommendation against the intervention	Conditional recommendation for either the intervention or the comparison	Conditional recommendation for the intervention	Strong recommendation for the intervention
○	●	○	○	○

CONCLUSIONS

Recommendation

In people with IgE-mediated CMA who do not tolerate baked milk, we suggest that clinicians do not use oral immunotherapy with baked cow's milk.
(CONDITIONAL recommendation based on very low certainty evidence)

Remark: Persons with IgE-mediated CMA who do tolerate specific amounts of baked cow's milk can continue consuming it.

Justification

Balance of the desirable and undesirable effects is unclear because of the very low certainty of the evidence. Panel members agreed that more and higher quality evidence would be desirable to obtain and once available, it is likely to influence the strength but also the direction of this recommendation.
Panel members thought that in any case the balance will depend on patient's and family's values and preferences.

Subgroup considerations

No specific subgroups were identified.

Implementation considerations

Diagnosis of IgE-mediated CMA with reactions to baked milk must be confirmed before commencing OIT with baked milk.
When choosing to preform OIT, clinicians may want to consider the following situations that may be contraindications for starting and for continuation of OIT:
- a patient and her/his family is not able to follow the protocol
- a patient and family has no access to epinephrine and/or is not able to properly use it when needed
- a physician managing OIT is not able to devote sufficient time and resources to properly administer and monitor OIT.
- a patient has a history of confirmed previous frequent severe reactions to baked cow's milk
- a child has persistent gastrointestinal symptoms

- a patient has a concomitant asthma that is not well controlled.

Monitoring and evaluation

When choosing to preform OIT with baked milk, clinicians need to monitor symptoms in all patients and proper nutrition in small children.

Research priorities

1. Temperature and time of heating/baking cow's milk products.
2. The effects of OIT with baked milk on quality of life of patients and their family members.
3. Costs of OIT with baked milk.

Question: Should oral immunotherapy with cow's milk vs. elimination diet alone be used for IgE-mediated cow's milk allergy?

Certainty assessment						№ of patients		Effect		Certainty	Importance
№ of studies	Risk of bias	Inconsistency	Indirectness	Imprecision	Other considerations	OIT with cow's milk	Elimination diet only	Relative (95% CI)	Absolute (95% CI)		
**Anaphylaxis**											
7 RCT [1,2,3,4,5,6,7]<br>follow-up: 6 to 17 months	not serious [a]	serious [b]	not serious	not serious [c]	none	Only one study (De Schryver 2019) used current definition of anaphylaxis. The rate of anaphylaxis in this study was 1 per 100 persons per year <u>without</u> OIT and 550 per 100 persons per year <u>with</u> OIT (rate ratio: 60.0; 95% CI: 15 to 244; rate difference: 5 more per 1 person per year (95% CI: 4 to 6)). One study (Skripak 2008) defined anaphylaxis as "some combination of respiratory, gastrointestinal, and/or skin reaction" and reported similar results. Four additional studies reported no anaphylactic reactions, however, they either did not provide the definition that they used or equated it with epinephrine use.				⊕⊕⊕◯<br>MODERATE	CRITICAL
**14** NRS<br>follow-up: median 2 years<br>(338 person-years)	serious [A]	serious [B]	serious [C]	not serious	none	At least 1 event: **8.9%** of patients receiving OIT (95% CI: 4.3 to 13.5) (12 studies)<br>Incidence rate: **44 per 100 patients per year** (95% CI: 2 to 869) (5 studies)				⊕◯◯◯<br>VERY LOW	
**Use of IM epinephrine**											
7 RCT [1,3,4,5,7,8]<br>follow-up: 4 to 12 months	not serious [d]	not serious	not serious	not serious	none	85/134	2/120	IRR 29.4<br>(7.4 to 117.0)	**268 more per 100 patients per year**<br>(from 203 more to 333 more) [e]	⊕⊕⊕⊕<br>HIGH	CRITICAL
**28** NRS<br>follow-up: median 1 year<br>(1604 person-years)	serious [A]	serious [D]	not serious	not serious	none	At least 1 event during up-dosing phase of OIT: **15%** of patients (95% CI: 10 to 20) (17 studies)<br>At least 1 event during maintenance phase: **5%** of patients (95% CI: 3 to 8) (15 studies)<br>Incidence rate: **16 per 100 patients per year** (95% CI: 6 to 38) (18 studies).				⊕⊕◯◯<br>LOW	
**Adverse effect leading to the discontinuation of treatment**											
7 RCT [1,2,3,4,5,6,7]	not serious [d]	not serious	not serious	serious [f]	none	20/146 (13.7%)	9/132 (6.8%)	RR 1.8<br>(0.84 to 3.84)	**5 more per 100**<br>(from 1 fewer to 19 more) [g]	⊕⊕⊕◯<br>MODERATE	CRITICAL
**16** NRS	not serious	serious [E]	not serious	not serious	none	**12%** (95% CI: 8 to 16). 89/768 patients starting OIT.				⊕⊕⊕◯<br>MODERATE	
**Severe gastrointestinal symptoms**											
5 RCT [1,2,4,5,7]<br>follow-up: 4 to 17 months	not serious [d]	not serious	serious [h]	serious [i]	none	45/91 (49.5%)	4/84 (4.8%)	RR 6.9<br>(1.6 to 30.9) [j]	**28 more per 100**<br>(from 3 more to 100 more)	⊕⊕◯◯<br>LOW	CRITICAL
**10** NRS<br>follow-up: median 1.5 year<br>(277 person-years)	serious [A]	serious	serious [F]	not serious	none	At least 1 event: **31%** of patients receiving OIT (95% CI: 14 to 49) (7 studies)<br>Incidence rate: **258 per 100 patients per year** (95% CI: 39 to 1698) (4 studies)				⊕◯◯◯<br>VERY LOW	
**Severe respiratory symptoms/wheezing**											
1 RCT [4]<br>follow-up: 12 months<br>assessed with: "nebulized epinephrine for respiratory symptoms"	not serious [d]	not serious	not serious	serious [k]	none	24/30 (80.0%)	0/30 (0.0%)	RR 49.0<br>(3.12 to 770.59)	**77 more per 100**<br>(from 62 more to 92 more) [e]	⊕⊕⊕◯<br>MODERATE	CRITICAL
7 NRS<br>follow-up: median 2 years<br>(246 person-years)	serious [A]	not serious	serious [F]	not serious	none	At least 1 event: **9%** of patients receiving OIT (95% CI: 4 to 14) (2 studies). Additional 4 studies reported wheezing but did not specify its severity: 17% (95% CI: 8 to 27).<br>Incidence rate: **916 per 100 patients per year** (95% CI: 626 to 1339) (3 studies)				⊕⊕◯◯<br>LOW	
**Generalized erythema, urticaria and angioedema?**											
5 RCT [1,2,4,5,7]<br>follow-up: 4 to 17 months	not serious [d,l]	not serious	not serious	serious [m]	none	27/89 (30.3%)	7/82 (8.5%)	RR 2.8<br>(0.74 to 10.36) [n]	**15 more per 100**<br>(from 2 fewer to 80 more)	⊕⊕⊕◯<br>MODERATE	CRITICAL
7 NRS	serious [A]	not serious	serious [G]	not serious	none	At least 1 event: **40%** of patients receiving OIT (95% CI: 1 to 78) (4 studies)<br>Incidence rate: **178 per 100 patients per year** (95% CI: 59 to 541) (5 studies)				⊕⊕◯◯<br>LOW	

follow-up: median 1 year (291 person-years)											
**Ability to drink cow's milk and eat dairy products without a reaction**											
**7** RCT [1-8]<br>follow-up: 4 to 11 months<br>assessed with: passing a graded food challenge with ≥150 ml cow's milk and/or ability to drink cow's milk and eat dairy products without symptoms	not serious [p]	not serious	very serious [q]	not serious [r]	none [s,t]	100/144 (69.4%)	3/121 (2.5%)	RR 12.1<br>(5.59 to 26.21) [u]	**28 more per 100**<br>(from 11 more to 63 more)	⊕⊕⊕◯<br>MODERATE	CRITICAL
**Ability to accidentally consume a small amount of cow's milk without a reaction**											
**7** RCT [1,2,3,4,5,7,8]<br>follow-up: range 4 months to 11 months<br>assessed with: passing a supervised graded food challenge with ≥5 ml of cow's milk	not serious [p]	not serious	very serious [q]	not serious	none [s,t]	100/123 (81.3%)	4/108 (3.7%) [af]<br>13.0% [af]	RR 10.4<br>(5.12 to 21.11)	**35 more per 100**<br>(from 15 more to 74 more) [af]<br>**100 more per 100**<br>(from 54 more to 100 more) [af]	⊕⊕⊕◯<br>MODERATE	CRITICAL
**Tolerance of cow's milk when it is reintroduced after a period of not consuming milk and milk products**											
0 RCT (not measured)	-	-	-	-	-	-	-	-	-	-	CRITICAL
**8** NRS<br>follow-up: median 1 year<br>assessed with passing graded OFC after 2-8 weeks of elimination diet	serious [A]	serious [J]	serious [K]	not serious [L]	none	**44%** patients starting OIT (95% CI: 18 to 69).				⊕◯◯◯<br>VERY LOW	
**Emergency department visit**											
**2** RCT [2,4]	serious [v]	not serious	not serious	very serious [w]	none	There were 2 events among 48 children in OIT group and no events in control groups. Only 2 of the 11 studies reported this outcome.				⊕◯◯◯<br>VERY LOW	CRITICAL
**4** NRS<br>follow-up: median	serious [A]	not serious	not serious	serious [H]	none	**1.8%** of patients receiving OIT visited ED at least once (95% CI: 0 to 3.7) (4 studies)				⊕⊕◯◯<br>LOW	
**Hospital admission**											
**0** RCT (not reported)	-	-	-	-	-	-	-	-	-	-	IMPORTANT
**6** NRS<br>follow-up: median	serious [A]	not serious	not serious	serious	none	There were no hospitalizations among 264 patients in 6 studies of OIT that reported this outcome.				⊕⊕◯◯<br>LOW	
**Death**											
**7** RCT [1-5,9,10]<br>follow-up: 4 to 11 months	not serious	not serious	not serious	not serious	none	0/129 (0.0%)	0/103 (0.0%)	not estimable	**0 fewer per 100**<br>(from 4 fewer to 3 more) [e]	⊕⊕⊕⊕<br>HIGH	IMPORTANT
**0** NRS (not reported)	-	-	-	-	-	-	-	-	-	-	
**Mild respiratory symptoms**											
**5** RCT [1,4,5,7,10]<br>follow-up: 16 to 40 weeks	not serious [d,l]	not serious	not serious	serious [y]	none	20/73 (27.4%)	1/74 (1.4%)	RR 10.0<br>(2.41 to 41.43) [z]	**12 more per 100**<br>(from 2 more to 55 more)	⊕⊕⊕◯<br>MODERATE	IMPORTANT
**7** NRS<br>follow-up: median 2 years (246 person-years)	serious [A]	not serious	serious [F]	not serious	none	**16%** (95% CI: 8 to 25) of patients receiving OIT (Studies did not report severity of symptoms) (5 studies)<br>Incidence rate: **916 per 100 patients per year** (95% CI: 626 to 1339) (3 studies)				⊕⊕◯◯<br>LOW	
**Angioedema**											

**4** RCT [1,2,4,7]	not serious [d,l]	not serious	not serious	serious [o]	none	8/59 (13.6%)	0/52 (0.0%)	RR 4.7 (0.85 to 25.82)	**12 more per 100** (from 2 more to 22 more) [e]	⊕⊕⊕◯ MODERATE	IMPORTANT
**1** NRS follow-up: 2 years	serious [A]	not serious	not serious	very serious	none	1 event among 21 patients receiving OIT (5%)				⊕◯◯◯ VERY LOW	
**Lip or mouth pruritus, perioral rash**											
**5** RCT [1,2,3,4,5]	serious [l]	not serious	not serious	serious [ab]	none	47/76 (61.8%)	1/68 (1.5%)	RR 12.8 (2.5 to 65.4) [ac,ad]	17 more per 100 (from 2 more to 95 more)	⊕⊕◯◯ LOW	IMPORTANT
**9** NRS follow-up: median 1 year (298 patient-years)	serious [A]	serious	not serious	not serious	none	At least 1 event: **48%** patients receiving OIT (95% CI: 18 to 78) (5 studies)<br>Incidence rate: **990 per 100 patients per year** (95% CI: 249 to 3929) (5 studies)				⊕⊕◯◯ LOW	
**Eosinophilic esophagitis**											
**0** RCT (not reported)	-	-	-	-	-	-	-	-	-	-	IMPORTANT
**6** NRS follow-up: 2 years	serious [A]	not serious	serious [l]	serious	none	**9%** of patients receiving OIT (81/877) developed EoE at least once (95% CI: 4 to 15).				⊕◯◯◯ VERY LOW	
**Quality of life of children**											
**0** RCT (not reported)	-	-	-	-	-	-	-	-	-	-	IMPORTANT
**1** NRS follow-up: 5 weeks assessed with food allergy quality of life questionnaire - parent form (FAQLQ-PF); MID: 0.5 point	serious [A]	not serious	serious [M]	serious [N]	none	FAQLQ-PF: in 21 patients QoL deteriorated, in 31 remained unchanged, and in 30 was improved.				⊕◯◯◯ VERY LOW	
**Quality of life of the caregivers**											
**0** RCT (not reported)	-	-	-	-	-	-	-	-	-	-	IMPORTANT
**0** NRS (not reported)	-	-	-	-	-	-	-	-	-	-	
**Any adverse effect**											
**6** RCT [1,2,3,4,5,7]	not serious [d]	not serious	serious [x]	not serious	none	100/116 (86.2%)	20/100 (17.0%)	RR 3.63 (1.73 to 7.61)	57 more per 100 (from 7 more to 100 more)	⊕⊕⊕◯ MODERATE	IMPORTANT
**22** NRS follow-up: median 1.5 years (1158 patient-years)	serious [A]	not serious [O]	very serious [P]	not serious	none	At least 1 event: **53%** patients receiving OIT (95% CI: 29 to 77) (14 studies)<br>Incidence rate: **564 per 100 patients per year** (95% CI: 172 to 1848) (14 studies)				⊕◯◯◯ VERY LOW	

CI: Confidence interval; **IRR:** incidence rate ratio; **MD:** Mean difference; **MID:** minimal important difference; **NRS:** non-randomized (observational) study; **OFC:** oral food challenge; **RCT:** Randomized controlled trial; **RR:** Risk ratio

Explanations

a. The only study (De Schryver 2019) that reported direct evidence was not blinded and was stopped early because of apparent benefit. However, both of those biases are likely to underestimate adverse effects.
b. 2 studies reported the rate of anaphylaxis with OIT between 4.7 and 5.5 per person per year and other 4 studies reported no anaphylactic reactions with OIT. This difference could not be explained with population characteristics or the type of OIT. It is possible that the difference is related to the definition of anaphylaxis used in individual studies; 3 studies did not report what definition was used. We did not reduce the certainty because of indirectness (one study provided a direct outcome measure) but rather because of inconsistency, as they seem to be related.
c. We did not lower the certainty because of imprecision, because the results of one study that could be used provided precise estimates. If data from other studies could be used then the judgment about precision might change.
d. Most studies were not blinded but it is unlikely that this would overestimate the risk of adverse effects.
e. There were no events in control groups; 95% CI around the risk difference was estimated from risk difference meta-analysis.
f. Only 29 events; confidence interval does not exclude an appreciable harm with OIT or no difference.
g. Sensitivity analysis assuming 0 events among controls in De Schryver 2019 - RD: 12 more per 100.
h. Studies did not report GI symptoms consistently – some may have had very different importance for patients than the others.
i. The CI does not exclude an appreciably increased risk of GI symptoms or no difference.
j. In 2 studies the rate of reactions per patient was reported. Across these studies the rate of GI symptoms was 21.4 times higher (95% CI: 8.9 to 51.8) with OIT than without.
k. Only 24 events; 95% confidence interval does not exclude an appreciable benefit or an appreciable harm.
l. Few studies reported this outcome that we considered obvious to measure and report; in general, adverse effects were reported inconsistently, using variable definitions, and sometimes precluding meaningful conclusions.
m. There were only 34 events and the pooled confidence interval does not exclude harm from OIT or no difference.
n. Two additional studies measured urticaria as rate of events per patient. Rate of generalized urticaria was 8.3 times higher (95% CI: 3.2 to 21.1) with OIT than without.

o. Only 8 events; CI does not exclude an appreciable harm with OIT or no effect
p. In some studies participants were not blinded but the results were consistent across all studies. Although the true effect might be smaller than the presented estimate, we did not rate down the certainty of evidence for risk of bias given the very strong association.
q. It is not certain whether all those passing a graded food challenge in a clinic will also be able to tolerate an equivalent total amount of milk without a graded challenge.
r. Total 85 events among 217 patients
s. There is some suggestion of publication bias as all studies were small and all showed very large effect. We did not reduce the certainty of evidence because we already reduced it for indirectness.
t. There was a very large association that does increase the confidence in the estimated effect on an indirect outcome. However, because of this indirectness we thought that very strong association may not apply to the outcome of interest.
u. One additional study (Morisset 2007) explicitly included only children that could tolerate at least 60 ml of milk at baseline and found a smaller effect of OIT RR: 1.44 (95% CI: 0.98 to 2.11). Another RCT published as a conference abstract only reported tolerance in 4/11 children receiving OIT but did not report how many children achieved tolerance in control group (n = 4) (Filho 2015).
v. Only 2 of 7 studies reported this outcome.
w. Only 2 events in one study.
x. There were many adverse effects with various importance to patients.
y. There were only 21 events and the pooled confidence interval does not exclude harm from OIT or no difference.
z. In 2 studies the rate of reactions per patient was reported. Across these studies the rate of asthma/wheezing was 11 times higher (95% CI: 0.97 to 125.0) with OIT than without.
aa. Only 16 events in one group.
ab. Only 50 events. CI does not exclude an appreciable harm or little difference.
ac. Three Additional studies show small to large increase In a number of reactions per patient in the OIT group compared to controls. The results were not consistent therefore we did not combine them in meta-analysis. The rates ratios individual studies were 880.07, 713.37, and 4.53.
ad. One study (Lee 2013) was excluded from the analysis as ii failed to report data on the control group
A. No control group (series of cases). Any inference requires implicit comparison.
B. Rate of anaphylaxis varied between none to 46%. We could not explain it with the characteristics of the population or the type of OIT protocol.
C. Most studies did not report how they defined anaphylaxis. Based on the variability of definitions used in RCTs we assumed that they would also be variable in observational studies and not reflecting the current definition.
D. Use of epinephrine IM varied between none to 60% of patients. We could not explain it with the characteristics of the population or the type of OIT protocol.
E. Discontinuation varied between 3% and 40%. We could not explain it with the characteristics of the population or the type of OIT protocol.
F. We pooled data of any respiratory symptom unless it was specified to be severe. Most studies did not report the severity of symptoms.
G. Most studies reported urticaria without mentioning its range or severity.
H. Only 6 events among 230 patients.
I. EoE was not confirmed with biopsy in most studies.
J. Proportion of children being able to tolerate milk varied between 20% and 91%. We were not able to explain it with the duration and target dose of OIT, duration of avoidance diet, and the dose of milk in OFC.
K. It is not certain whether all those passing a graded food challenge in a clinic will also be able to tolerate an equivalent total amount of milk without a graded challenge. It is also uncertain whether 2-8 weeks of strict avoidance of milk would have similar effects as a longer period of usual uncontrolled diet.
L. The results were not precise, but we assumed that this is because of inconsistency and we did not reduce the already very low certainty for imprecision.
M. Outcome was measured during the initial phase of OIT, whereas QoL of children during the whole period of treatment and after OIT is of interest.
N. Only 82 patients.
O. Proportion of adverse effects was very inconsistent among studies, but we assumed that this was owing to their varying definitions and reporting.
P. There were many different adverse effects with different importance to patients. They were also inconsistently defined and reported among the studies.

References

References to randomized trails:

1. Martorell A, De la Hoz B,Ibáñez MD,Bone J,Terrados MS,Michavila A,Plaza AM,Alonso E,Garde J,Nevot S,Echeverria L,Santana C,Cerdá JC,Escudero C,Guallar I,Piquer M,Zapatero L,Ferré L,Bracamonte T,Muriel A,Martínez MI,Félix R.. Oral desensitization as a useful treatment in 2-year-old children with cow's milk allergy. Clin Exp Allergy; 2011.
2. Salmivesi S, Korppi M,Mäkelä MJ,Paassilta M.. Milk oral immunotherapy is effective in school-aged children. Acta Paediatr; 2013.
3. Lee JH, Kim WS, Kim H,Hahn YS. Increased cow's milk proteinespecific IgG4 levels after oral desensitization in 7- to 12-month-old infants. Ann Allergy Asthma Immunol; 2013.
4. Longo G, Barbi E, Berti I,Meneghetti R,Pittalis A,Ronfani L,Ventura A.. Specific oral tolerance induction in children with very severe cow's milk-induced reactions. J Allergy Clin Immunol; 2008.
5. Skripak JM, Nash SD,Rowley H,Brereton NH,Oh S,et al.. A randomized, double-blind, placebo-controlled study of milk oral immunotherapy for cow's milk allergy. J Allergy Clin Immunol; 2008.
6. De Schryver, Sarah. Adverse Events in Oral Immunotherapy for the Desensitization of Cow's Milk Allergy in Children: A Randomized Controlled Trial. J Allergy Clin Immunol Pract; 2019.
7. Pajno GB, Caminiti L,Ruggeri P,De Luca R,Vita D,La Rosa M,Passalacqua G.. Oral immunotherapy for cow's milk allergy with a weekly up-dosing regimen: a randomized single-blind controlled study.. Ann Allergy Asthma Immunol; 2010.
8. Patriarca, G., Schiavino, D., Nucera, E., Schinco, G., Milani, A., Gasbarrini, G. B.. Food allergy in children: results of a standardized protocol for oral desensitization. Hepato-Gastroenterology; 1998.
9. FIlho WR, Flausino FJ,Silva RA,Silva FM,Pinto JA,Nunes L. Risks and benefits of oral immunotherapy for IgE-mediated cow's milk allergy. World Allergy Organization Journal; 2015.
10. Morisset M, Moneret-Vautrin DA,Guenard L,Cuny JM,Frentz P,et al. Oral desensitization in children with milk and egg allergies obtains recovery in a significant proportion of cases. A randomized study in 60 children with cow's milk allergy and 90 children with egg allergy. Eur Ann Allergy Clin Immunol; 2007.

References to observational studies:

1. Alvaro M, Giner MT, Vazquez M, Lozano J, Dominguez O, Piquer M, et al. Specific oral desensitization in children with IgE-mediated cow's milk allergy. Evolution in one year. Eur J Pediatr 2012; 171:1389-95.
2. Alves-Correia M, Gaspar A, Borrego LM, Azevedo J, Martins C, Morais-Almeida M. Successful oral desensitization in children with cow's milk anaphylaxis: Clinical and laboratory evaluation up to nine-years follow-up. Allergol Immunopathol (Madr) 2019; 47:133-40.
3. Aquilante BP, Nhola Faion MC, Veiga FMS, Soldateli G, Yonamine GH, Santos CN, et al. Eosinophilic esophagitis during milk oral immunotherapy: a growing concern. Journal of Allergy and Clinical Immunology 2018; 141:AB135.
4. Babaie D, Nabavi M, Arshi S, Mesdaghi M, Chavoshzadeh Z, Bemanian MH, et al. Cow's Milk Desensitization in Anaphylactic Patients: A New Personalized-dose Method. Iran J Allergy Asthma Immunol 2017; 16:45-52.
5. Bellón S, Sánchez L, González L, Moreira A, Bracamonte T, Quevedo S, et al. Evolution of immunologic parameters in children subjected to food oral immunotherapy with cow's milk during a 10-year follow-up. Clinical and Translational Allergy 2018; 8:26.
6. Bellón S, Sánchez L, Muñoz C, Bracamonte T, Fernández S, Quevedo S, et al. High prevalence of primary eosinophilic gastrointestinal disorders in children who have received food oral immunotherapy. Clinical and Translational Allergy 2018; 8:O20.
7. Bellón S, Sánchez L, González L, Moreira A, Bracamonte T, Quevedo S, et al. Cow's milk protein consumption and risk of adverse reactions in children subjected to food oral immunotherapy with cow's milk during a long-term follow-up. Clinical and Translational Allergy 2018:O2.

8. Berti I, Badina L, Cozzi G, Giangreco M, Bibalo C, Ronfani L, et al. Early oral immunotherapy in infants with cow's milk protein allergy. Pediatric Allergy & Immunology 2019; 30:572-4.
9. De Schryver S, Mazer B, Clarke AE, St Pierre Y, Lejtenyi D, Langlois A, et al. Adverse Events in Oral Immunotherapy for the Desensitization of Cow's Milk Allergy in Children: A Randomized Controlled Trial. J Allergy Clin Immunol Pract 2019; 7:1912-9.
10. Ebrahimi M, Gharagozlou M, Khalili A, Magaji Hamid K, Azizi G, Movahedi M. Induction of Tolerance by Oral Immunotherapy in Patients With Cow's Milk Allergy. Journal of Investigational Allergology & Clinical Immunology 2016; 26:341-3.
11. Ebrahimi M, Gharagozlou M, Mohebbi A, Hafezi N, Azizi G, Movahedi M. The Efficacy of Oral Immunotherapy in Patients with Cow's Milk Allergy. Iran J Allergy Asthma Immunol 2017; 16:183-92.
12. ElBadawy NE, Abdel-Latif RS. Food Specific IgE as A Biomarker of Oral Immunotherapy Efficacy in Comparison to Double Blind Food Challenge Test. Egypt J Immunol 2017; 24:109-25.
13. Elizur A, Goldberg MR, Levy MB, Nachshon L, Katz Y. Oral immunotherapy in cow's milk allergic patients: course and long-term outcome according to asthma status. Annals of Allergy, Asthma, & Immunology 2015; 114:240-4.e1.
14. Elizur A, Appel MY, Goldberg MR, Yichie T, Levy MB, Nachshon L, et al. Clinical and laboratory 2-year outcome of oral immunotherapy in patients with cow's milk allergy. Allergy 2016; 71:275-8.
15. Epstein-Rigbi N, Goldberg MR, Levy MB, Nachshon L, Elizur A. Changes in quality of life of food-allergic children from initiation of oral immunotherapy, through updosing, upon reaching maintenance and after 6 months of follow-up. Journal of Allergy and Clinical Immunology 2018; Conference:2018 American Academy of Allergy, Asthma and Immunology, AAAAI and World Allergy Organization, WAO Joint Congress. United States. 141 (2 Supplement 1) (pp AB240).
16. Frischmeyer-Guerrerio PA, Masilamani M, Gu W, Brittain E, Wood R, Kim J, et al. Mechanistic correlates of clinical responses to omalizumab in the setting of oral immunotherapy for milk allergy. Journal of Allergy & Clinical Immunology 2017; 140:1043-53.e8.
17. Garcia-Ara C, Pedrosa M, Belver MT, Martin-Munoz MF, Quirce S, Boyano-Martinez T. Efficacy and safety of oral desensitization in children with cow's milk allergy according to their serum specific IgE level. Ann Allergy Asthma Immunol 2013; 110:290-4.
18. Garcia-Lirio E, Gonzalez Diaz C, Gonzalez Hermosa A, Gamboa P, Aranguren R, Sanz ML. Oral Immunotherapy With Egg and Milk: Changes in Peripheral Serum Cytokines Are Not Predictive Factors for Severe Adverse Reactions or for the Final Report. Journal of Investigational Allergology & Clinical Immunology 2018; 28:24-8.
19. Goldberg MR, Elizur A, Nachshon L, Appel MY, Levy MB, Golobov K, et al. Oral immunotherapy-induced gastrointestinal symptoms and peripheral blood eosinophil responses. J Allergy Clin Immunol 2017; 139:1388-90 e4.
20. Goldberg MR, Nachshon L, Levy MB, Elizur A, Katz Y. Risk Factors and Treatment Outcomes for Oral Immunotherapy-Induced Gastrointestinal Symptoms and Eosinophilic Responses (OITIGER). The Journal of Allergy & Clinical Immunology in Practice 2019; 02:02.
21. Gonzalez Jimenez D, Larrea Tamayo E, Diaz Martin JJ, Molinos Norniella C, Perez Solis D, Menendez Arias C, et al. [Oral rush desensitization for cow milk allergy: Clinical and immunological follow-up]. An Pediatr (Barc) 2013; 79:346-51.
22. Kaneko H, Teramoto T, Kondo M, Morita H, Ohnishi H, Orii K, et al. Efficacy of the slow dose-up method for specific oral tolerance induction in children with cow's milk allergy: comparison with reported protocols. Journal of Investigational Allergology & Clinical Immunology 2010; 20:538-9.
23. Kauppi P, Kukkonen AK, Hakulinen A, Helin T, Voutilainen H, Pelkonen A, et al. Oral immunotherapy in severe milk, peanut or egg allergy in adults - preliminary results. Allergy; 69:526.
24. Kim JS, Wood RA, Lindblad R, Noone SA, Paterakis MN, Henning A. A randomized, double-blind, placebo-controlled trial of omalizumab combined with oral immunotherapy (OIT) in the treatment of cow's milk allergy (CMA): safety of dosing. Journal of allergy and clinical immunology 2014; 133:AB403.
25. Kivisto JE, Korppi M, Helminen M, Maki T, Paassilta M. Half of the children who received oral immunotherapy for a cows' milk allergy consumed milk freely after 2.5 years. Acta Paediatr 2015; 104:1164-8.
26. Kuitunen M, Englund H, Remes S, Moverare R, Pelkonen A, Borres MP, et al. High IgE levels to alpha-lactalbumin, beta-lactoglobulin and casein predict less successful cow's milk oral immunotherapy. Allergy 2015; 70:955-62.
27. Levy MB, Elizur A, Goldberg MR, Nachshon L, Katz Y. Clinical predictors for favorable outcomes in an oral immunotherapy program for IgE-mediated cow's milk allergy. Ann Allergy Asthma Immunol 2014; 112:58-63 e1.
28. Longo G, Berti I, Barbi E, Calligaris L, Matarazzo L, Radillo O, et al. Diagnosed child, treated child: food challenge as the first step toward tolerance induction in cow's milk protein allergy. Eur Ann Allergy Clin Immunol 2012; 44:54-60.
29. Luyt D, Bravin K, Luyt J. Implementing specific oral tolerance induction to milk into routine clinical practice: experience from first 50 patients. J Asthma Allergy 2014; 7:1-9.
30. Mantyla J, Thomander T, Hakulinen A, Kukkonen K, Palosuo K, Voutilainen H, et al. The effect of oral immunotherapy treatment in severe IgE mediated milk, peanut, and egg allergy in adults. Immun Inflamm Dis 2018; 6:307-11.
31. Martinez-Botas J, Rodriguez-Alvarez M, Cerecedo I, Vlaicu C, Dieguez MC, Gomez-Coronado D, et al. Identification of novel peptide biomarkers to predict safety and efficacy of cow's milk oral immunotherapy by peptide microarray. Clinical & Experimental Allergy 2015; 45:1071-84.
32. Meglio P, Bartone E, Plantamura M, Arabito E, Giampietro PG. A protocol for oral desensitization in children with IgE-mediated cow's milk allergy. Allergy 2004; 59:980-7.
33. Meglio P, Giampietro PG, Gianni S, Galli E. Oral desensitization in children with immunoglobulin E-mediated cow's milk allergy--follow-up at 4 yr and 8 months. Pediatric Allergy & Immunology 2008; 19:412-9.
34. Miura Y, Nagakura K, Yanagida N, Sato S, Ebisawa M. Efficacy and safety of low-dose oral immunotherapy for patients with severe cow's milk allergies: A 3-year follow-up. Allergy: European Journal of Allergy and Clinical Immunology 2018; Conference:37th Annual Congress of the European Academy of Allergy and Clinical Immunology, EAACI 2018. Germany. 73 (Supplement 105) (pp 54).
35. Nct. Milk Oral Immunotherapy in Children to Treat Food Allergy. Https://clinicaltrials.gov/show/nct01162473 2010.
36. Ogura K, Imai T, Iikura K, Sato S, Komata T, Tomikawa M, et al. Variable efficacy of oral immunotherapy for hen's egg, cow's milk and wheat allergy: compared to their natural courses. Allergy 2012; 67:412.
37. Ogura K, Komata T, Hasegawa M, Hayashi N, Satou M, Goto M, et al. Efficacy of Slow Oral Immunotherapy for Cow's Milk Allergy. The World Allergy Organization Journal 2012; 5:S41-S2.
38. Ono M, Takasato Y, Matsui T, Sugiura S, Ito K. The efficacy and safety of tailored oral immunotherapy for patients with mild food allergies. Allergy: European Journal of Allergy and Clinical Immunology 2018; Conference:37th Annual Congress of the European Academy of Allergy and Clinical Immunology, EAACI 2018. Germany. 73 (Supplement 105) (pp 321).
39. Paassilta M, Salmivesi S, Maki T, Helminen M, Korppi M. Children who were treated with oral immunotherapy for cows' milk allergy showed long-term desensitisation seven years later. Acta Paediatrica 2016; 105:215-9.
40. Patriarca G, Buonomo A, Roncallo C, Del Ninno M, Pollastrini E, Milani A, et al. Oral desensitisation in cow milk allergy: immunological findings. Int J Immunopathol Pharmacol 2002; 15:53-8.
41. Patriarca G, Nucera E, Pollastrini E, Roncallo C, De Pasquale T, Lombardo C, et al. Oral specific desensitization in food-allergic children. Digestive Diseases & Sciences 2007; 52:1662-72.
42. Perezabad L, Reche M, Valbuena T, Lopez-Fandino R, Molina E, Lopez-Exposito I. Oral Food Desensitization in Children With IgE-Mediated Cow's Milk Allergy: Immunological Changes Underlying Desensitization. Allergy Asthma Immunol Res 2017; 9:35-42.
43. Reche M, Valbuena T, Fiandor A, Padial A, Cañete A, Pascual C. Early Induction Of Oral Tolerance Protocol (OTI) In Children With Cow'S Milk Allergy. Journal of Allergy and Clinical Immunology 2011; 127:AB24.
44. Sato S, Yanagida N, Ebisawa M. Initial Antigen-Specific IgE Levels Predict Clinical Outcome of Rush Oral Immunotherapy for Food Anaphylaxis. World Allergy Organization Journal 2019; Conference:XXIV World Allergy Congress 2015. South Korea. 18 (2) (pp 93-94).
45. Savilahti EM, Kuitunen M, Savilahti E, Makela MJ. Specific antibodies in oral immunotherapy for cow's milk allergy: kinetics and prediction of clinical outcome. International Archives of Allergy & Immunology 2014; 164:32-9.
46. Savilahti EM, Kuitunen M, Valori M, Rantanen V, Bardina L, Gimenez G, et al. Use of IgE and IgG4 epitope binding to predict the outcome of oral immunotherapy in cow's milk allergy. Pediatric Allergy & Immunology 2014; 25:227-35.
47. Skripak JM, Nash SD, Rowley H, Brereton NH, Oh S, Hamilton RG, et al. A randomized, double-blind, placebo-controlled study of milk oral immunotherapy for cow's milk allergy. Journal of Allergy & Clinical Immunology 2008; 122:1154-60.
48. Staden U, Blumchen K, Blankenstein N, Dannenberg N, Ulbricht H, Dobberstein K, et al. Rush oral immunotherapy in children with persistent cow's milk allergy. Journal of Allergy & Clinical Immunology 2008; 122:418-9.
49. Suarez-Farinas M, Chang H, Gimenez G, Grishina G, Getts R, Sampson HA. Precision medicine in food allergy: use of food protein epitope mapping and machine learning to predict 'sustained unresponsiveness' in milk-allergic patients following milk oral immunotherapy. Allergy 2016:57.
50. Suarez-Farinas M, Suprun M, Chang HL, Gimenez G, Grishina G, Getts R, et al. Predicting development of sustained unresponsiveness to milk oral immunotherapy using epitope-specific antibody binding profiles. J Allergy Clin Immunol 2019; 143:1038-46.
51. Takahashi M, Taniuchi S, Soejima K, Hatano Y, Yamanouchi S, Kaneko K. Two-weeks-sustained unresponsiveness by oral immunotherapy using microwave heated cow's milk for children with cow's milk allergy. Allergy Asthma Clin Immunol 2016; 12:44.
52. Torabi B, De Schryver S, Lejtenyi D, Baerg I, Chan ES, Ben-Shoshan M, et al. Maintenance milk oral immunotherapy at 9 months is associated with ongoing increases in casein-specific serum IgG4. Allergy, asthma and clinical immunology. Conference: allergen's 8th research conference. Canada 2016; 12.
53. Torabi B, De Schryver S, Rahman T, Lejtenyi D, Baerg I, Chan ES, et al. Improvement in Skin Testing Is Associated with Increases in Milk Component- Specific IgA and IgG4 during Milk Oral Immunotherapy. Journal of Allergy and Clinical Immunology 2016; 137:AB131.
54. Torabi B, Schneider O, Lejtenyi D, Ben-Shoshan M, Mazer BD. Salivary IgG4 Increases during Milk Oral Immunotherapy. Journal of Allergy and Clinical Immunology 2017; 139:AB255.

55. Vazquez-Ortiz M, Alvaro-Lozano M, Alsina L, Garcia-Paba MB, Piquer-Gibert M, Giner-Munoz MT, et al. Safety and predictors of adverse events during oral immunotherapy for milk allergy: severity of reaction at oral challenge, specific IgE and prick test. Clin Exp Allergy 2013; 43:92-102.
56. Wasserman RL, Sugerman RW, Ul Ain Kamili Q, Pence DM, Hague AR, Herbert M. Eosinophilic esophagitis like oral immunotherapy related syndrome (ELORS). Journal of Allergy and Clinical Immunology 2018; Conference:2018 American Academy of Allergy, Asthma and Immunology, AAAAI and World Allergy Organization, WAO Joint Congress. United States. 141 (2 Supplement 1) (pp AB258).
57. Wood RA, Kim JS, Lindblad R, Nadeau K, Henning AK, Dawson P, et al. A randomized, double-blind, placebo-controlled study of omalizumab combined with oral immunotherapy for the treatment of cow's milk allergy. Journal of Allergy & Clinical Immunology 2016; 137:1103-10.e11.
58. Yanagida N, Sato S, Asaumi T, Okada Y, Ogura K, Ebisawa M. A Single-Center, Case-Control Study of Low-Dose-Induction Oral Immunotherapy with Cow's Milk. International Archives of Allergy & Immunology 2015; 168:131-7.
59. Yonekura Anagusko CL, Mendonca J, Lopes MM, Torres P, Ferreira Martins CS, Castro FM, et al. Specific IgG4 to milk proteins during oral immunotherapy for milk allergy: relationship to eosinophilic esophagitis. Journal of Allergy and Clinical Immunology 2019; Conference:2019 AAAAI Annual Meeting. United States. 143 (2 Supplement) (pp AB138).
60. Zapatero L, Alonso E, Fuentes V, Martinez MI. Oral desensitization in children with cow's milk allergy. J Investig Allergol Clin Immunol 2008; 18:389-96.

Author(s): RT, AB, JLB

Omalizumab compared to no anti-IgE antibody for patients who receive OIT for IgE-mediated CMA

Patient or population: patients who receive OIT for IgE-mediated CMA
Setting: tertiary care allergy clinic

Outcomes	№ of participants (studies) Follow up	Certainty of the evidence (GRADE)	Relative effect (95% CI)	Anticipated absolute effects	
				Risk with no anti-IgE antibody	Risk difference with omalizumab
Anaphylaxis	139 (3 RCTs) [1,2,3] follow up range: 9 to 26 months	⊕◯◯◯ VERY LOW [a,b,c]	**RR 0.34** (0.11 to 1.05)	10 per 100	**7 fewer per 100** (9 fewer to 1 more)
	125 (11 NRS) [4]	⊕◯◯◯ VERY LOW [a,b,c,d]	One study of OIT with multiple foods (n=41) reported 1 event in omalizumab group and 1 event in a control group (**RR: 1.93; 95% CI: 0.13 to 28.6**) (Otani 2014).<br>At least 1 event occurred in a median of **34%** patients receiving OIT+ omalizumab (**range: 11% to 60%**) (8 series of cases of milk OIT; n=74) (Nadeau 2011, Crisafulli 2019, Martorell-Calatayud 2016, Arasi 2017, Blasco-Valero 2017, Demir 2018, Larrosa Garcia 2019, Paz 2019).<br>Two additional series of cases of omalizumab+OIT with peanuts and multiple foods reported either no anaphylaxis (0/34) or 20 anaphylactic reactions among 23 patients (Andorf 2017, Brandstrom 2019).		
Use of IM epinephrine (adrenaline)	195 (4 RCTs) [1,2,3,5]	⊕◯◯◯ VERY LOW [a,b,c,e]	**RR 0.23** (0.05 to 0.97) [f]	25 per 100	**19 fewer per 100** (24 fewer to 1 fewer) [f]
	(7 NRS)	⊕◯◯◯ VERY LOW [a,b,c,d,g]	One study of OIT with multiple foods (n=41) reported 1 event in omalizumab group and 1 event in a control group (**RR: 1.93; 95% CI: 0.13 to 28.6**) (Otani 2014).<br>Studies with omalizumab + milk OIT: **21%** patients (**range: 0% to 60%**) (4 series of cases; n=33) (Nadeau 2011, Crisafulli 2019, Takahasi 2017, Demir 2018).<br>Studies with omalizumab + peanut OIT: 23% and 47% patients (Brandstrom 2019, Schneider 2013; n=30).		
Adverse effect leading to the discontinuation of treatment	101 (2 RCTs) [2,3]	⊕◯◯◯ VERY LOW [a,b,h]	**RR 0.41** (0.03 to 5.30)	9 per 100	**5 fewer per 100** (8 fewer to 37 more)
	(10 NRS)	⊕◯◯◯ VERY LOW [a,b,d]	One study of OIT with multiple foods (n=41) reported no events (Otani 2014).<br>Studies with omalizumab + milk OIT: **5%** patients (**range: 0% to 33%**) (7 series of cases; n=58) (Nadeau 2011, Crisafulli 2019, Martorell-Calatayud 2016, Takahasi 2017, Arasi 2017, Larrosa Garcia 2019, Paz 2019).<br>Studies with omalizumab + peanut or multiple foods OIT: **18%** patients (4 series of cases; n=68) (Andorf 2017, Brandstrom 2019, Schneider 2013, Le 2014).		
Severe gastrointestinal symptoms	55 (3 RCTs) [1,3,5]	⊕◯◯◯ VERY LOW [b,h,i]	**RR 0.89** (0.51 to 1.56) [j]	50 per 100	**5 fewer per 100** (25 fewer to 28 more) [j]
	(3 NRS)	⊕◯◯◯ VERY LOW [a,b,i,k,l]	3 series of cases receiving omalizumab with OIT for peanut and multiple food allergy reported between **0%, 29%, and 38%** patients with GI symptoms (n=64). (Andorf 2017, Brandstrom 2019, NCT00932282).		
Severe respiratory symptoms/wheezing	55 (3 RCTs) [1,3,5]	⊕◯◯◯ VERY LOW [b,h,i]	**RR 0.52** (0.34 to 1.48) [m]	29 per 100	**14 fewer per 100** (19 fewer to 14 more) [m]
	(6 NRS)	⊕◯◯◯ VERY LOW [a,i,k,l]	Six series of cases receiving omalizumab with OIT for milk, peanut, and multiple food allergy reported between **0%** and **73%** patients with respiratory symptoms (n=86). (Nadeau 2011, Crisafulli 2019, Arasi 2017, Andorf 2017, Brandstrom 2019, NCT00932282).		

Generalized erythema or urticaria	115<br>(3 RCTs) [1,3,5]	⊕◯◯◯<br>VERY LOW [a,b,h,i,n]	**RR 0.52**<br>(0.18 to 1.52) [o]	48 per 100	**23 fewer per 100**<br>(39 fewer to 25 more) [o]
	(5 NRS)	⊕◯◯◯<br>VERY LOW [a,b,i,k]	Five series of cases receiving omalizumab with OIT for milk, peanut, and multiple food allergy reported between **8%** and **41%** patients with urticaria (n=52). (Nadeau 2011, Crisafulli 2019, Arasi 2017, Brandstrom 2019, NCT00932282).		
Ability to drink cow's milk and eat dairy products without a reaction<br>assessed with: passing a graded food challenge with ≥150 ml cow's milk and/or ability to drink cow's milk and eat dairy products without symptoms	55<br>(1 RCT) [3]<br>follow up: 7 months	⊕⊕◯◯<br>LOW [c,h,p]	**RR 1.24**<br>(0.95 to 1.63)	71 per 100	**17 more per 100**<br>(4 fewer to 45 more)
Ability to accidentally consume a small amount of cow's milk without a reaction<br>assessed with: passing a supervised graded food challenge with ≥5 ml of cow's milk	64<br>(2 RCTs) [1,3]	⊕⊕◯◯<br>LOW [e,q]	**RR 1.26**<br>(0.96 to 1.64)	69 per 100	**18 more per 100**<br>(3 fewer to 44 more)
Ability to drink cow's milk reintroduced after a period of not consuming milk and milk products<br>assessed with: passing a graded food challenge with ≥150 ml cow's milk and/or ability to drink cow's milk and eat dairy products without symptoms	75<br>(2 RCTs) [3,5]	⊕◯◯◯<br>VERY LOW [h,r]	**RR 1.42**<br>(0.82 to 2.44)	35 per 100	**15 more per 100**<br>(6 fewer to 51 more)
Ability to accidentally consume a small amount of cow's milk reintroduced after a period of not consuming milk and milk products<br>assessed with: passing a supervised graded food challenge with ≥5 ml of cow's milk	75<br>(2 RCTs) [3,5]	⊕◯◯◯<br>VERY LOW [b,h]	**RR 1.02**<br>(0.73 to 1.40)	49 per 100	**1 more per 100**<br>(13 fewer to 19 more)
Emergency department visit	17<br>(1 NRS)	⊕◯◯◯<br>VERY LOW [a,b,k]	Only one case series of patients receiving omalizumab and OIT for peanut allergy reported 7/17 patients requiring ED visits. Other studies did not report this outcome.		
Hospital admission	22<br>(2 NRS)	⊕◯◯◯<br>VERY LOW [a,b,k]	Two series of cases reported 0/5 and 2/17 patients requiring hospitalization. (Crisafulli 2019, Brandstrom 2019).		
Eosinophilic esophagitis	(4 RCTs) [1,2,3,5]	⊕◯◯◯<br>VERY LOW [b]	Four RCTs reported **3 cases among 131 patients** receiving omalizumab with OIT and **1 case among 68 patients** receiving OIT alone. Two additional series of patients receiving omalizumab with OIT reported 1 case among 30 patients (Brandstrom 2019, Schneider 2013).		
Quality of life of patients	(2 NRS) [2,4]	⊕◯◯◯<br>VERY LOW [b]	One RCT reported that patient QoL improved (allergen avoidance, social/dietary limitations, anxiety). Authors also observed a reduction in child and parent perceived risk of severe reactions and death from accidental ingestion. However, they did not assess the difference between the omalizumab and control groups because most control patients received open label omalizumab before the QoL was measured. One observational study with a control group reported the number of patients who achieved at least minimal important improvement in QoL (**RR: 1.2**, 95% CI: 0.91 to 1.58; **RD: 15 more per 100 patients**; 95% CI: from 8 fewer to 38 more).<br>Another study among patients receiving omalizumab for OIT with milk reported that patients saw the following benefits:<br>- increased dietary options (4/9 children)<br>- inclusion in social situations (parties/cafeteria, 4/9 children)<br>- decreased anxiety about reactions (3/9).<br>They also reported the following factors reducing QoL:<br>- omalizumab injections and blood draws (7/9)<br>- worry about possible reactions during desensitization or challenges (5/9).		

Quality of life of the caregivers	(2 NRS) [2,4]	⊕◯◯◯ VERY LOW [b]	One RCT reported a reduction in caregiver perceived risk of severe reactions and death from accidental ingestion. However, they did not assess the difference between the omalizumab and control groups because most control patients received open label omalizumab before the QoL was measured. Another study of patients receiving omalizumab for OIT with milk reported that caregivers saw the following benefits:<br>- reduced anxiety about allergic reactions (5/8)<br>- child's inclusion in social activities (4/8)<br>- ability to eat at restaurants (4/8)<br>- increased spontaneity around food-related events (3/8).
Any adverse effect<br>follow up: 9 months	(4 RCTs) [1,2,3,5]	⊕◯◯◯ VERY LOW [b,h]	In the one RCT that used OIT for milk allergy the median percentage of doses per person with any symptoms was 2.1% with omalizumab and 16.1% without. Reactions requiring treatment were also more frequent without omalizumab (median: 0.0% vs. 3.8% of doses per person). Most reaction were classified by study authors as mild and majority were oral or pharyngeal (median: 0.6% vs. 8.8% of doses per person), respiratory symptoms (median: 0.0 vs. 2.5% of doses per person) and GI symptoms (median 0.0 vs. 3.0% of doses per person). The other 3 RCTs that used omalizumab with OIT for other foods reported rates of adverse effects. The pooled IRR was 0.64 (95% CI: 0.21 to 1.97) which corresponds to 304 fewer AEs per 100 patient-years (95% CI: from 675 fewer to 827 more).

***The risk in the intervention group** (and its 95% confidence interval) is based on the assumed risk in the comparison group and the **relative effect** of the intervention (and its 95% CI).

CI: Confidence interval; **NRS:** non-randomized study; **RCT:** randomized controlled trial; **RR:** Risk ratio

GRADE Working Group grades of evidence
High certainty: We are very confident that the true effect lies close to that of the estimate of the effect
Moderate certainty: We are moderately confident in the effect estimate: The true effect is likely to be close to the estimate of the effect, but there is a possibility that it is substantially different
Low certainty: Our confidence in the effect estimate is limited: The true effect may be substantially different from the estimate of the effect
Very low certainty: We have very little confidence in the effect estimate: The true effect is likely to be substantially different from the estimate of effect

Explanations

a. There is some uncertainty whether or not the baseline risk for this outcome is the same during OIT with milk and with other foods used in these studies: peanut, egg, and multiple foods at the same time.
b. Number of events and total number of participants are small and do not meet optimal information size.
c. Numerous small studies have only been published as conference abstracts without a corresponding peer-reviewed publication.
d. The only observational study with a control group did not adjust for any confounders and the remaining studies were series of cases without control groups.
e. Confidence interval does not exclude an appreciable benefit or no difference with adding omalizumab to OIT.
f. Studies also reported number of epinephrine injections per group and the overall effect was similar: pooled incidence rate ratio: 0.36 (95% CI: 0.13 to 1.01); rate difference: 19 fewer per 100 patient-years (95% CI: 0 to 26 fewer).
g. Epinephrine use varied widely.
h. Confidence interval does not exclude an appreciable benefit or an appreciable harm from adding omalizumab to OIT.
i. The severity of symptoms was not reported; it is likely that they represented the whole spectrum from mild to severe.
j. Presented results are from one RCT in patients with milk OIT. Another study reported incidence rate and its results were consistent with those presented (IRR: 0.81, 95% CI: 0.52 to 1.26; rate difference: 48 fewer per 100 patient-years, 95% CI: 153 fewer to 57 more). A third study reported GI adverse effects in 22% vs. 54% of doses received by patients in omalizumab vs. control group.
k. Single arm study without a control group.
l. Estimates varied but we did not lower certainty because the numbers were small and most likely they were the main source of observed inconsistency.
m. Presented results are from one RCT in patients with milk OIT. Another study reported incidence rate and its results were consistent with those presented (IRR: 0.71, 95% CI: 0.34 to 1.48; rate difference: 28 fewer per 100 patient-years, 95% CI: 91 fewer to 35 more). A third study reported GI adverse effects in 0% vs. 1% of doses received by patients in omalizumab vs. control group.
n. Studies showed different results, but we assumed that it could be explained by different definitions and different severity of symptoms that were counted.
o. One study reported incidence rate and its results were consistent with those presented (IRR: 0.92, 95% CI: 0.47 to 1.78; rate difference: 11 fewer per 100 patient-years, 95% CI: 97 fewer to 75 more).
p. Only 44 events
q. One study (Wood 2016) did not report this outcome and we extrapolated from ability to drink 150 ml of milk. It is likely that there were some additional patients who achieved this outcome.
r. Only 23 events

References

1. Wood RA, Kim JS, Lindblad R, Nadeau K, Henning AK, Dawson P, et al. A randomized, double-blind, placebo-controlled study of omalizumab combined with oral immunotherapy for the treatment of cow's milk allergy. Journal of Allergy & Clinical Immunology. 2016;137(4):1103-10.e11.
2. MacGinnitie AJ, Rachid R, Gragg H, Little SV, Lakin P, Cianferoni A, et al. Omalizumab facilitates rapid oral desensitization for peanut allergy. Journal of Allergy & Clinical Immunology. 2017;139(3):873-81.e8.
3. Andorf S, Purington N, Block WM, Long AJ, Tupa D, Brittain E, et al. Anti-IgE treatment with oral immunotherapy in multifood allergic participants: a double-blind, randomised, controlled trial. Lancet Gastroenterol Hepatol. 2018;3(2):85-94.
4. Andorf S, Purington N, Kumar D, Long A, O'Laughlin KL, Sicherer S, et al. A Phase 2 Randomized Controlled Multisite Study Using Omalizumab-facilitated Rapid Desensitization to Test Continued vs Discontinued Dosing in Multifood Allergic Individuals. EClinicalMedicine. 2019;7:27-38.
5. Acevedo M, Fuentes-Aparicio V, Zambrano G, Morales-Cabeza C, Infante S, Alvarez-Perea A, et al. Oral immunotherapy and follow-up in highly sensitized egg allergic children. Allergy: European Journal of Allergy and Clinical Immunology. 2017;72 (Supplement 103):739.

6. Otani IM, Begin P, Kearney C, Dominguez TL, Mehrotra A, Bacal LR, et al. Multiple-allergen oral immunotherapy improves quality of life in caregivers of food-allergic pediatric subjects. Allergy Asthma Clin Immunol. 2014;10(1):25.
7. Arasi S, Caminiti L, Crisafulli G, Pajno GB. Omalizumab combined with oral immunotherapy for the treatment of severe cow's milk allergy: Our 2-year-long experience. Allergy: European Journal of Allergy and Clinical Immunology. 2017;72 (Supplement 103):740-1.
8. Blasco-Valero C, Galvan-Blasco P, Garriga-Baraut T, Vila-Indurain B. Improvement of safety during the induction and maintenance phase of oral immunotherapy with cow's milk and egg when assisted with omalizumab. Clinical and Translational Allergy Conference: 5th Pediatric Allergy and Asthma Meeting, PAAM. 2017;8(Supplement 2).
9. Candon Morillo R, Burgos Montero AM, Moreno Mata E, Ruiz Leon MB, Gonzalez Sanchez LA. Results of tretament of XOLAIR (omalizumab) associated with oral immunotherapy of cow's milk in anfilactic patient. Allergy: European Journal of Allergy and Clinical Immunology. 2014;69:537.
10. Crisafulli G, Caminiti L, Chiera F, Arasi S, Salzano G, Panasiti I, et al. Omalizumab in children with severe allergic disease: a case series. Ital. 2019;45(1):13.
11. Demir E, Eren Akarcan S, Cigerci Gunaydin N, Ulusoy Severcan E, Kartal Ozturk G, Eski A, et al. Omalizumab for patients refractory to milk oral immunotherapy. Allergy: European Journal of Allergy and Clinical Immunology. 2018;73 (Supplement 105):501.
12. Larrosa Garcia M, Jimenez-Lozano I, Blasco-Valero C, Canete-Ramirez C, Garriga-Baraut MT, Garcia-Palop BM, et al. Analysis of the use of omalizumab in oral tolerance induction for high-risk food allergies in children. European Journal of Hospital Pharmacy. 2019;26 (Supplement 1):A132.
13. Lefevre S, Kanny G. Oral immunotherapy and omalizumab for food allergy. Allergy: European Journal of Allergy and Clinical Immunology. 2016;71 (Supplement 102):269.
14. Martorell-Calatayud C, Michavila-Gomez A, Martorell-Aragones A, Molini-Menchon N, Cerda-Mir JC, Felix-Toledo R, et al. Anti-IgE-assisted desensitization to egg and cow's milk in patients refractory to conventional oral immunotherapy. Pediatric Allergy & Immunology. 2016;27(5):544-6.
15. Nadeau KC, Schneider LC, Hoyte L, Borras I, Umetsu DT. Rapid oral desensitization in combination with omalizumab therapy in patients with cow's milk allergy. Journal of Allergy & Clinical Immunology. 2011;127(6):1622-4.
16. Paz LF, Barranco RM, Fernandez Crespo JF, Del Carmen Dieguez M. Safety of oral immunotherapy in addition to omalizumab in patients with severe allergy to cow's milk proteins. Journal of Allergy and Clinical Immunology. 2019;143 (2 Supplement):AB276.
17. Takahashi M, Soejima K, Taniuchi S, Hatano Y, Yamanouchi S, Ishikawa H, et al. Oral immunotherapy combined with omalizumab for high-risk cow's milk allergy: a randomized controlled trial. Sci. 2017;7(1):17453.
18. Brandstrom J, Vetander M, Sundqvist AC, Lilja G, Johansson SGO, Melen E, et al. Individually dosed omalizumab facilitates peanut oral immunotherapy in peanut allergic adolescents. Clinical & Experimental Allergy. 2019;49(10):1328-41.
19. Schneider LC, Rachid R, LeBovidge J, Blood E, Mittal M, Umetsu DT. A pilot study of omalizumab to facilitate rapid oral desensitization in high-risk peanut-allergic patients. Journal of Allergy & Clinical Immunology. 2013;132(6):1368-74.
20. Sindher S, Kumar D, Purington N, Tupa D, Long A, Cao S, et al. Efficacy of a Fixed Dose of Omalizumab during Multi-Allergen Oral-Immunotherapy. Annals of Allergy, Asthma and Immunology. 2019;123 (5 Supplement):S50-S1.
21. Dominguez TLR, Mehrotra A, Wilson S, Winterroth L, Blakemore A, Sampson K, et al. The safety of multiple oral immunotherapy in phase one studies at a single center. Clinical and Translational Allergy. 2013;3:24DUMMY.
22. Henson M, Edie A, Steele P, Kamilaris J, Kulis M, Thyagarajan A, et al. Peanut oral immunotherapy and omalizumab treatment for peanut allergy. Journal of Allergy and Clinical Immunology. 2012;129(2):AB28.
23. Le U, Virkud Y, Vickery BP, Steele PH, Kamilaris JS, Kulis Jr MD, et al. Omalizumab pretreatment does not protect against peanut oral immunotherapy-related adverse gastrointestinal events. Journal of Allergy and Clinical Immunology. 2014;133(2):AB104.
24. Lyu SC, Nadeau K. Mechanisms of Th2 to treg vs Th2 to Th1 in non rush vs rush food OIT. Journal of Allergy and Clinical Immunology. 2013;131(2):AB196.
25. Pena-Peloche M, Hinojosa Macias M, De La Hoz Caballer B, Terrados Cepeda S, Berges Gimeno M, Huertas Barbudo B, et al. Treatment of severe and persistent food allergy with Omalizumab. Journal of Allergy and Clinical Immunology. 2011;127(2):AB26.
26. NCT00932282. Peanut Oral Immunotherapy and Anti-Immunoglobulin E (IgE) for Peanut Allergy. https://clinicaltrialsgov/show/NCT00932282. 2009.

OIT with baked milk compared to no OIT in patients with IgE-CMA who do not tolerate baked milk

Patient or population: patients with IgE-CMA who do not tolerate baked milk
Setting:
Intervention: OIT with baked milk
Comparison: no OIT

Outcomes	№ of participants (studies) Follow up	Certainty of the evidence (GRADE)	Impact
Anaphylaxis<br>follow up: 1 years	(1 observational study) [1]	⊕◯◯◯<br>VERY LOW [a,b,c]	**With OIT:** 1/20 patients in one series (**5%**).<br>**Without OIT:** based on studies among patients with CMA in whom tolerance of baked milk was not known (OIT with fresh milk) the rate of anaphylaxis with elimination diet alone would be 1 per 100 persons per year (**1%**).
Use of IM epinephrine<br>follow up: 1 years	(2 observational studies) [1,2]	⊕◯◯◯<br>VERY LOW [a,c,d]	**With OIT:** 3/15 (**20%**) in one study and 1/20 (**5%**) in another study.<br>**Without OIT:** based on studies among patients with CMA in whom tolerance of baked milk is not known (OIT with fresh milk) the rate of epinephrine use with elimination diet alone would be 2 per 120 patients (**1.7%**).
Discontinuation of treatment due to adverse effects and/or symptoms<br>follow up: 1 years	(2 observational studies) [1,2]	⊕◯◯◯<br>VERY LOW [a,c,d]	2/15 (**13%**) in one study and 4/20 (**20%**) in another study.
Severe gastrointestinal symptoms<br>follow up: 1 years	(2 observational studies) [1,2]	⊕◯◯◯<br>VERY LOW [a,c,d,e]	5/15 (**33%**) in one study and 3/20 (**15%**) in another study.
Severe respiratory symptoms/wheezing<br>follow up: 1 years	(2 observational studies) [1,2]	⊕◯◯◯<br>VERY LOW [a,c,d,e]	8/15 (**53%**) in one study and 2/20 (**10%**) in another study.
Generalized urticaria or erythema<br>follow up: 1 years	(1 observational study) [2]	⊕◯◯◯<br>VERY LOW [a,c,f]	5 of 15 patients (**33%**)
Ability to drink cow's milk and eat dairy products without a reaction<br>assessed with: passing a supervised graded food challenge with >254 ml of fresh cow's milk or ability to eat 1.3 g of baked milk<br>follow up: 1 years	(2 observational studies) [1,2]	⊕◯◯◯<br>VERY LOW [a,c,d]	4/15 (**27%**) in one study and 5/20 (**25%**) in another study.
Ability to accidentally consume a small amount of cow's milk without a reaction - not reported	-	-	
Ability to drink cow's milk reintroduced after a period of abstaining from milk and milk products - not measured	-	-	
Emergency department visit - not reported	-	-	
Hospital admission - not reported	-	-	

Eosinophilic esophagitis - not reported	-	-	
Quality of life of children - not measured	-	-	
Quality of life of the caregivers - not measured	-	-	
Lip/mouth pruritus - not reported	-	-	
Angioedema - not reported	-	-	
Any adverse effect - not reported	-	-	

***The risk in the intervention group** (and its 95% confidence interval) is based on the assumed risk in the comparison group and the **relative effect** of the intervention (and its 95% CI).

CI: Confidence interval

GRADE Working Group grades of evidence
High certainty: We are very confident that the true effect lies close to that of the estimate of the effect
Moderate certainty: We are moderately confident in the effect estimate: The true effect is likely to be close to the estimate of the effect, but there is a possibility that it is substantially different
Low certainty: Our confidence in the effect estimate is limited: The true effect may be substantially different from the estimate of the effect
Very low certainty: We have very little confidence in the effect estimate: The true effect is likely to be substantially different from the estimate of effect

Explanations

a. No control group (series of cases). Any inference requires an implicit comparison with another group of patients who did not receive OIT. We did not identify any study that explicitly measured and reported the rate of adverse reactions among patients allergic to baked milk.
b. Only one event
c. One additional study (Lazzarotto 2013, Lazzarotto 2014) has been completed and published only as a conference abstract with no information about the outcomes.
d. Few events among only 35 patients
e. Studies did not report how severe were the symptoms.
f. Most studies reported urticaria without mentioning its range or severity.

References

1. Gruzelle, V., Juchet, A., Martin-Blondel, A., Michelet, M., Chabbert-Broue, A., Didier, A.. Benefits of baked milk oral immunotherapy in French children with cow's milk allergy. Pediatric Allergy and Immunology.; 2020.
2. Goldberg, M. R., Nachshon, L., Appel, M. Y., Elizur, A., Levy, M. B., Eisenberg, E., Sampson, H. A., Katz, Y.. Efficacy of baked milk oral immunotherapy in baked milk-reactive allergic patients. Journal of Allergy & Clinical Immunology; Dec 2015.

World Allergy Organization (WAO) Diagnosis and Rationale for Action against Cow's Milk Allergy (DRACMA) Guidelines 2021: immunotherapy

Author(s): Ramon Targino, Jan Brozek

Question: Epicutaneous immunotherapy compared to no immunotherapy for cow's milk allergy

Outcome	№ of studies	№ of patients		Effect		Certainty	Importance
		epicutaneous immunotherapy	no immunotherapy	Relative (95% CI)	Absolute (95% CI)		
**Death (follow up: 3 to 12 months)**	2 [1,2]	0/155 (0.0%)	0/62 (0.0%)	not estimable	**0 fewer per 100** (from 3 fewer to 2 more)	⊕⊕⊕⊕ HIGH [a]	CRITICAL
**Anaphylaxis (follow up: 3 months)**	2 [1,2]	0/155 (0.0%)	0/62 (0.0%)	not estimable	**0 fewer per 100** (from 3 fewer to 2 more)	⊕◯◯◯ VERY LOW [b,c,d,e,f]	CRITICAL
**Epinephrine use**	1 [1]	0/10 (0.0%)	0/9 (0.0%)	not estimable	**0 fewer per 100** (from 18 fewer to 18 more)	⊕◯◯◯ VERY LOW [b,c,e,f,g,h]	CRITICAL
**Laryngeal edema (follow up: 3 to 12 months)**	2 [1,2]	Laryngeal edema was not explicitly reported but we assumed that no child suffered this outcome based on reported number of anaphylactic reactions and serious adverse effects. The risk difference would be 0 more per 1,000 (95% CI: 33 fewer to 21 more).				⊕◯◯◯ VERY LOW [b,c,d,e,f]	CRITICAL
**Severe asthma/wheezing**	1 [1]	0/10 (0.0%)	0/9 (0.0%)	not estimable	**0 fewer per 100** (from 18 fewer to 18 more)	⊕◯◯◯ VERY LOW [b,c,e,f,h]	CRITICAL
**Serious adverse effects (follow up: 3 and 12 months)**	2 [1,2]	0/155 (0.0%)	0/62 (0.0%)	not estimable	**0 fewer per 100** (from 2 fewer to 3 more)	⊕◯◯◯ VERY LOW [b,c,d,e,f]	CRITICAL
**Discontinuation of treatment owing to adverse effects (follow up: 3 months)**	2 [1,2]	Ona small study (Dupont 2010) reported no discontinuation owing to AEs and the other study (Tilles 2018) reported only 1.5% dropout rate owing to AEs (but did not report in which group).				⊕◯◯◯ VERY LOW [b,c,f,l]	IMPORTANT
**Any adverse effects (follow up: 12 months)**	2 [1,2]	There were no serious adverse effects reported in both studies. No patient discontinued therapy because of adverse effect. There were also no hospitalizations, ED visits, no epinephrine was used, and no anaphylactic reaction occurred. One study (Dupont 2010) reported 2 patients in EPIT having diarrhea and 1 patient vomiting, compared to no one in placebo group. There were 1 vs 2 episodes of wheezing. In another study (MILES) most patients reported local itching (83.3%), redness (83.3%) or swelling (72.2%) at least once, but the difference between the groups was not reported.				⊕◯◯◯ VERY LOW [b,c,f,j,l]	CRITICAL
**Emergency department visit**	2 [1,2]	0/155 (0.0%)	0/62 (0.0%)	not estimable	**0 fewer per 100** (from 3 fewer to 2 more)	⊕◯◯◯ VERY LOW [b,c,f,l]	CRITICAL
**Hospital admission**	2 [1,2]	0/155 (0.0%)	0/62 (0.0%)	not estimable	**0 fewer per 100** (from 3 fewer to 2 more)	⊕◯◯◯ VERY LOW [b,c,f,l]	CRITICAL
**Full tolerance (able to drink 150 mL of milk and/or eat dairy products)**	1 [1]	In one study no child in either group (EPIT 10, placebo 8) was able to tolerate 100 ml of milk after 3 months of EPIT (Dupont 2010). Exact numbers were not reported but the mean change in tolerated dose of milk was from 2 to 23 ml in EPIT group and from 4 to 5 ml in placebo group).				⊕◯◯◯ VERY LOW [b,c,f,h]	CRITICAL

**At least partial tolerance (able to drink ≥5 mL of milk) (follow up: 3 and 12 months)**	2 [1,2]	63/154 (40.9%)	17/60 (28.3%)	RR 1.41 (0.90 to 2.19)	**12 more per 100** (from 3 fewer to 34 more)	⊕◯◯◯ VERY LOW [b,c,f,k,l]	CRITICAL
**Cumulative dose of milk tolerated during OFC**	1 [1]	In one study among 18 children the mean difference was 18.17 ml more (1.02 less to 37.36 more) (Dupont 2010). In another study the mean change from baseline in amount of milk tolerated was 22–36 ml in EPIT groups and 17 ml in placebo group (Tilles 2018). The baseline tolerance was not reported but children were included in the study when they tolerated less than 10 ml of cow's milk.				⊕◯◯◯ VERY LOW [b,c, f,h,m]	IMPORTANT
**Duration of sustained tolerance of milk (when achieved) - not measured**	-	-	-	-	-	-	CRITICAL
**Outgrowing CMA - not measured**	-	-	-	-	-	-	CRITICAL
**Quality of life of a patient - not measured**	-	-	-	-	-	-	CRITICAL
**Quality of life of caregivers - not measured**	-	-	-	-	-	-	CRITICAL

CI: Confidence interval; **RR:** Risk ratio; **MD:** Mean difference

Explanations

a. No events, however, given the very low mortality from food allergy in general, we assumed that EPIT would have no influence on this outcome.
b. In 2 abstracts from this study (Dupont 2010) the number of children receiving EPIT was 13 and in the other abstract and in the final publication it is 9 -- unclear why those 4 children were excluded.
c. Both studies were published only as conference abstracts and as press releases from the manufacturer.
d. There were no events in these studies; risk difference is estimated based on total number of patients (this estimate is conservative and likely produces confidence intervals that are too wide).
e. No events; confidence interval does not exclude an appreciable benefit or an appreciable harm.
f. There are only 2 small studies published over the last 9 years and both are sponsored by the same manufacturer of the same device.
g. One study with most patients did not report this outcome.
h. Only 16 patients
i. Only 16 patients in one study and 198 patients in another, but differences not reported per group. Number of individual events would likely not meet the optimal information size.
j. One study with most patients did not report results per group but only total events across all treatments.
k. It is not clear how many children benefited because the inclusion criterion for the study that contributed almost all information was inability to tolerate 9 ml of milk (unclear how many children were included and could tolerate 5 ml to start with).
l. Only 80 events; confidence interval does not exclude an appreciable benefit or no difference
m. Per protocol analysis

References

1. Dupont, C., Kalach, N., Soulaines, P., Legoue-Morillon, S., Piloquet, H., Benhamou, P. H.. Cow's milk epicutaneous immunotherapy in children: a pilot trial of safety, acceptability, and impact on allergic reactivity. Journal of Allergy & Clinical Immunology; 2010.
2. Tilles, S., Makhija, M., Hebert, J., Nadeau, K., Begin, P., Brown Whitehorn, T., Rutault, K., Hayem, C., Nowak-Wegrzyn, A., Wood, R.. A double-blind, placebo-controlled phase I/II dose-finding study of viaskin milk in children and adolescents for the treatment of IgE-mediated cow's milk protein allergy (CMPA): Results from miles. Allergy: European Journal of Allergy and Clinical Immunology; 2018.

Membership of the panel and the evidence synthesis team

DRACMA guideline panel	Evidence synthesis team
Ignacio Ansotegui<br>Amal H. Assa'ad<br>Sami L. Bahna<br>Roberto Berni Canani<br>Martin Bozzola<br>Jan L. Brozek (non-voting)<br>Lamia Dahdah<br>Christophe Dupont<br>Motohiro Ebisawa<br>Alessandro Fiocchi<br>Elena Galli<br>Rose Kamenwa<br>Gideon Lack<br>Haiqi Li<br>Alberto Martelli<br>Anna Nowak-Węgrzyn<br>Nikolaos G. Papadopoulos<br>Ruby Pawankar<br>Maria Said<br>Mario Sánchez-Borges<br>Holger J. Schünemann<br>Raanan Shamir<br>Jonathan M. Spergel<br>Hania Szajewska<br>Luigi Terracciano<br>Yvan Vandenplas<br>Carina Venter<br>Susan Waserman<br>Amena Warner<br>Gary W. K. Wong	Stefania Arasi<br>Antonio Bognanni<br>Jan L. Brozek<br>Derek K. Chu<br>Piotr Dziechciarz<br>Ramon T. Firmino<br>Andrea Horvath<br>Yetiani Roldan<br>Siw Waffenschmidt
