# Supplementary material for: World Allergy Organization (WAO) Diagnosis and Rationale for Action against Cow’s Milk Allergy (DRACMA) Guideline update – XIV – Recommendations on CMA immunotherapy
Source: World Allergy Organ J. 2022 Apr 23;15(4):100646. doi: 10.1016/j.waojou.2022.100646 (PMC9061625; doi:10.1016/j.waojou.2022.100646)
Supplement: Multimedia component 4 [file mmc4.docx]

**Supplemental Appendix 1**

**Search strategies**

**1. Search for systematic reviews (since 2010)**

***PubMed (NLM)***

#1 "meta analysis"[Publication Type]

#2 "review"[Publication Type]

#4 meta analysis[all fields]

#5 search*[tw]

#6 #1 or #2 or #4 or #5

#7 milk AND (allergy OR allergic OR allergen OR intolerance* OR tolerance* OR hypersensitive)

#8 #6 and #7

#9 #8 AND 2010:2018 [DP]

***Cochrane Database of Systematic Reviews (CDSR)***

(milk AND (allergy OR allergic OR allergen OR intolerance* OR tolerance* OR hypersensitive)):ti,ab,kw

***Database of Abstracts of Reviews of Effects (DARE)***

(milk AND (allergy OR allergic OR allergen OR intolerance* OR tolerance* OR hypersensitive)) IN DARE, HTA WHERE LPD FROM 01/01/2010 TO 24/09/2018

***National Institute for Health and Care Excellence (NICE)***

Search term: milk

***Canadian Agency for Drugs and Technologies in Health (CADTH)***

Search terms: milk allergy

***Agency for Healthcare Research and Quality (AHRQ)***

Search term: milk

***Epistemonikos***

Search terms: milk allergy

**2. Search for individual studies**

***MEDLINE (OVID)***

1 Milk Hypersensitivity/

2 Milk/

3 (milk adj3 (allerg* or hypersensitivity)).ti,ab.

4 or/1-3

5 Milk Hypersensitivity/im

6 Desensitization, Immunologic/

7 (oral adj5 (desensiti* or immunotherapy or tolerance induction)).ti,ab.

8 or/5-7

9 and/4,8

10 9 not (exp animals/ not humans.sh.)

11 10 not (comment or editorial).pt.

12 11 not (review or meta-analysis or guideline or practice guideline).pt.

***PubMed (NLM)***

#1 milk[tiab] AND (hypersensitivity[tiab] OR allerg*[tiab])

#2 oral[tiab] AND (desensiti*[tiab] OR immunotherapy[tiab] OR tolerance induction[tiab])

#3 #1 AND #2

#4 #3 NOT Medline [SB]

***Embase (OVID)***

1 milk allergy/

2 (milk adj3 (allerg* or hypersensitivity)).ti,ab.

3 or/1-2

4 desensitization/

5 immunological tolerance/

6 exp immunotherapy/

7 (oral adj5 (desensiti* or immunotherapy or tolerance induction)).ti,ab.

8 or/4-7

9 and/3,8

10 9 not (exp animals/ not human/)

11 10 not (Conference Abstract or Conference Review or Editorial).pt.

12 11 not medline.cr.

***Cochrane Central Register of Controlled Trials***

#1 MeSH descriptor: [Milk Hypersensitivity] explode all trees

#2 MeSH descriptor: [Milk] explode all trees

#3 (milk NEAR/3 (allerg* or hypersensitivity)):ti,ab

#4 #1 or #2 or #3

#5 MeSH descriptor: [Milk Hypersensitivity] explode all trees and with qualifier(s): [immunology - IM]

#6 MeSH descriptor: [Desensitization, Immunologic] explode all trees

#7 (oral NEAR/5 (desensiti* or immunotherapy or tolerance induction)):ti,ab

#8 #5 or #6 or #7
