# Supplementary material for: World Allergy Organization (WAO) Diagnosis and Rationale for Action against Cow’s Milk Allergy (DRACMA) Guideline update – XIV – Recommendations on CMA immunotherapy
Source: World Allergy Organ J. 2022 Apr 23;15(4):100646. doi: 10.1016/j.waojou.2022.100646 (PMC9061625; doi:10.1016/j.waojou.2022.100646)
Supplement: Multimedia component 5 [file mmc5.docx]

**Detailed description of Inclusion and Exclusion criteria**

**Systematic Review**

Inclusion criteria:

- Design: SR, guideline that includes SR(s)
- Populations: Patients with confirmed CMA as defined by the authors, with no restrictions of age or comorbidities, including other concomitant food allergies.
- Interventions: oral immunotherapy with cow’s milk (either fresh or baked).

Exclusion criteria:

- Design: independent studies
- Populations: non-IgE mediated cow’s milk allergy; sensitization to CM without clinical symptoms; other food allergies
- Interventions: immunotherapy administered solely through other routes than the oral one (i.e. Epicutaneous, Labial); OIT performed with the employment of an allergen different from cow’s milk (either fresh or baked).

**Independent studies**

Design: both RCTs and NSRs, including both comparative and single-arm

- Populations: Patients with confirmed CMA as defined by the authors, with no restrictions of age or comorbidities, including other concomitant food allergies.
- Interventions: oral immunotherapy with cow’s milk (either fresh or baked).

Exclusion criteria:

- Design: editorials, commentaries and letters to the editors with no primary data, non-human studies;
- Populations: non-IgE mediated cow’s milk allergy; sensitization to CM without clinical symptoms; different food allergies
- Interventions: immunotherapy administered solely through other routes than the oral one (i.e. Epicutaneous, Labial); OIT performed with the employment of an allergen different from cow’s milk (either fresh or baked).
