# Supplementary material for: World Allergy Organization (WAO) Diagnosis and Rationale for Action against Cow’s Milk Allergy (DRACMA) Guideline update – XIV – Recommendations on CMA immunotherapy
Source: World Allergy Organ J. 2022 Apr 23;15(4):100646. doi: 10.1016/j.waojou.2022.100646 (PMC9061625; doi:10.1016/j.waojou.2022.100646)
Supplement: Multimedia component 8 [file mmc8.docx]

Supplementary Table 3: Risk of bias of randomized controlled trials.

| **Study** | **Outcomes** | **Randomization process** | **Intended intervention** | **Missing outcome data** | **Measurement of outcome** | **Selection of reported outcome** | **Overall risk of bias** |
| --- | --- | --- | --- | --- | --- | --- | --- |
| De Schryver 2019 A | Anaphylaxis; Adverse effect leading to discontinuation of treatment; Ability to drink milk without reaction | Some concerns | Low risk of bias | Low risk of bias | Low risk of bias | Some concerns | Some concerns |
| Filho 2015 | Death | High risk of bias | Low risk of bias | Some concerns | Low risk of bias | Some concerns | High risk of bias |
| Gabrielli 2018 | No outcomes of interest | NA | NA | NA | NA | NA | NA |
| Lee 2013 | Anaphylaxis; Use of IM epinephrine; Adverse effect leading to discontinuation of treatment; Ability to drink milk without reaction; Ability to accidentally consume a small amount without reaction; Death; Lip or mouth pruritus; Any adverse effect. | High risk of bias | Low risk of bias | Low risk of bias | Low risk of bias | Some concerns | High risk of bias |
| Longo 2008 | Anaphylaxis; Use of IM epinephrine; Adverse effect leading to discontinuation of treatment; Severe GI symptoms; Severe respiratory symptoms; Generalized erythema, urticaria and angioedema; Ability to drink milk without reaction; Ability to accidentally consume a small amount without reaction; Emergency department visit; Death; Mild respiratory symptoms; Angioedema; Lip or mouth pruritus; Any adverse effect. | Low risk of bias | Low risk of bias | Low risk of bias | Low risk of bias | Some concerns | Some concerns |
| Maeda 2021 | Ability to accidentally consume a small amount without reaction; Severe Adverse Events; Use of IM epinephrine; Adverse effect leading to discontinuation of treatment; Any adverse event | Low risk of bias | Low risk of bias | Low risk of bias | Low risk of bias | Some concerns | Some concerns |
| Martorell 2011 | Anaphylaxis; Use of IM epinephrine; Adverse effect leading to discontinuation of treatment; Severe GI symptoms; Generalized erythema, urticaria and angioedema; Ability to drink milk without a reaction; Ability to accidentally consume a small amount without reaction; Death; Mild respiratory symptoms; Angioedema; Lip or mouth pruritus; Any adverse effect. | Low risk of bias | Low risk of bias | Low risk of bias | Low risk of bias | Some concerns | Some concerns |
| Morisset 2007 | Death; Mild respiratory symptoms | High risk of bias | Some concerns | Low risk of bias | Some concerns | Some concerns | High risk of bias |
| Pajno 2010 | Anaphylaxis; Use of IM epinephrine; Adverse effect leading to discontinuation of treatment; Severe GI symptoms; Generalized erythema, urticaria and angioedema; Ability to drink milk without reaction; Ability to accidentally consume a small amount without reaction; Mild respiratory symptoms; Angioedema; Any adverse effect. | Low risk of bias | Low risk of bias | Low risk of bias | Low risk of bias | Low risk of bias | Low risk of bias |
| Patriarca 1998 | Use of IM epinephrine; Ability to drink cow’s milk without reaction; Ability to accidentally consume a small amount without reaction | High risk of bias | Some concerns | High risk of bias | Some concerns | Some concerns | High risk of bias |
| Salmivesi 2013 | Anaphylaxis; Adverse effect leading to discontinuation of treatment; Severe GI symptoms; Generalized erythema, urticaria and angioedema; Ability to drink milk without a reaction; Ability to accidentally consume a small amount without reaction; Emergency department visit; Death; Angioedema; Lip or mouth pruritus; Any adverse effect. | Low risk of bias | Low risk of bias | Low risk of bias | Low risk of bias | Low risk of bias | Low risk of bias |
| Skripak 2008 | Anaphylaxis; Use of IM epinephrine; Adverse effect leading to discontinuation of treatment; Severe GI symptoms; Generalized erythema, urticaria and angioedema; Ability to drink cow’s milk without reaction; Ability to accidentally consume a small amount without reaction; Death; Mild respiratory symptoms; Lip or mouth pruritus; Any adverse effect. | Low risk of bias | Low risk of bias | Low risk of bias | Low risk of bias | Some concerns | Some concerns |
| Dantzer 2021 | Ability to drink cow's milk and eat dairy products without a reaction; Use of IM epinephrine; Severe gastrointestinal symptoms; Severe respiratory symptoms/wheezing; Generalized urticaria or erythema; Quality of life of children; Quality of life of the caregivers | Some concerns | Low risk of bias | Low risk of bias | Low risk of bias | Low risk of bias | Some concerns |
