# Supplementary material for: World Allergy Organization (WAO) Diagnosis and Rationale for Action against Cow’s Milk Allergy (DRACMA) Guideline update – XIV – Recommendations on CMA immunotherapy
Source: World Allergy Organ J. 2022 Apr 23;15(4):100646. doi: 10.1016/j.waojou.2022.100646 (PMC9061625; doi:10.1016/j.waojou.2022.100646)
Supplement: Multimedia component 9 [file mmc9.docx]

**Supplemental Table 4 - Risk of bias assessment of included non-randomized studies, through Newcastle-Ottawa Scale (NOS).**

| **Author** | **Selection** | | | | **Comparability** | **Outcome** | | | **Score** |
| --- | --- | --- | --- | --- | --- | --- | --- | --- | --- |
|  | Representativeness of the exposed cohort^1^ | Selection of the non-exposed cohort^2^ | Ascertainment of exposure^3^ | Outcome not present at the start^4^ | Comparability of cohorts based on design or analysis^5^ | Ascertainment of outcome^6^ | Follow-up time^7^ | Adequacy of follow up^8^ |  |
| Alvaro 2012^A^ | ***^a^** | NA | ***^a^** | ***^a^** | **-^c^** | ***^a^** | ***^a^** | ***^a^** | 6 |
| Alvarez-Perea 2014 ^B^ | ***^a^** | NA | ***^a^** | ***^a^** | NA | ***^b^** | ***^a^** | ***^b^** | 6 |
| Alves-Correia 2018 | **-^d^** | NA | ***^a^** | ***^a^** | **-^c^** | **-^c^** | ***^a^** | ***^a^** | 4 |
| Aquilante 2018 | **-^d^** | NA | ***^a^** | **-^b^** | **-^c^** | ***^a^** | ***^a^** | ***^b^** | 4 |
| Amat 2017 ^C^ | ***^b^** | NA | ***^a^** | ***^a^** | NA | ***^b^** | ***^a^** | ***^b^** | 6 |
| Arasi 2019 ^D^ | ***^b^** | NA | ***^a^** | ***^a^** | NA | ***^b^** | ***^a^** | ***^b^** | 6 |
| Babaie 2017 | ***^a^** | NA | ***^a^** | ***^a^** | NA | ***^b^** | ***^a^** | ***^b^** | 6 |
| Barbi 2012 ^E^ | ***^a^** | NA | ***^a^** | ***^a^** | NA | **-^c^** | ***^a^** | **-^c^** | 4 |
| Bellon 2018^F^ | ***^b^** | NA | **-^d^** | **-^b^** | NA | **-^c^** | ***^a^** | **-^d^** | 2 |
| Berti 2019 | ***^a^** | NA | ***^b^** | ***^a^** | NA | **-^c^** | ***^a^** | ***^b^** | 5 |
| Blumchen 2010 | ***^a^** | NA | ***^a^** | ***^a^** | NA | ***^a^** | ***^a^** | **-^c^** | 5 |
| Bonito Vitor 2009 | ***^a^** | NA | ***^a^** | ***^a^** | NA | ***^a^** | ***^a^** | ***^b^** | 6 |
| Caminiti 2009B^G^ | ***^a^** | NA | ***^a^** | ***^a^** | NA | ***^a^** | ***^a^** | ***^b^** | 6 |
| Carraro 2012 | ***^a^** | NA | ***^a^** | ***^a^** | NA | ***^a^** | ***^a^** | ***^b^** | 6 |
| Cianferroni 2017 | ***^a^** | NA | ***^a^** | ***^a^** | NA | ***^b^** | ***^a^** | ***^b^** | 6 |
| Dahmane 2020 | ***^a^** | NA | ***^a^** | ***^a^** | NA | ***^b^** | ***^a^** | ***^b^** | 6 |
| De la Fuente 2020 | ***^a^** | NA | NR | ***^a^** | NA | ***^a^** | ***^a^** | ***^b^** | 5 |
| De Schryver 2019 B^H^ | ***^a^** | ***^a^** | ***^a^** | ***^a^** | **-^c^** | ***^b^** | ***^a^** | **-^c^** | 6 |
| Demir 2020^I^ | ***^a^** | NA | ***^a^** | ***^a^** | NA | ***^a^** | ***^a^** | ***^b^** | 6 |
| Ebrahimi 2016^J^ | ***^b^** | NA | ***^a^** | ***^a^** | NA | ***^a^** | ***^a^** | ***^b^** | 6 |
| Echeverría-Zudaire 2016 | ***^b^** | NA | ***^a^** | ***^a^** | NA | ***^a^** | ***^a^** | ***^b^** | 6 |
| El Badawy 2017 | ***^b^** | ***^a^** | ***^a^** | ***^a^** | **-^c^** | ***^b^** | ***^a^** | ***^a^** | 7 |
| Elizur 2015 | ***^a^** | NA | ***^a^** | ***^a^** | NA | ***^b^** | ***^a^** | ***^a^** | 6 |
| Elizur 2016 | ***^a^** | NA | ***^a^** | ***^a^** | NA | ***^b^** | ***^a^** | ***^a^** | 6 |
| Englund 2013 | ***^a^** | NA | ***^a^** | ***^a^** | NA | ***^a^** | ***^a^** | ***^b^** | 6 |
| Epstein Rigbi 2017 | ***^b^** | ***^a^** | ***^a^** | ***^a^** | ***^a^** | ***^b^** | ***^a^** | ***^a^** | 8 |
| Farmakas 2009 | ***^b^** | NA | ***^a^** | ***^b^** | NA | ***^a^** | ***^a^** | ***^b^** | 5 |
| Ferreira Martins 2019 | ***^b^** | NA | ***^a^** | ***^a^** | NA | ***^b^** | ***^a^** | NR | 5 |
| Flor de Lima 2013 | ***^b^** | NA | ***^a^** | ***^a^** | NA | ***^b^** | ***^a^** | NR | 5 |
| Gallucci 2010 | NI | NA | ***^a^** | NR | NA | ***^b^** | ***^a^** | ***^b^** | 4 |
| Garcia-Ara 2013 | ***^b^** | ***^a^** | ***^a^** | ***^a^** | ***^b^** | ***^b^** | ***^a^** | ***^b^** | 9 |
| Garcia-Lirio 2018 | ***^b^** | NA | ***^a^** | ***^a^** | NA | ***^b^** | ***^a^** | ***^a^** | 6 |
| Goldberg 2015^K^ | ***^a^** | NA | ***^a^** | ***^a^** | NA | ***^b^** | ***^a^** | ***^a^** | 6 |
| Gómez García 2019 | ***^b^** | NA | ***^a^** | ***^a^** | NA | ***^b^** | ***^a^** | ***^b^** | 6 |
| Gonzales Jimenez 2013 | ***^b^** | NA | ***^a^** | ***^a^** | NA | ***^b^** | ***^a^** | ***^a^** | 6 |
| Gruzelle 2020 | ***^a^** | NA | ***^a^** | ***^a^** | NA | ***^b^** | NR | NR | 4 |
| Hague 2014 | ***^a^** | NA | ***^a^** | ***^a^** | NA | ***^b^** | ***^a^** | ***^a^** | 6 |
| Hayashi 2011 | ***^a^** | NA | ***^a^** | ***^a^** | NA | ***^b^** | **-^b^** | NR | 5 |
| Hirayama 2016 | ***^a^** | ***^a^** | ***^a^** | ***^a^** | ***^a^** | ***^b^** | ***^a^** | NR | 7 |
| Indiveri 2017 | NI | NA | **-^d^** | **-^d^** | NA | **-^d^** | **-^b^** | ***^b^** | 1 |
| Kameda 2017 | ***^a^** | NA | ***^a^** | **-^d^** | NA | ***^b^** | ***^a^** | ***^a^** | 5 |
| Kando 2012 | ***^b^** | NA | ***^a^** | ***^a^** | NA | ***^b^** | ***^a^** | ***^a^** | 6 |
| Kaneko 2010 | ***^b^** | NA | ***^a^** | ***^a^** | NA | ***^b^** | **-^b^** | ***^b^** | 6 |
| Katz 2015 | ***^a^** | NA | ***^a^** | **-^d^** | NA | **-^c^** | ***^a^** | ***^a^** | 4 |
| Kauppila 2019 | ***^b^** | **-^c^** | ***^a^** | **-^c^** | ***^a^** | **-^c^** | ***^a^** | **-^c^** | 5 |
| Ke 2019 | ***^b^** | NA | ***^a^** | ***^a^** | NA | ***^b^** | ***^a^** | ***^a^** | 6 |
| Keet 2013 | NI | NA | **-^c^** | **-^c^** | NA | **-^c^** | ***^a^** | ***^b^** | 2 |
| Kivisto 2015 | ***^a^** | NA | ***^a^** | ***^a^** | NA | ***^b^** | ***^a^** | ***^a^** | 6 |
| Kloepfer 2008 | ***^a^** | ***^a^** | ***^a^** | ***^a^** | NR | ***^a^** | ***^a^** | ***^a^** | 7 |
| Kuitunen 2015 | ***^a^** | NA | ***^a^** | ***^a^** | NA | ***^b^** | ***^a^** | ***^a^** | 6 |
| Kunizaki 2018 | NI | NA | ***^a^** | ***^a^** | NA | ***^a^** | ***^a^** | ***^a^** | 5 |
| Levy 2014^L^ | ***^a^** | NA | ***^a^** | ***^a^** | NA | ***^b^** | ***^a^** | ***^b^** | 6 |
| Lomas 2014 | NI | NA | ***^a^** | ***^a^** | NA | NR | ***^a^** | ***^a^** | 4 |
| Longo 2012 | ***^a^** | NA | ***^a^** | ***^a^** | NA | ***^b^** | ***^a^** | ***^a^** | 6 |
| Luyt 2014^M^ | ***^a^** | NA | ***^a^** | ***^a^** | NA | ***^b^** | ***^a^** | ***^b^** | 6 |
| Mack 2017 | NI | NA | ***^a^** | ***^a^** | NA | ***^b^** | ***^a^** | ***^a^** | 5 |
| Manabe 2019 | NI | NA | **-^c^** | **-^c^** | NA | **-^c^** | ***^a^** | ***^a^** | 2 |
| Mantyla 2018^N^ | NI | NA | ***^a^** | ***^a^** | NA | ***^b^** | ***^a^** | **-^c^** | 4 |
| Martinez-Botas 2015 | ***^a^** | ***^a^** | ***^a^** | ***^a^** | **-^c^** | ***^a^** | ***^a^** | ***^b^** | 9 |
| Meglio 2004^O^ | ***^a^** | NA | **-^b^** | ***^a^** | NA | **-^c^** | ***^a^** | ***^a^** | 4 |
| Miura 2018 | **-^d^** | **-^c^** | **-^d^** | ***^a^** | **-^c^** | ***^b^** | ***^a^** | ***^a^** | 4 |
| Miura 2021 | NI | NA | **-^c^** | ***^a^** | NA | **-^c^** | ***^a^** | NR | 2 |
| Moreno 2019 | ***^a^** | NA | **-^d^** | **-^d^** | NA | **-^c^** | **-^b^** | ***^a^** | 2 |
| Mori 2010 | ***^b^** | ***^a^** | ***^a^** | ***^a^** | ***^a^** | ***^b^** | ***^a^** | NR | 7 |
| Mori 2017 | ***^a^** | NA | ***^a^** | ***^a^** | NA | **-^c^** | ***^a^** | ***^a^** | 5 |
| Mota 2018 | ***^b^** | NA | **-^b^** | ***^a^** | NA | **-^c^** | ***^a^** | ***^a^** | 4 |
| NCT01162473 | ***^a^** | NA | **-^d^** | ***^a^** | NA | ***^a^** | ***^a^** | ***^b^** | 4 |
| Nachshon 2020 | ***^b^** | NA | ***^a^** | ***^a^** | NA | ***^b^** | ***^a^** | ***^a^** | 6 |
| Nagakura 2020 | ***^b^** | NA | ***^a^** | ***^a^** | NA | ***^b^** | ***^a^** | ***^a^** | 6 |
| Nagaraju 2020 | NI | NA | ***^a^** | ***^a^** | NA | ***^b^** | ***^a^** | ***^a^** | 5 |
| Natsume 2019 | ***^b^** | NA | ***^a^** | ***^a^** | NA | ***^a^** | ***^a^** | NR | 5 |
| Navakova 2011 | NI | NA | ***^a^** | ***^a^** | NA | ***^b^** | ***^a^** | ***^a^** | 5 |
| Ogura 2012 A | **-^d^** | **-^c^** | **-^d^** | ***^a^** | **-^c^** | ***^b^** | ***^a^** | ***^a^** | 4 |
| Ogura 2012 B | **-^d^** | **-^c^** | **-^d^** | ***^a^** | **-^c^** | ***^b^** | ***^a^** | **-^d^** | 3 |
| Ogura 2020 A | ***^a^** | NA | ***^a^** | ***^a^** | NA | ***^b^** | ***^a^** | ***^b^** | 6 |
| Ogura 2020 B | ***^a^** | NA | ***^a^** | ***^a^** | NA | ***^b^** | ***^a^** | **-^c^** | 5 |
| Ono 2018 | **-^d^** | NA | **-^d^** | ***^a^** | NA | **-^d^** | ***^a^** | **-^d^** | 2 |
| Ortega-Camarero 2012 | ***^b^** | NA | ***^a^** | ***^a^** | NA | ***^b^** | **-^b^** | ***^a^** | 5 |
| Paassilta 2016^P^ | ***^a^** | NA | **-^d^** | ***^a^** | NA | **-^c^** | ***^a^** | ***^a^** | 4 |
| Pajno 2013^Q^ | ***^a^** | NA | ***^a^** | ***^a^** | NA | ***^b^** | ***^a^** | ***^b^** | 6 |
| Palhinha 2019 | ***^b^** | NA | NR | ***^a^** | NA | ***^b^** | ***^a^** | ***^b^** | 5 |
| Patriarca 2002 | ***^a^** | **-^c^** | **-^d^** | ***^a^** | **-^c^** | **-^c^** | ***^a^** | ***^b^** | 4 |
| Patriarca 2007 | ***^a^** | **-^c^** | **-^d^** | ***^a^** | **-^c^** | ***^a^** | ***^a^** | ***^b^** | 5 |
| Perezabad 2017 | ***^a^** | NA | **-^d^** | ***^a^** | NA | **-^c^** | ***^a^** | ***^b^** | 4 |
| Petrakis 2019 | ***^b^** | NA | ***^a^** | ***^a^** | NA | ***^b^** | ***^a^** | ***^a^** | 6 |
| Poza-Guedes 2015 | ***^b^** | NA | ***^a^** | ***^a^** | NA | ***^b^** | ***^a^** | ***^a^** | 6 |
| Reche 2011 | **-^d^** | **-^c^** | **-^d^** | ***^a^** | ***^a^** | **-^d^** | ***^a^** | ***^a^** | 4 |
| Rios 2018 | ***^b^** | NA | NR | ***^a^** | NA | ***^b^** | ***^a^** | NR | 4 |
| Rodriguez-Alvarez 2010 | ***^b^** | NA | ***^a^** | ***^a^** | NA | ***^b^** | ***^a^** | ***^a^** | 6 |
| Saltzman 2012 | ***^b^** | NA | ***^a^** | ***^a^** | NA | ***^b^** | ***^a^** | ***^a^** | 6 |
| Sanchez-Garcia 2012 | ***^b^** | NA | ***^a^** | ***^a^** | NA | ***^b^** | ***^a^** | ***^a^** | 6 |
| Sato 2019 | **-^d^** | NA | **-^d^** | ***^a^** | NA | **-^d^** | **-^c^** | ***^a^** | 2 |
| Savilahti 2014 A | ***^a^** | NA | **-^c^** | ***^a^** | NA | **-^c^** | ***^a^** | ***^b^** | 4 |
| Savilahti 2014 B | ***^a^** | NA | **-^c^** | ***^a^** | NA | **-^c^** | **-^c^** | ***^b^** | 3 |
| Shibata 2013 | ***^a^** | NA | ***^a^** | ***^a^** | NA | ***^b^** | ***^a^** | ***^a^** | 6 |
| Skripak 2008 B^R^ | ***^a^** | ***^a^** | **-^d^** | ***^a^** | ***^a^** | ***^a^** | ***^a^** | ***^b^** | 7 |
| Staden 2008 | ***^a^** | NA | **-^d^** | ***^a^** | NA | ***^a^** | **-^b^** | ***^a^** | 5 |
| Sugimoto 2012 | NI | NA | NR | NR | NA | ***^b^** | ***^a^** | ***^a^** | 3 |
| Sugiura 2020 | ***^b^** | ***^a^** | ***^a^** | ***^a^** | ***^a^** | ***^b^** | ***^a^** | ***^a^** | 9 |
| Takahashi 2013 | ***^b^** | NA | ***^a^** | ***^a^** | NA | ***^b^** | ***^a^** | ***^a^** | 6 |
| Takahashi 2016 | ***^a^** | ***^a^** | **-^d^** | ***^a^** | ***^a^** | ***^b^** | ***^a^** | ***^a^** | 7 |
| Takaoka 2020 | ***^b^** | NA | ***^a^** | ***^a^** | NA | ***^b^** | ***^a^** | ***^a^** | 6 |
| Valenca 2016 | NA | NA | NA | NA | NA | NA | NA | NA | NA |
| Vazquez 2011 | NI | NA | ***^a^** | **-^b^** | NA | **-^c^** | ***^a^** | ***^b^** | 3 |
| Vazquez-Ortiz 2013 | ***^a^** | NA | ***^a^** | ***^a^** | NA | **-^c^** | ***^a^** | ***^b^** | 5 |
| Wasserman 2018 | **-^d^** | NA | **-^d^** | **-^b^** | NA | **-^d^** | **-^c^** | **-^d^** | 0 |
| Weinbrand-Goichberg 2016 | NA | NA | NA | NA | NA | NA | NA | NA | NA |
| Wood 2016^S^ | ***^a^** | NA | ***^b^** | ***^a^** | NA | ***^a^** | ***^a^** | ***^a^** | 6 |
| Yanagida 2010 | ***^b^** | NA | ***^a^** | ***^a^** | NA | ***^b^** | ***^a^** | ***^a^** | 6 |
| Yanagida 2015 | ***^a^** | ***^a^** | **-^d^** | ***^a^** | **-^c^** | ***^b^** | ***^a^** | ***^a^** | 6 |
| Yonekura Anagusko 2019 | **-^d^** | NA | **-^d^** | ***^a^** | NA | **-^d^** | **-^c^** | **-^d^** | 1 |
| Zapatero 2008 | ***^a^** | NA | ***^b^** | ***^a^** | NA | **-^c^** | ***^a^** | ***^b^** | 5 |
| Zhao 2019 A | NA | NA | NA | NA | NA | NA | NA | NA | NA |
| Zhao 2019 B | NA | NA | NA | NA | NA | NA | NA | NA | NA |

^1^a) Truly representative*, b) Somewhat representative*, c) Selected group of users, d) No description of the derivation of the cohort

^2^a) Drawn from the same community as the exposed cohort*, b) Drawn from a different source, c) No description

^3^a) Secure record*, b) Structured interview, c) Written self-report, d) No description

^4^a) Yes*, b) No

^5^ Study controls for a) at least one of the following factors: age, sex, ethnicity, immune comorbidities*, b) at least one other factor*, c) no factors

^6^a) Independent blind assessment*, b) Record linkage*, c) Self-report, d) No description

^7^a) Follow-up time was long enough for outcome to occur *, b) Follow-up time was not long enough for outcome to occur, c) Not reported or Unclear

^8^a) Complete follow-up - all subjects accounted for*, b) Subjects lost to follow-up unlikely to introduce bias (≥80%)*, c) Follow-up rate < 80% and no description of those lost, d) No statement.

^A^ Same study as Alvaro 2011

^B^ Same study as Alvarez-Perea 2014 A-B

^C^ Same study as Amat 2017 A-B

^D^ Same study as Arasi 2019 A-B

^E^ Same study as Barbi 2011

^F^ Same study as Bellon 2018 A-B-C

^G^ Same study as Caminiti 2009 A

^H^ Same study as Torabi 2016; Torabi 2017; De Schryver 2019 A

^I^ Same study as Demir 2017; Demir 2014; Demir 2012

^J^ Same study as Ebrahimi 2017

^K^ Same study as Goldberg 2014

^L^ Same study as Goldberg 2017; Goldberg 2019; Levy 2012

^M^ Same study as Luyt 2013

^N^ Same study as Kauppi 2014

^O^ Same study as Meglio 2008

^P^ Follow up of Salmivesi 2013

^Q^ Same study as Pajno 2014

^R^ Same study as Skripak 2008 A; Narisety 2009

^S^ Same study as Fischmeyer-Guerrerio 2017; Kim 2014; Suarez-Farinas 2018; Suarez-Farinas 2016

NA: not applicable.

NI: not enough information

NR: not reported
