# Supplementary material for: World Allergy Organization (WAO) Diagnosis and Rationale for Action against Cow’s Milk Allergy (DRACMA) Guideline update – XIV – Recommendations on CMA immunotherapy
Source: World Allergy Organ J. 2022 Apr 23;15(4):100646. doi: 10.1016/j.waojou.2022.100646 (PMC9061625; doi:10.1016/j.waojou.2022.100646)
Supplement: Multimedia component 11 [file mmc11.docx]

**Table 2 A: Evidence profile from the included controlled randomized trials**

**Question**: OIT with baked milk compared to no OIT in patients with IgE-CMA who do not tolerate baked milk

| **Certainty assessment** | | | | | | | **№ of patients** | | **Effect** | | **Certainty** | **Importance** |
| --- | --- | --- | --- | --- | --- | --- | --- | --- | --- | --- | --- | --- |
| **№ of studies** | **Study design** | **Risk of bias** | **Inconsistency** | **Indirectness** | **Imprecision** | **Other considerations** | **Baked Milk OIT** | **No OIT** | **Relative (95% CI)** | **Absolute (95% CI)** |  |  |
| **Ability to drink cow's milk and eat dairy products without a reaction (follow-up: 12 months)** | | | | | | | | | | | | |
| 1^1^ | randomised trials | serious ^a^ | not serious | not serious | serious ^b^ | none | 11/15 (73.3%) | 0/15 (0.0%) | **RR 23.00** (1.48 to 358.00) | **0 fewer per 100** (from 0 fewer to 0 fewer) | ⨁⨁◯◯ Low | CRITICAL |
| **Use of IM epinephrine (follow-up: 12 months)** | | | | | | | | | | | | |
| 1^1^ | randomised trials | serious ^a^ | not serious | not serious | very serious ^c^ | none | 3/15 (20.0%) | 1/15 (6.7%) | **RR 3.00** (0.35 to 25.68) | **13 more per 100** (from 4 fewer to 100 more) | ⨁◯◯◯ Very low | CRITICAL |
| **Severe gastrointestinal symptoms (follow-up: 12 months)** | | | | | | | | | | | | |
| 1^1^ | randomised trials | serious ^a^ | not serious | not serious | very serious ^c^ | none | 1/15 (6.7%) | 1/15 (6.7%) | **RR 1.00** (0.07 to 14.53) | **0 fewer per 100** (from 6 fewer to 90 more) | ⨁◯◯◯ Very low | CRITICAL |
| **Severe respiratory symptoms/wheezing (follow-up: 12 months)** | | | | | | | | | | | | |
| 1^1^ | randomised trials | serious ^a^ | not serious | not serious | very serious ^c^ | none | 1/15 (6.7%) | 2/15 (13.3%) | **RR 0.50** (0.05 to 4.94) | **7 fewer per 100** (from 12 fewer to 52 more) | ⨁◯◯◯ Very low | CRITICAL |
| **Generalized urticaria or erythema (follow-up: 12 months)** | | | | | | | | | | | | |
| 1^1^ | randomised trials | serious ^a^ | not serious | not serious | very serious ^c^ | none | 2/15 (13.3%) | 1/15 (6.7%) | **RR 2.00** (0.20 to 19.78) | **7 more per 100** (from 5 fewer to 100 more) | ⨁◯◯◯ Very low | CRITICAL |
| **Quality of life of children (follow-up: 12 months)** | | | | | | | | | | | | |
| 1^1^ | randomised trials | serious ^a^ | not serious | not serious | very serious ^d^ | none | Most of OIT patients experienced an overall improvement in QoL. The only age group in which a between-group comparison could be done was children between 8-12 years of age, surveyed through the FAQOL-CF questionnaire (OIT: 5; Placebo: 5). The results suggest that patients in the OIT arm, were more likely to have a QoL improvemement (>0.5 MCID). | | | | ⨁◯◯◯ Very low | IMPORTANT |
| **Quality of life of the caregivers (follow-up: 12 months)** | | | | | | | | | | | | |
| 1^1^ | randomised trials | serious ^a^ | not serious | not serious | very serious ^d^ | none | The parents of 26 patients (OIT: 12; Placebo: 14) were surveyed through the FAQOL-PF questionnaire. The results highlighted no overall difference between groups, but the parents in the placebo arm appeared more likely to experience an improvement (>0.5 MCID) in the emotional domain. | | | | ⨁◯◯◯ Very low | IMPORTANT |

**CI:** confidence interval; **MD:** mean difference; **RR:** risk ratio

#### Explanations

a. RoB.2 some concerns for risk of bias

b. Few events among only 30 patients and the effect estimate crosses several thresholds of effect size

c. Few events among only 30 patients and the effect estimate does not distinguish between appreciable benefit or harm.

d. Few events (>0.5 MCID) among a limited number of patients.

#### References

1.Dantzer J, Dunlop J, Psoter KJ, Keet C, Wood R. Efficacy and safety of baked milk oral immunotherapy in children with severe milk allergy: A randomized, double-blind, placebo-controlled phase 2 trial. Journal of Allergy and Clinical Immunology 2021

**Table 2 B: Evidence Profile from the included non-randomized studies**

**Question**: OIT with baked milk compared to no OIT in patients with IgE-CMA who do not tolerate baked milk

| **Certainty assessment** | | | | | | | **Impact** | **Certainty** | **Importance** |
| --- | --- | --- | --- | --- | --- | --- | --- | --- | --- |
| **№ of studies** | **Study design** | **Risk of bias** | **Inconsistency** | **Indirectness** | **Imprecision** | **Other considerations** |  |  |  |
| **Anaphylaxis (follow up: 17 months)** | | | | | | | | | |
| 1 ^1^ | observational studies | serious ^a^ | not serious | not serious | very serious ^b^ | publication bias strongly suspected ^c^ | 1/20 patients (**5%**) | ⨁◯◯◯ VERY LOW | CRITICAL |
| **Use of IM epinephrine (mean follow up: 14 months)** | | | | | | | | | |
| 2 ^1,2^ | observational studies | serious ^a^ | not serious | not serious | very serious ^d^ | publication bias strongly suspected ^c^ | 3/15 (**20%**) in one study and 1/20 (**5%**) in another study. | ⨁◯◯◯ VERY LOW | CRITICAL |
| **Discontinuation of treatment due to adverse effects and/or symptoms (mean follow up: 14 months)** | | | | | | | | | |
| 2 ^1,2^ | observational studies | serious ^a^ | not serious | not serious | very serious ^d^ | publication bias strongly suspected ^c^ | 2/15 (**13%**) in one study and 4/20 (**20%**) in another study. | ⨁◯◯◯ VERY LOW | CRITICAL |
| **Severe gastrointestinal symptoms (mean follow up: 14 months)** | | | | | | | | | |
| 2 ^1,2^ | observational studies | serious ^a^ | not serious | serious ^e^ | very serious ^d^ | publication bias strongly suspected ^c^ | 5/15 (**33%**) in one study and 3/20 (**15%**) in another study. | ⨁◯◯◯ VERY LOW | CRITICAL |
| **Severe respiratory symptoms/wheezing (mean follow up: 14 months)** | | | | | | | | | |
| 2 ^1,2^ | observational studies | serious ^a^ | not serious | serious ^e^ | very serious ^d^ | publication bias strongly suspected ^c^ | 8/15 (**53%**) in one study and 2/20 (**10%**) in another study. | ⨁◯◯◯ VERY LOW | CRITICAL |
| **Generalized urticaria or erythema (follow up: 12 months)** | | | | | | | | | |
| 1 ^2^ | observational studies | serious ^a^ | not serious | serious ^f^ | very serious | publication bias strongly suspected ^c^ | 5/15 patients (**33%**) | ⨁◯◯◯ VERY LOW | CRITICAL |
| **Ability to drink cow's milk and eat dairy products without a reaction (mean follow up: 14 months; assessed with: passing a supervised graded food challenge with >254 ml of fresh cow's milk or ability to eat 1.3 g of baked milk)** | | | | | | | | | |
| 2 ^1,2^ | observational studies | serious ^a^ | not serious | not serious | very serious ^d^ | publication bias strongly suspected ^c^ | 4/15 (**27%**) in one study and 5/20 (**25%**) in another study. | ⨁◯◯◯ VERY LOW | CRITICAL |

**CI:** Confidence interval

#### Explanations

a. No control group (series of cases). Any inference requires implicit comparison.

b. Only one event

c. One additional study (Lazzarotto 2013, Lazzarotto 2014) has been completed and published only as a conference abstract with no information about the outcomes.

d. Few events among only 35 patients

e. Studies did not report how severe were the symptoms.

f. Most studies reported urticaria without mentioning its range or severity.

#### References

1. Gruzelle, V., Juchet, A., Martin-Blondel, A., Michelet, M., Chabbert-Broue, A., Didier, A.. Benefits of baked milk oral immunotherapy in French children with cow's milk allergy. Pediatric Allergy and Immunology.; 2020.

2. Goldberg, M. R., Nachshon, L., Appel, M. Y., Elizur, A., Levy, M. B., Eisenberg, E., Sampson, H. A., Katz, Y.. Efficacy of baked milk oral immunotherapy in baked milk-reactive allergic patients. Journal of Allergy & Clinical Immunology; Dec 2015.
